# Mathematical characterization of population dynamics in breast cancer cells treated with doxorubicin

**DOI:** 10.3389/fmolb.2022.972146

**Published:** 2022-09-12

**Authors:** Emily Y. Yang, Grant R. Howard, Amy Brock, Thomas E. Yankeelov, Guillermo Lorenzo

**Affiliations:** ^1^ Oden Institute for Computational Engineering and Sciences, The University of Texas at Austin, Austin, TX, United States; ^2^ Department of Biomedical Engineering, The University of Texas at Austin, Austin, TX, United States; ^3^ Livestrong Cancer Institutes, Dell Medical School, The University of Texas at Austin, Austin, TX, United States; ^4^ Interdisciplinary Life Sciences Program, The University of Texas at Austin, Austin, TX, United States; ^5^ Department of Diagnostic Medicine, The University of Texas at Austin, Austin, TX, United States; ^6^ Department of Oncology, The University of Texas at Austin, Austin, TX, United States; ^7^ Department of Imaging Physics, The University of Texas MD Anderson Cancer Center, Houston, TX, United States; ^8^ Department of Civil Engineering and Architecture, University of Pavia, Pavia, Italy

**Keywords:** mathematical oncology, mathematical modeling, population dynamics, chemotherapy, drug resistance, breast cancer, time-resolved microscopy

## Abstract

The development of chemoresistance remains a significant cause of treatment failure in breast cancer. We posit that a mathematical understanding of chemoresistance could assist in developing successful treatment strategies. Towards that end, we have developed a model that describes the cytotoxic effects of the standard chemotherapeutic drug doxorubicin on the MCF-7 breast cancer cell line. We assume that treatment with doxorubicin induces a compartmentalization of the breast cancer cell population into surviving cells, which continue proliferating after treatment, and irreversibly damaged cells, which gradually transition from proliferating to treatment-induced death. The model is fit to experimental data including variations in drug concentration, inter-treatment interval, and number of doses. Our model recapitulates tumor cell dynamics in all these scenarios (as quantified by the concordance correlation coefficient, CCC > 0.95). In particular, superior tumor control is observed with higher doxorubicin concentrations, shorter inter-treatment intervals, and a higher number of doses (*p* < 0.05). Longer inter-treatment intervals require adapting the model parameterization after each doxorubicin dose, suggesting the promotion of chemoresistance. Additionally, we propose promising empirical formulas to describe the variation of model parameters as functions of doxorubicin concentration (CCC > 0.78). Thus, we conclude that our mathematical model could deepen our understanding of the cytotoxic effects of doxorubicin and could be used to explore practical drug regimens achieving optimal tumor control.

## Introduction

Breast cancer is the most common cancer among women worldwide and the leading cause of cancer death in over 100 countries (Sung et al., 2021). Chemotherapy is a primary component of cancer treatment and options have both advanced and increased considerably in recent years (Anampa et al., 2015). However, the development of chemoresistance, and resulting tumor recurrence, remains a common cause of treatment failure and a primary cause of cancer death (Holohan et al., 2013; Longley and Johnston, 2005). Indeed, for a standard chemotherapy drug such as doxorubicin, breast cancer patients may develop chemoresistance within just 6–10 months (Rivera and Gomez, 2010; O’Shaughnessy, 2005).

From a biological perspective, the development of chemoresistance is governed by many complex mechanisms, such as treatment-induced genetic and epigenetic alterations, altered metabolic states, and adaptive responses of the tumor microenvironment (Easwaran et al., 2014; Zhao, 2016; Dong et al., 2018; Ji et al., 2019). Tumor cells can also possess an intrinsic phenotypic or genetic resistance that can render the therapy ineffective even before acquired chemoresistance develops (Harris et al., 2007; Bernard et al., 2009; Campbell et al., 2018). Moreover, the existence of intratumoral heterogeneity and its role in tumor regrowth have become increasingly recognized, as the presence of even a minor subpopulation of drug-resistant cells can give rise to tumor relapse (Easwaran et al., 2014; Polyak, 2011; Alizadeh et al., 2015; Sun and Yu, 2015). Furthermore, phenotype switching, in which tumor cells swap between varying degrees of drug-resistant and drug-sensitive phenotypes, can enable the establishment of more permanent chemoresistance mechanisms that hinder complete tumor eradication (Easwaran et al., 2014; Meacham and Morrison, 2013; Echeverria et al., 2019; Kumar et al., 2019). Considering the complex biological processes underlying chemoresistance development, we believe that a robust framework is needed to comprehensively integrate the growing knowledge of this phenomenon and guide future research efforts. To this end, experimentally-validated mathematical models of chemoresistance mechanisms could be a potent tool in understanding the dynamics of overall tumor drug response. The description of cancer growth and therapeutic response by leveraging mechanistic mathematical models is a rich field known as mathematical oncology (Yankeelov et al., 2013; Rockne et al., 2019; Lorenzo et al., 2022). This approach has already shown promise in characterizing breast cancer growth and treatment response in both the preclinical and clinical settings (Atuegwu et al., 2013; Pascal et al., 2013; Weis et al., 2015; Zhang et al., 2015; Geng et al., 2017; Palmer and Sorger, 2017; Jarrett et al., 2018; Jarrett et al., 2020a).

There are several mechanistic approaches to mathematically describe chemoresistance (Sun and Hu, 2018; Craig et al., 2019; Craig et al., 2021; Hori et al., 2021), with the original theoretical models dating back more than two decades (Panetta, 1997; Panetta, 1998). The standard strategy consists of defining a multicompartmental tumor cell population including one or multiple species of both drug-resistant and drug-sensitive cells, which evolve and interact over time following a set of ordinary differential equations, or over both space and time according to a set of partial differential equations (Craig et al., 2019; Craig et al., 2021; Hori et al., 2021; Jackson and Byrne, 2000; Greene et al., 2019; Kim et al., 2021; Strobl et al., 2021; Howard et al., 2022). Alternatively, Sun et al. (2016) utilized a stochastic, multiscale model that incorporated heterogeneous population dynamics with drug pharmacokinetics and microenvironment contributions to drug resistance in melanoma patients. Furthermore, Pisco et al. (2013) and Álvarez-Arenas et al. (2019) applied the evolutionary theories of Darwinian selection and Lamarckian induction to guide their modeling of drug resistance in leukemia cells and non-small cell lung carcinoma, respectively. However, despite these promising studies, there is a still a dearth of experimentally-validated mechanistic models of chemoresistance in breast cancer, with which we could test alternative biological hypotheses to ultimately enhance chemotherapeutic strategies for individual patients. For instance, Chapman et al. (2019) developed a model integrating phenotypic switching of cell differentiation states and tumor heterogeneity to characterize therapeutic escape in the triple-negative subtype, but the empirical validation of their model predictions currently remains limited. Additionally, *in vitro* studies usually label cell lines as homogeneously drug-resistant or drug-sensitive and assume a static drug sensitivity (AbuHammad and Zihlif, 2013; Gottesman, 2002), which overlooks the existence of intratumoral heterogeneity and transient drug resistance. Moreover, preclinical studies often assess tumor cell death at a single time point 24–72 h post-treatment (Martins et al., 2018; Chung et al., 2020; Low et al., 2021). This experimental setting does not enable the characterization of long-term tumor drug responses and, hence, the development of drug-induced chemoresistance.

Here, we present a mechanistic model to describe the dynamics of drug response and chemoresistance development in MCF-7 breast cancer cells treated with doxorubicin, which we fit to time-resolved microscopy measurements of tumor cell number subjected to diverse therapeutic plans over long experimental times (>8 days). Doxorubicin is a cytotoxic anthracycline drug that is extensively used in chemotherapeutic regimens for breast cancer (Jarrett et al., 2020a; Zardavas and Piccart, 2015; Waks and Winer, 2019). As a cytotoxic drug, treatment with doxorubicin primarily induces tumor cell death, but this therapeutic effect may also be preceded by cell cycle arrest (Anampa et al., 2015; Howard et al., 2022; Gewirtz, 1999; Carvalho et al., 2009; McKenna et al., 2017). Our work continues the first efforts of Howard et al. in studying doxorubicin resistance in breast cancer cell populations by leveraging several experimentally-informed mechanistic models (Howard et al., 2022; Howard et al., 2018). While Howard et al. originally proposed multiple models to characterize this phenomenon and selected the best of them for each dataset, we have developed a single model that can be extended for multiple drug doses. To incorporate intratumoral heterogeneity, we assume that the cytotoxic action of doxorubicin treatment induces a compartmentalization of the breast cancer cell population into two subgroups: surviving cells and irreversibly damaged cells, which will ultimately die due to doxorubicin action. We further assume that the surviving cells continue proliferating after exposure to doxorubicin, while the irreversibly damaged cells progressively transition from proliferation to drug-induced death. Hence, the eventual development of chemoresistance will be driven by the surviving cells. Importantly, the model compartmentalization ultimately results from the underlying distribution of diverse drug sensitivity phenotypes in the tumor cell population and its changes after the delivery of each doxorubicin dose (Howard et al., 2022; Pisco et al., 2013; Álvarez-Arenas et al., 2019). To accommodate potentially significant treatment-induced variations in the underlying spectrum of drug resistance phenotypes, we investigate an adaptive parametrization of our model with each doxorubicin dose. Hence, drug sensitivity in the tumor cell population is assumed to be dynamic with time, thereby accounting for tumor cell plasticity (Easwaran et al., 2014; Meacham and Morrison, 2013; Echeverria et al., 2019; Kumar et al., 2019). Additionally, our model is fit to the same time-resolved microscopy experiments used in Howard et al. (2022), in which breast cancer cells were subjected to doxorubicin treatments varying in either drug concentration, inter-treatment interval, or the number of doses. Our results show that our proposed model can fit the data observed in all three scenarios with remarkable accuracy. We have also analyzed the model parameter trends for each experiment and built empirical parameter formulas as functions of doxorubicin concentration, which may provide further insight into the development of chemoresistance.

The remainder of this work is organized as follows. First, given that we utilize the time-resolved microscopy data previously collected by Howard et al. (2022), we briefly outline their acquisition and preprocessing procedures. We also describe the derivation of the model and explain the numerical and statistical methods leveraged in this study. We then present the results from our model fittings for each of the three aforementioned experimental scenarios and analyze the corresponding quality of fit and trends in model parameters. To conclude, we discuss the main implications from our work, its limitations, and future directions.

## Methods

### Data acquisition and preprocessing

The experimental data leveraged in this study were fully obtained from Howard et al. (2022). In the following, we provide only the salient details of the data acquisition and preprocessing procedures presented in Howard et al. (2022) and directly relevant to our study, to which we added a final outlier assessment.

#### Cell culture

MCF-7 human breast cancer cells (ATCC HTB-22) were cultured in Minimum Essential Media (Gibco) supplemented with 10% fetal bovine serum (Gibco) and 1% penicillin-streptomycin (Gibco). Cells were maintained at 37°C with 5% CO_2_. A stable fluorescent cell line expressing constitutive EGFP with a nuclear localization signal (MCF7-EGFPNLS1) was established to aid in the automated cell quantification of the time resolved microscopy measurements (Howard et al., 2022; Howard et al., 2018). Genomic integration of the EGFP expression cassette was accomplished by leveraging the Sleeping Beauty transposon system. The EGFP-NLS sequence was obtained as a gBlock (IDT) and cloned into the optimized Sleeping Beauty transfer vector pSBbi-Neo (which was a gift from Eric Kowarz, Addgene plasmid #60525) (Kowarz et al., 2015). To mediate genomic integration, this two-plasmid system consisting of the transfer vector containing the EGFP-NLS sequence and the pCMV(CAT)T7-SB100 plasmid containing the Sleeping Beauty transposase was co-transfected into the MCF-7 population utilizing Lipofectamine 2000 (mCMV(CAT)T7-SB100 was a gift from Zsuzsanna Izsvak, Addgene plasmid #34879) (Mátés et al., 2009). After gene integration with Sleeping Beauty transposase, EGFP + cells were collected by fluorescence activated cell sorting and maintained in media supplemented with 200 ng/ml G418 (Caisson Labs) in place of penicillin-streptomycin.

#### Doxorubicin response experiments

Cells were seeded in a 96-well plate at a target density of 2,000 cells/well and grown for approximately 48 h to allow for cell adhesion and recovery from passaging. An IncuCyte S2 Live Cell Analysis System (Essen/Sartorius, Goettingen, Germany) was used to collect fluorescent and phase contrast images every 2–4 h. Images were collected for periods of 21–56 days to ensure that cultures in which cells recover after exposure to doxorubicin were able to display logistic growth. Doxorubicin treatment was prepared by reconstituting doxorubicin hydrochloride (Cayman Chemical 15,007, Ann Arbor, Michigan) in water and mixing it with 100 µl of growth media at 2× the target concentration, which was then added to each well of the plate. The drug-containing media was then replaced with fresh growth media after 24 h. Three experiment types were run, in which either the doxorubicin concentration, the inter-treatment interval, or the number of doses was varied (see Table 1). Each doxorubicin concentration was tested in *n* = 6 replicates, while each inter-treatment interval and number of doses was tested in *n* = 12 replicates.

**TABLE 1:** Experimental conditions. In Experiment 1, one dose of doxorubicin was delivered at concentrations varying from 10 to 300 nM (*n* = 6). In Experiment 2, two doses of 75 nM doxorubicin were delivered at inter-treatment intervals varying from 0 to 16 days (*n* = 12). In Experiment 3, one to five doses of 75 nM doxorubicin were delivered at either 2-day or 2-week inter-treatment intervals (*n* = 12).

Experiment	Concentration [nM]	Inter-treatment Interval [d]	Number of Doses	Number of Replicates
1	0, 10, 20, 35, 50, 75, 100, 125, 150, 300	-	1	6
2	75	0, 2, 4, 6, 8, 10, 12, 14, 16	2	12
3	75	2 or 14	1, 2, 3, 4, 5	12

#### Image analysis

Using IncuCyte’s integrated software, the quantification of total tumor cell counts was performed on the fluorescent images using the green fluorescence channel. Individual cells were consistently resolved using standard image analysis techniques of background subtraction, followed by thresholding, edge detection, and minimum area filtering. The phase contrast images were consulted in parallel to aid the validation of image analysis (Howard et al., 2022; Howard et al., 2018).

#### Data truncation

The tumor cell time courses extracted from some wells did not provide meaningful data throughout the entire time course due to a variety of reasons. These included the cell population growing to confluence and fluctuating with feeding cycles, being disturbed during media replenishment, or growing three-dimensionally resulting in cells overlapping each other and thereby compromising the ability to accurately quantify cell numbers. Thus, for each dataset, the estimated cell number was truncated either just prior to reaching confluence, when the cell number dropped more than 50% due to media handling, or when repeated discontinuities were observed in the time course data.

#### Data normalization

For smaller discontinuities in which less than 50% of the cells were lost due to media handling, the data was normalized by dividing the cell number at time points before the discontinuity by a constant α (Howard et al., 2022) calculated *via* Eq. 1:

$$\alpha =\frac{\frac{\left(N_{d-1}-N_{d-2}\right)}{\left(t_{d-1}-t_{d-2}\right)}+2\frac{N_{d-1}}{t_{d}-t_{d-1}}}{2\frac{N_{d}}{t_{d}-t_{d-1}}+\frac{N_{d}-N_{d+1}}{t_{d+1}-t_{d}}}$$ (1)

in which $N_{d}$ , $N_{d-i}$ , and $N_{d+i}$ are the total tumor cell counts at the discontinuity, i points before the discontinuity, and i points after the discontinuity, respectively. $t_{d}$ , $t_{d-i}$ , and $t_{d+i}$ are the times of the discontinuity, i points before the discontinuity, and i points after the discontinuity, respectively. The objective of this normalization was to smooth the first and second derivatives of the total tumor cell counts across the discontinuity, as proposed in (Howard et al., 2022). Supplementary Appendix A in the Supplementary Information provides further details about the purpose and derivation of Eq. 1.

#### Outlier removal

For datasets that possessed outliers, the *rmoutliers* function from MATLAB R2020b (The Mathworks, Natick, MA) was used to remove data points using median filtering. A visual inspection of the resulting data confirmed that this method removed evident outliers from the original series, while maintaining the natural fluctuations in tumor cell counts (see Supplementary Figures S1–S5).

### Mathematical model

**FIGURE 1:**
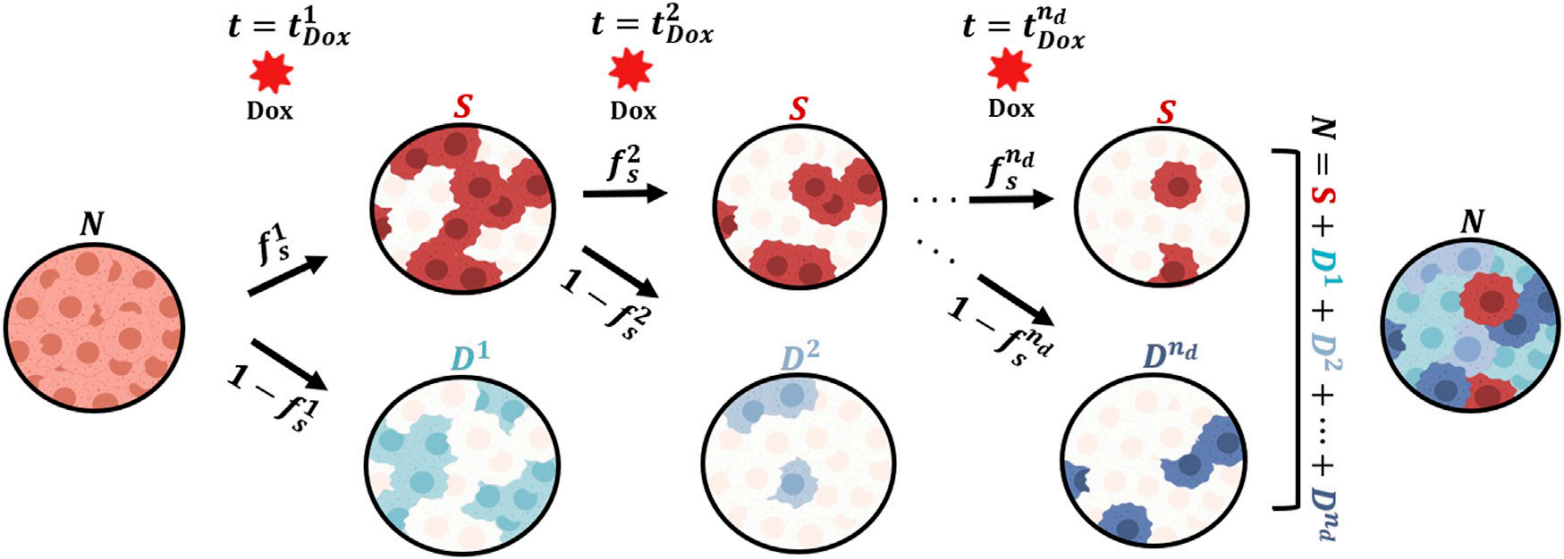
Generalized model of tumor cell response to multiple doses of doxorubicin treatment. We start with a population of untreated tumor cells and let them grow for approximately 48 h. At time $t_{Dox}^{1}$ , we add a dose of doxorubicin (Dox) to each well. We assume that after the treatment, the tumor cells either survive ( S ) or are irreversibly damaged ( $D^{1}$ ) and ultimately die due to the cytotoxic action of doxorubicin. The fraction of cells in either subpopulation is determined by $f_{s}^{1}$ **,** the fraction of surviving cells after the first dose. After the subsequent $n_{d}$ doses of doxorubicin ( $i=2,3,\ldots ,n_{d}$ ), we assume that a fraction $f_{s}^{i}$ of the surviving cells survive the treatment, while a fraction ( $1-f_{s}^{i}$ ) induces a new subpopulation of irreversibly damaged cells ( $D^{i})$ , such that the total number of tumor cells is $N=S+D^{1}+D^{2}+...{+D}^{n_{d}}$ for times $t>t_{Dox}^{n_{d}}$ . In this study, we further assess whether the collection of $f_{s}^{i}$ can be assumed to take on the same value or whether they require an independent parameterization with each doxorubicin dose. This Figure was created using BioRender.com.

We present a mathematical model to describe the response of MCF-7 breast cancer cells to the cytotoxic action of doxorubicin in the three experimental scenarios listed in Table 1. We begin by describing the biological mechanisms captured by the model assuming a single dose of doxorubicin (Experiment 1, Table 1). Then, we show how the model can be generalized to multiple doses (Experiments 2 and 3, Table 1), and can also be modified to vary specific parameters with each dose. Figure 1 illustrates the main tumor cell dynamics described by our model after each dose of doxorubicin, which are further detailed in the following paragraphs. The reader can refer to Supplementary Table S1 for a consolidated list of model parameter definitions and their units.

#### Single-dose model

We start with a population of tumor cells ( N ) that grow untreated for a specified period of time prior to doxorubicin treatment (approximately 48 h). We assume that these untreated cells follow logistic growth:

$$\frac{dN}{dt}=g_{0}N\left(1-\frac{N}{\theta_{u}}\right)$$ (2)

$$N\left(0\right)=N_{0}$$ (3)

where $g_{0}$ is the untreated proliferation rate, $\theta_{u}$ is the untreated tumor cell carrying capacity, and $N_{0}$ is the initial number of tumor cells. We set $\theta_{u}$ = 53,873 cells, which corresponds to the mean value resulting from the fitting of Eqs 2, 3 to the untreated datasets in Experiment 1 (i.e., 0 nM doxorubicin; further details can be found in Supplementary Tables S2–S5 and Supplementary Figure S1).

Let $t_{Dox}^{1}$ denote the time at which a single dose of doxorubicin is delivered, as described in Experiment 1 (Table 1). At this time point, we assume that a fraction $f_{s}$ of the tumor cells survives the treatment ( S) , whereas the complementary fraction $\left(1-f_{s}\right)$ is irreversibly damaged by the cytotoxic action of doxorubicin and will ultimately die (D) (Anampa et al., 2015; Howard et al., 2022; Gewirtz, 1999; Carvalho et al., 2009; McKenna et al., 2017). Hence, the fraction $f_{s}$ ultimately depends on the underlying spectrum of drug sensitivities in the tumor cell population as well as on the amount of drug delivered with each dose (Howard et al., 2022; Gewirtz, 1999; Carvalho et al., 2009; McKenna et al., 2017; Zoli et al., 1995). We denote the initial number of surviving and irreversibly damaged tumor cells immediately after treatment with doxorubicin as $S\left(t_{Dox}^{1+}\right)$ and $D\left(t_{Dox}^{1+}\right)$ , respectively, which are defined based on the number of untreated cells immediately before the delivery of doxorubicin, $N\left(t_{Dox}^{1-}\right)$ , as

$$S\left(t_{Dox}^{1+}\right)=f_{s}N\left(t_{Dox}^{1-}\right)$$ (4)

$$D\left(t_{Dox}^{1+}\right)=\left(1-f_{s}\right)N\left(t_{Dox}^{1-}\right)$$ (5)

such that the total tumor cell number N for time $t>t_{Dox}^{1}$ is calculated as

N(t)=S(t)+D(t) (6)

Note that Eqs 4–6 ensure the continuity in the tumor cell count before and after the treatment with doxorubicin, as observed in the corresponding experimental data (see Supplementary Figure S2).

We assume that the surviving cells also follow logistic growth with a different rate and carrying capacity:

$$\frac{dS}{dt}=g_{s}S\left(1-\frac{N}{\theta_{Dox}}\right)$$ (7)

where $g_{s}\;$ is the proliferation rate of surviving tumor cells and $\theta_{Dox}$ is the treated tumor cell carrying capacity. For the irreversibly damaged cells, we assume that their logistic growth dynamics gradually transition from proliferation to treatment-induced death at an exponentially-decaying rate:

$$\frac{dD}{dt}=\left(k_{d}+\left(g_{d}-k_{d}\right)\exp\left(-\gamma_{d}\left(t-t_{Dox}^{1}\right)\right)\right)D\left(1-\frac{N}{\theta_{Dox}}\right),$$ (8)

where $k_{d}$ and $g_{d}$ denote the drug-induced death rate and the proliferation rate of the irreversibly damaged tumor cells, respectively, while $\gamma_{d}$ represents the drug-induced death delay rate. The latter mechanism represents the varying duration of the cascade of biological events that takes place between drug exposure and the ultimate doxorubicin-induced tumor cell death (e.g., uptake by tumor cells, damage to DNA, cell cycle arrest, induction of tumor cell death) (Howard et al., 2022; Gewirtz, 1999; Carvalho et al., 2009; McKenna et al., 2017). In Eq. 8, the use of a common logistic model formulation to describe the initial growth after treatment and the ensuing drug-induced death in the irreversibly-damaged tumor cell compartment facilitates the modeling of this transition in the dynamics of this subpopulation within the growth rate of the logistic model (i.e., the first factor in parenthesis in the right-hand side of Eq. 8). Additionally, the cytotoxic action of doxorubicin targets proliferating cells (Howard et al., 2022; Gewirtz, 1999; Carvalho et al., 2009; McKenna et al., 2017). The logistic model formulation in Eq. 8 further enables to account for the limitation to tumor cell proliferation depending on the total tumor cell density, which may hence limit drug-induced tumor cell death.

In Eqs 7, 8, we introduce a treated tumor cell carrying capacity $\left(\theta_{Dox}\right)$ , which may be different from $\theta_{u}$ defined for untreated cells in Eq. 2. The rationale for this modeling choice is inspired by the experimental data from Howard et al. (2022), which shows that the maximum tumor cell counts in the replicates treated with doxorubicin could reach either larger or smaller values at confluence with respect to their untreated counterparts (i.e., the 0 nM replicates in Experiment 1; see Supplementary Figures S1–S5). To estimate $\theta_{Dox}$ in Eqs 7, 8, we used either one of two approaches. If the last tumor cell count in a dataset was greater than 30% of $\theta_{u}$ , then $\theta_{Dox}$ was fit along with the other model parameters. Conversely, if the last tumor cell count was less than 30% of $\theta_{u},$ then we fixed $\theta_{Dox}$ to the mean of the values obtained from the replicates of the same experiment in which this parameter was directly fit. The rationale for this approach is that we observed that final tumor cell counts below 30% of $\theta_{u}$ did not provide enough identifiability for $\theta_{Dox}$ , which ultimately induced significant model fitting errors.

#### Multiple-dose model

Let us now consider a treatment schedule consisting of $n_{d}$ doses of doxorubicin delivered at times $t_{Dox}^{i}$ , ( $i=1,2,\ldots ,n_{d}$ ), as described in Experiments 2 and 3 (Table 1). For the first dose, we assume that a fraction $f_{s}^{1}$ of the tumor cells survives treatment with doxorubicin and, thus, the multiple-dose model remains identical to the single-dose model described in the previous section. For each of the subsequent drug doses, we assume that a fraction $f_{s}^{i}$ ( $i=2,\ldots ,n_{d})$ of the tumor cells that escaped the cytotoxic action of the previous doxorubicin doses, $S\left(t_{Dox}^{i-}\right),$ survives the new dose, while a corresponding fraction $1-f_{s}^{i}$ gives rise to a new irreversibly damaged population $D^{i}$ . We further assume that each new subpopulation $D^{i}$ is characterized by a distinct value of the death delay rate $\gamma_{d}^{i}$ . The rationale for considering an adaptive parameterization of parameters $f_{s}$ and $\gamma_{d}$ with each doxorubicin dose is that the underlying spectrum of tumor cell sensitivities may significantly change with each doxorubicin dose and inter-treatment interval (Howard et al., 2022; Pisco et al., 2013; Álvarez-Arenas et al., 2019; Lyman, 2009; De Souza et al., 2011; Ponnusamy et al., 2017), such that the surviving fraction may exhibit an increased resistance to the drug. We hypothesize that this phenomenon results in a higher potential to survive a new drug dose (i.e., larger values of $f_{s}$ ) or to partially hinder the cytotoxic action of doxorubicin before ultimately succumbing (i.e., lower values of $\gamma_{d}$ ).

Thus, after the delivery of the $i^{th}$ dose ( i≥2) , the number of surviving tumor cells $S\left(t_{Dox}^{i+}\right)$ and the initial number of the new subpopulation of irreversibly damaged cells $D^{i}\left(t_{Dox}^{i+}\right)$ are calculated as

$$S\left(t_{Dox}^{i+}\right)=f_{s}^{i}S\left(t_{Dox}^{i-}\right)$$ (9)

$$D^{i}\left(t_{Dox}^{i+}\right)=\left(1-f_{s}^{i}\right)S\left(t_{Dox}^{i-}\right)$$ (10)

such that the total number of tumor cells during and after treatment with multiple doses of doxorubicin is given by

$$N\left(t\right)=S\left(t\right)+\overset{n_{d}}{\underset{i=1}{\sum }}D^{i}\left(t\right)H\left(t-t_{Dox}^{i}\right),$$ (11)

where $H\left(t-t_{Dox}^{i}\right)$ is the Heaviside step function, which equals 0 for t < $t_{Dox}^{i}$ and 1 for t ≥ $t_{Dox}^{i}$ . Note that Eqs 8–11 ensure the continuity in the total tumor cell number before and after each doxorubicin dose, as observed in the data from Experiments 2 and 3 (Table 1; see Supplementary Figures S3–S5).

In this multiple-dose model, we assume that the surviving cells continue to follow logistic growth after each of the consecutive doxorubicin doses, as described by Eq. 7. Additionally, each of the $i^{th}$ irreversibly damaged subpopulations are assumed to follow the growth dynamics defined in Eq. 8. Thus, for $i=1,\ldots ,n_{d}$ , the dynamics of each irreversibly damaged subpopulation $D^{i}$ is given by

$$\frac{dD^{i}}{dt}=\left(k_{d}+\left(g_{d}-k_{d}\right)\exp\left(-\gamma_{d}\left(t-t_{Dox}^{i}\right)\right)\right)D^{i}\left(1-\frac{N}{\theta_{Dox}}\right)$$ (12)

Finally, we further investigate two versions of the multiple-dose model: 1) the general formulation outlined above in which we vary $f_{s}$ and $\gamma_{d}$ with the delivery of each dose, and 2) a simplified version in which we assume a constant parameterization for all doses (i.e., $f_{s}^{1}=f_{s}^{2}=\ldots =f_{s}^{n_{d}}$ and $\gamma_{d}^{1}=\gamma_{d}^{2}=\ldots =\gamma_{d}^{n_{d}}$ ). Our underlying hypothesis is that longer inter-treatment intervals require an adaptive parameterization because they contribute to the development of chemoresistance (Howard et al., 2022; Lyman, 2009; De Souza et al., 2011; Ponnusamy et al., 2017), which would be represented in our model by higher fractions of surviving cells ( $f_{s}^{i}$ ) along with irreversibly damaged subpopulations ( $D^{i}$ ) exhibiting longer transition times from proliferation to treatment-induced death (i.e., lower values of $\gamma_{d}^{i}$ ). Conversely, we hypothesize that shorter inter-treatment intervals may not introduce significant changes to the survival fractions ( $f_{s}^{i}$ ) and death delay rates ( $\gamma_{d}^{i}$ ) associated with each drug dose, such that a constant parameterization would suffice to capture the tumor cell population response to the cytotoxic action of the prescribed doxorubicin treatment. In the Results section, we show that these hypotheses are significantly supported by the fitting of these two model versions to the data from Experiments 2 and 3 (Table 1).

### Numerical methods

#### Model fitting

We fit the single-dose model to each individual time course of total tumor cell counts from each replicate from Experiment 1 (*n* = 60; Table 1), and we fit the multiple-dose model to each individual time course of total tumor cell counts from each replicate from Experiments 2 and 3 (*n* = 108 and 120, respectively; Table 1). Model fitting was carried out with a nonlinear least-squares method, *via* the MATLAB (R2020b) function *lsqnonlin*. We leveraged a trust-region reflective algorithm with function, step, and optimality tolerances of 10^–6^, while the maximum number of function evaluations and iterations was set to 20,000. The parameter bounds and initial guesses were guided by the results from Howard et al. (2022), and are summarized in Supplementary Tables S2, S8, S12, S16, S17. The ordinary differential equations in our models were solved using a Runge-Kutta method as provided by *ode45* in MATLAB (R2020b). Supplementary Appendix B in the Supplementary Information provides further details about the model fitting approach used in this study.

#### Empirical parameter formulas

We constructed empirical formulas for the single-dose model parameters as a function of doxorubicin concentration based on the model fittings to the datasets from Experiment 1 (Table 1). To this end, we also applied a nonlinear least-squares method using a trust-region reflective algorithm provided by *lsqnonlin* in MATLAB (R2020b), as described in the previous section. The initial guess and bounds for the empirical parameters in these formulas were chosen according to the range of the single-dose model parameter values obtained from the fittings to the datasets from Experiment 1 (Table 1). The medians of the distributions of these fitted model parameters at each doxorubicin concentration were used as the observed values for the empirical parameter formula fits. The choice of the of the empirical formula for each parameter was based on the observed trend of the fitted parameter values obtained with the single-dose model as a function of increasing doxorubicin concentration (e.g., an exponentially decaying trend was represented with an exponential function; see the Results section and Supplementary Tables S6, S7 for further details).

### Statistical analysis

To assess our model’s quality of fit to the time course data, we calculated the coefficient of determination ( $R^{2}$ ), the normalized root mean squared error (NRMSE), the Pearson correlation coefficient (PCC), and the concordance correlation coefficient (CCC) (Lin, 1989). In the Results section, we report the median and range of these metrics across all the replicates of each experiment (i.e., *n* = 60, 108, 60, and 60 for Experiment 1, Experiment 2, Experiment 3 with 2-day inter-treatment interval, and Experiment 3 with 2-week inter-treatment interval, respectively). More detailed values can be found in Supplementary Tables S4, S10, S14, S20. We further assessed our model parameterizations and fits to experimental data through 95% nonlinear regression parameter confidence intervals and 95% nonlinear regression prediction confidence intervals calculated using *nlparci* and *nlpredci* in MATLAB (R2020b), respectively (see Supplementary Appendix C). To test for significant differences between two values of a model parameter or quality-of-fit metric within each experimental scenario, we performed two-sided Wilcoxon rank sum tests with 5% significance using *ranksum* in MATLAB (R2020b).

To assess the validity of the proposed empirical formulas using the single-dose model fittings, we ran a simulation test in which we qualitatively compared the model outcomes based on these formulas with the corresponding experimental observations at each drug concentration. To this end, Latin hypercube sampling based on *lhsdesign* in MATLAB (R2020b) was used to define 200 parameter combinations assuming uniform distributions over the 95% confidence intervals of the fitted empirical parameter formulas at each doxorubicin concentration, as calculated by *nlpredci* in MATLAB (R2020b).

## Results

### Fitting the single-dose model to experiment 1 data: Varying doxorubicin concentrations

**FIGURE 2:**
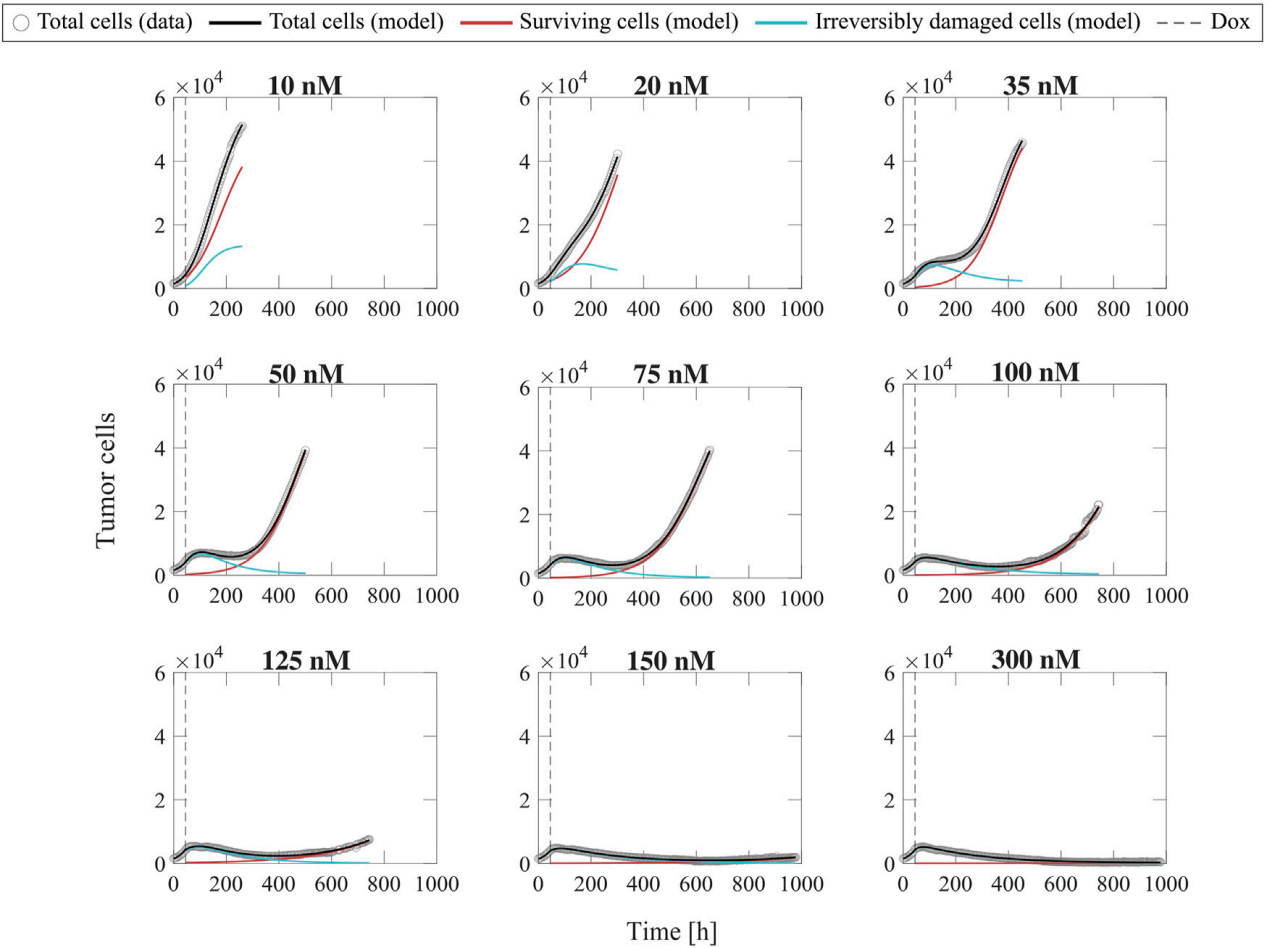
Representative fits of the single-dose model for varying concentrations of doxorubicin. Data and model fittings are shown for a representative replicate treated with 10–300 nM doxorubicin concentrations (Experiment 1, Table 1). Experimental data are shown in gray circles. The number of total cells, surviving cells, and irreversibly damaged cells obtained with the fitted single-dose model are shown in black, red, and blue solid lines, respectively. The time of doxorubicin delivery (Dox) is represented with a vertical grey dashed line. As doxorubicin concentration is increased, we observe a decrease in the growth rate of surviving cell subpopulation and a faster transition from growth to treatment-induced death in irreversibly damaged cell subpopulation. These drug-induced effects ultimately translate into a longer delay (or even suppression) of total tumor cell population growth post-treatment and lower total tumor cell count for higher doxorubicin concentrations, indicating superior tumor control overall. The median and range of the quality of fit metrics across all replicates in Experiment 1 (*n* = 60, Table 1) are NRMSE: 3.33 [0.80, 12.20], $R^{2}$ : >0.99 [0.96, >0.99], PCC: >0.99 [0.98, >0.99], and CCC: 0.99 [0.97, >0.99].

**FIGURE 3:**
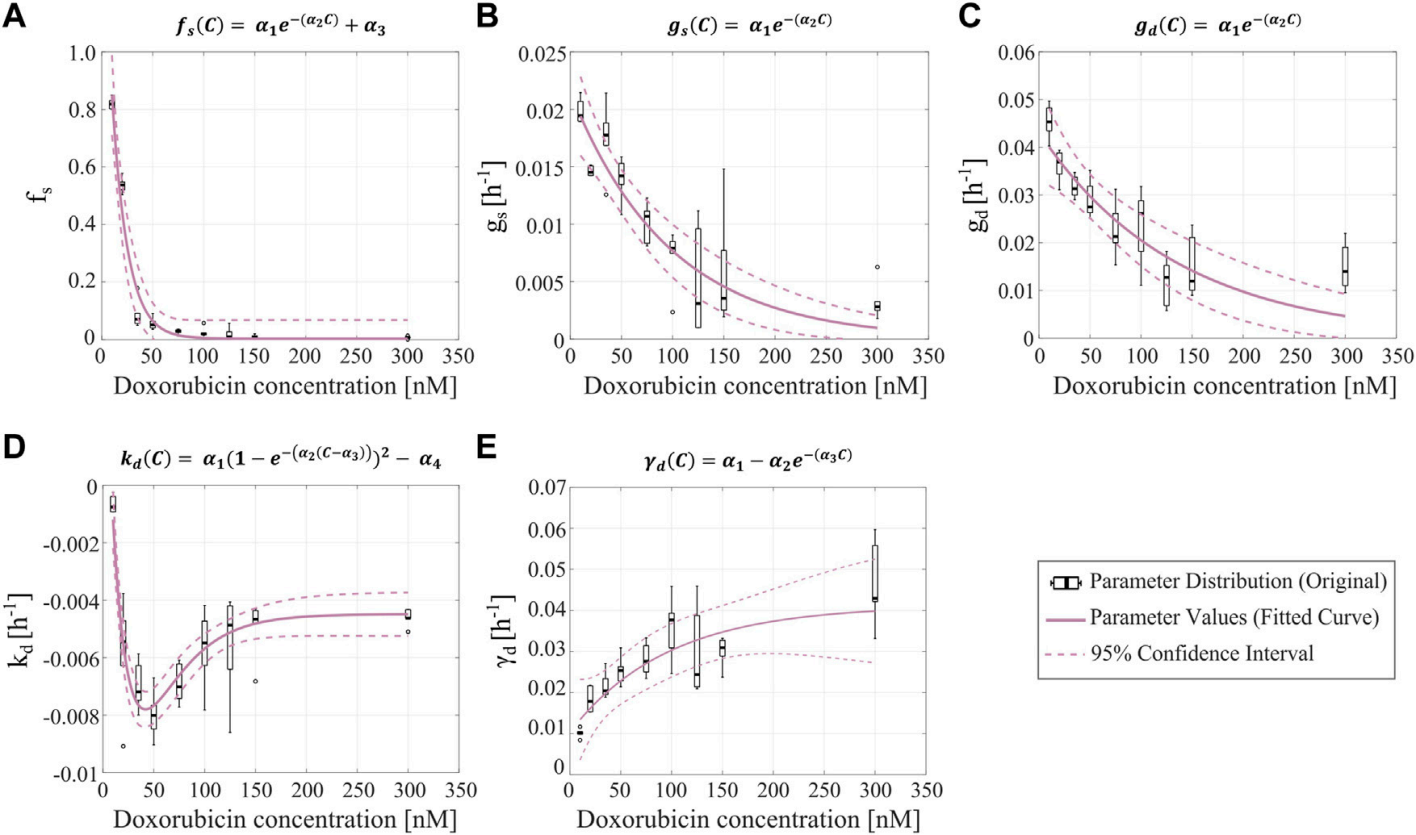
Empirical parameter formulas for varying doxorubicin concentrations. The proposed empirical formulas indicated at the top of each panel **(A–E)** were fit to the median of the corresponding parameter distributions obtained from fitting the single-dose model to the varying concentration datasets from Experiment 1 (Table 1). C denotes doxorubicin concentration in nM, while $\alpha_{i}$ ( i=1,2,… ) are empirical parameters. The distributions of the single-dose model parameters are represented with black boxplots, in which outliers are represented as black circles. The resulting curves from fitting the empirical parameter formulas are shown as purple solid lines, and their corresponding 95% confidence intervals are plotted as purple dashed lines. Panel **(A)** shows the parameter formula for the fraction of surviving cells ( $f_{s}$ ). Panel **(B)** shows the parameter formula for the proliferation rate of the surviving tumor cells ( $g_{s}$ ). Panel **(C)** shows the parameter formula for the proliferation rate of the irreversibly damaged tumor cells ( $g_{d}$ ). In panels **(A**–**C)**, we observe that as the drug concentration increases, the corresponding single-dose model parameter values decrease exponentially. Panel **(D)** shows the parameter formula for the doxorubicin-induced death rate of irreversibly damaged cells ( $k_{d}$ ), which we approximated with an equation based on a Morse-potential relationship. Panel **(E)** shows the parameter formula for the doxorubicin-induced death delay rate of irreversibly damaged cells ( $\gamma_{d}$ ), which increases and then plateaus as the drug concentration increases. Median and range of quality of fit metrics for the empirical parameter formulas (*n* = 5) are NRMSE: 17.65 [5.45, 153.6], $R^{2}$ : 0.91 [0.78, 0.98], PCC: 0.95 [0.88, 0.99], and CCC: 0.85 [0.78, 0.88].

Figure 2 shows representative model fits for the observed growth of MCF-7 cell populations treated with only one dose of doxorubicin at concentrations ranging from 10 to 300 nM (Experiment 1, Table 1). Model fits for all replicates at each drug concentration (*n* = 6) can be found in Supplementary Figure S2. We report the median and range of all the fitted model parameters for each doxorubicin concentration in Supplementary Table S3, while Figure 3 shows the boxplots of the fitted parameter distributions for each doxorubicin concentration. The median and range of the quality of fit metrics for the single-dose model fits to Experiment 1 data (*n* = 60) were: NRMSE (3.33 [0.80, 12.20]), $R^{2}$ (>0.99 [0.96, >0.99]), PCC (>0.99 [0.98, >0.99]), and CCC (0.99 [0.97, >0.99]). Supplementary Table S4 further provides detailed quality of fit metrics for each doxorubicin concentration. Figure 2, Supplementary Figure S2 and Supplementary Table S3 show that, as doxorubicin concentration is increased, the surviving cells exhibit a decrease in growth rate and number, while the irreversibly damaged cells undergo a faster transition from proliferation to treatment-induced death. These trends ultimately lead to significantly lower final total tumor cell counts ( p < 0.05, see Supplementary Table S5) and larger delay or even suppression of tumor regrowth in the cells exposed to higher doxorubicin concentrations (see Supplementary Figure S2), suggesting that tumor control improves as the doxorubicin dose is increased.

Figure 3 shows the fitted empirical formulas for the fraction of surviving cells ( $f_{s}$ )*,* the proliferation rate of the surviving cells ( $g_{s}$ ), the proliferation rate of the irreversibly damaged cells ( $g_{d}$ ), the doxorubicin-induced death rate of the irreversibly damaged cells ( $k_{d}$ ), and the doxorubicin-induced death delay rate of irreversibly damaged cells ( $\gamma_{d})$ . These empirical formulas are functions of doxorubicin concentration, which is denoted with C . The fitted empirical parameter values and their confidence intervals can be found in Supplementary Table S6, while the corresponding quality of fit metrics can be found in Supplementary Table S7. For $f_{s},$ $g_{s}$ , and $g_{d},$ we observe a clear exponentially decaying trend as drug concentration is increased (Figures 3A–C). In the case of $f_{s}$ , we added an additional constant empirical parameter to the decaying exponential to ensure that the empirical formula captures the low nonzero values of this parameter for the higher doxorubicin concentrations (otherwise, the exponential decay would reach the horizontal asymptote at $f_{s}$ = 0 for low doxorubicin concentrations). The parameter $k_{d}$ exhibits a complex trend, consisting of a steep decreasing branch for doxorubicin concentrations under 50 nM, followed by an increasing branch that plateaus for doxorubicin concentrations over 150 nM. We found that an empirical formula based on a Morse-potential relationship (Girifalco and Weizer, 1959) captured this trend (Figure 3D). For $\gamma_{d}$ , we chose a decaying exponential flipped with respect to the horizontal axis to capture the increasing trend that ultimately plateaus at a nonzero value (Figure 3E). The median and range of the quality of fit metrics for the five proposed empirical formulas were: NRMSE (17.65 [5.45, 153.6]), $R^{2}$ (0.91 [0.78, 0.98]), PCC (0.95 [0.88, 0.99]), and CCC (0.85 [0.78, 0.88]). We note that the NRMSE of the fitted empirical formula for $f_{s}$ reached values beyond 100%. This is due to the small values of $f_{s}$ at high concentrations of doxorubicin, where the NRMSE is not relevant to modeling outcomes; for reference, the RMSE for the fitted empirical formula for $f_{s}$ is 0.0476.

**FIGURE 4:**
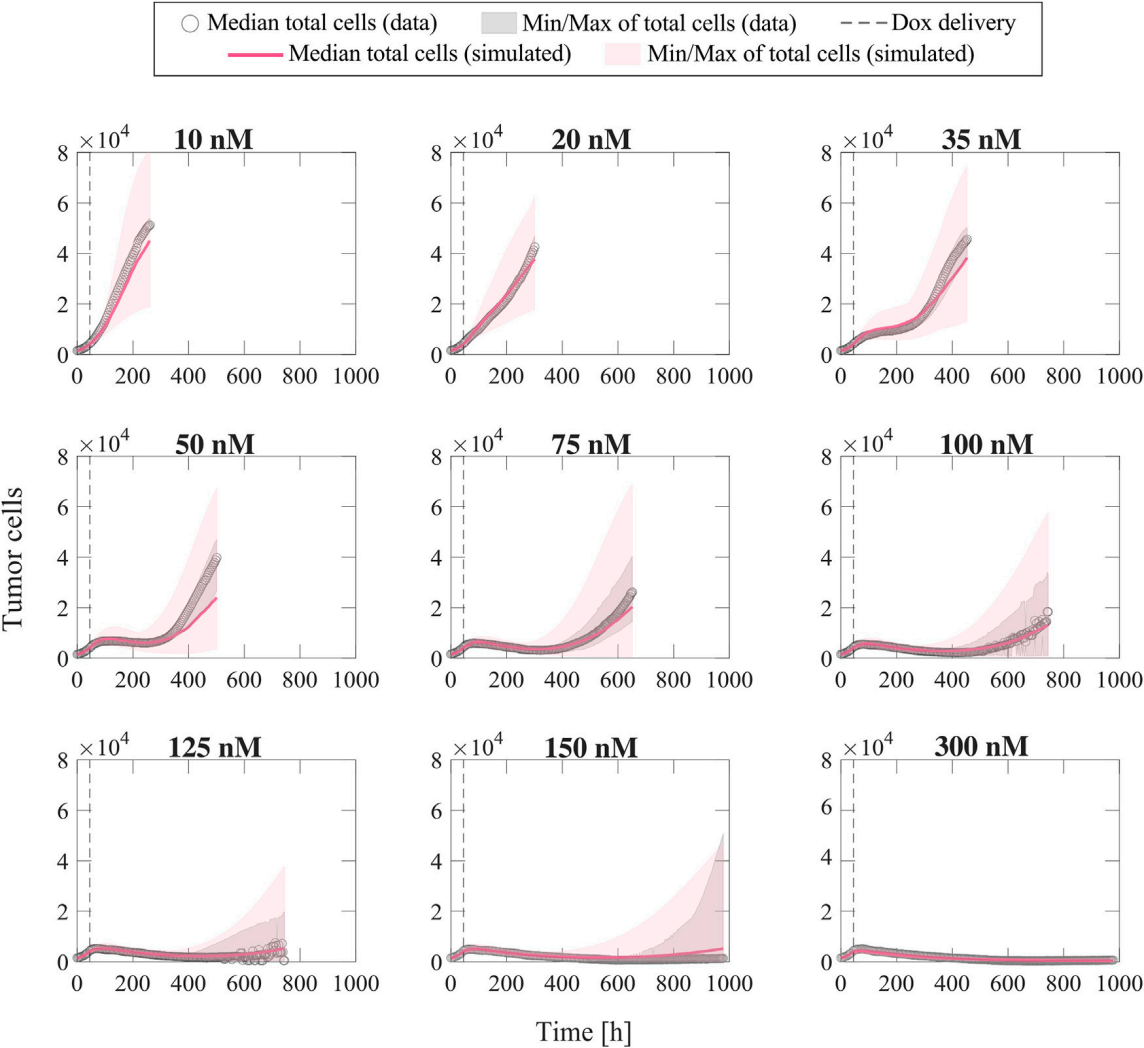
Comparison of simulated tumor cell population growth based on empirical parameter formulas with respect to experimental data for varying doxorubicin concentrations. We sampled the 95% confidence intervals for the fitted empirical parameter formulas in Figure 3 using Latin hypercube sampling to obtain 200 parameter combinations for each doxorubicin concentration, with which we carried out corresponding simulations with the single-dose model. The median and range of the model simulations are plotted with the median and range of the experimental data from Experiment 1 (Table 1) at each time point for comparison. The median of the experimental data is shown with gray circles, and the range of the experimental data is represented with gray shaded regions. The median of the model simulations is plotted as a pink solid line, and the range of the simulations is shown as pink shaded regions. The time of doxorubicin delivery is represented with a vertical grey dashed line. We observe that our fitted parameter formulas from Figure 3 can reproduce a wide range of tumor cell population dynamics, including those observed in the varying concentration datasets (Experiment 1, Table 1).

Once the parameter formulas had been established, we wanted to qualitatively assess the range of tumor cell population dynamics that our formulas could reproduce. For each doxorubicin concentration, we sampled the 95% confidence intervals of the fitted empirical parameter formulas (dashed purple lines in Figure 3) using Latin hypercube sampling to obtain 200 parameter combinations, with which we ran corresponding model simulations. Figure 4 presents the median and range of the model simulations plotted against the median and range of the experimental data measured at each time point for each doxorubicin concentration tested in Experiment 1 (Table 1). We observe that the proposed empirical parameter formulas (Figure 3) are able to predict a wide range of model solutions and that the simulations are able to capture the overall tumor cell population dynamics observed in the datasets from Experiment 1 (Table 1).

### Fitting the multiple-dose model to experiment 2 data: Varying inter-treatment intervals

**FIGURE 5:**
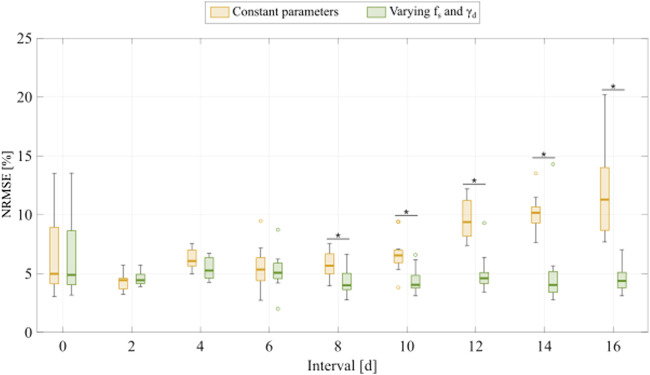
Comparison of fitting the experimental data for varying inter-treatment intervals with the multiple-dose model with constant versus varying parameters. For each inter-treatment interval tested in Experiment 2 (Table 1), we compared the normalized root mean squared error (NRMSE) calculated from the fittings using the multiple-dose model with constant parameters (yellow boxplots) with the NRMSE calculated from the fittings using the multiple-dose model with varying $f_{s}$ and $\gamma_{d}$ (green boxplots). Outliers are represented with circles. At inter-treatment intervals of 8, 10, 12, 14, and 16 days, there is a significantly lower NRMSE when the model with varying $f_{s}$ and $\gamma_{d}$ is used ( p : 0.0061, 0.0017, 1.56 $\times 10^{-4}$ , 5.92 $\times 10^{-4}$ , 3.66 $\times 10^{-5}$ , respectively). An asterisk (*) indicates p< 0.05 (two-sided Wilcoxon rank sum test).

To fit the experimental data for varying inter-treatment intervals (Experiment 2, Table 1), we initially used the two versions of the multiple-dose model; i.e., with all parameters held constant or varying $f_{s}$ and $\gamma_{d}$ with each drug dose. Figure 5 shows the distribution of the NRMSE in fitting the datasets at each inter-treatment interval (*n* = 12) for both models. We observe a significant difference between the NRMSEs obtained with either version of the multiple-dose model, such that the varying $f_{s}$ and $\gamma_{d}$ model provides a significantly lower NRMSE at 8-, 10-, 12-, 14-, and 16-day inter-treatment intervals ( p : 0.0061, 0.0017, 1.56 $\times 10^{-4}$ , 5.92 $\times 10^{-4}$ , 3.66 $\times 10^{-5}$ , respectively). Thus, the results shown in Figure 5 justify the use of the model with constant parameters for inter-treatment intervals shorter than 8 days and the model with varying $f_{s}$ and $\gamma_{d}$ for inter-treatment intervals ≥8 days. We followed this model selection criterion for fitting the datasets from Experiments 2 and 3 (Table 1) for the remainder of this work.

**FIGURE 6:**
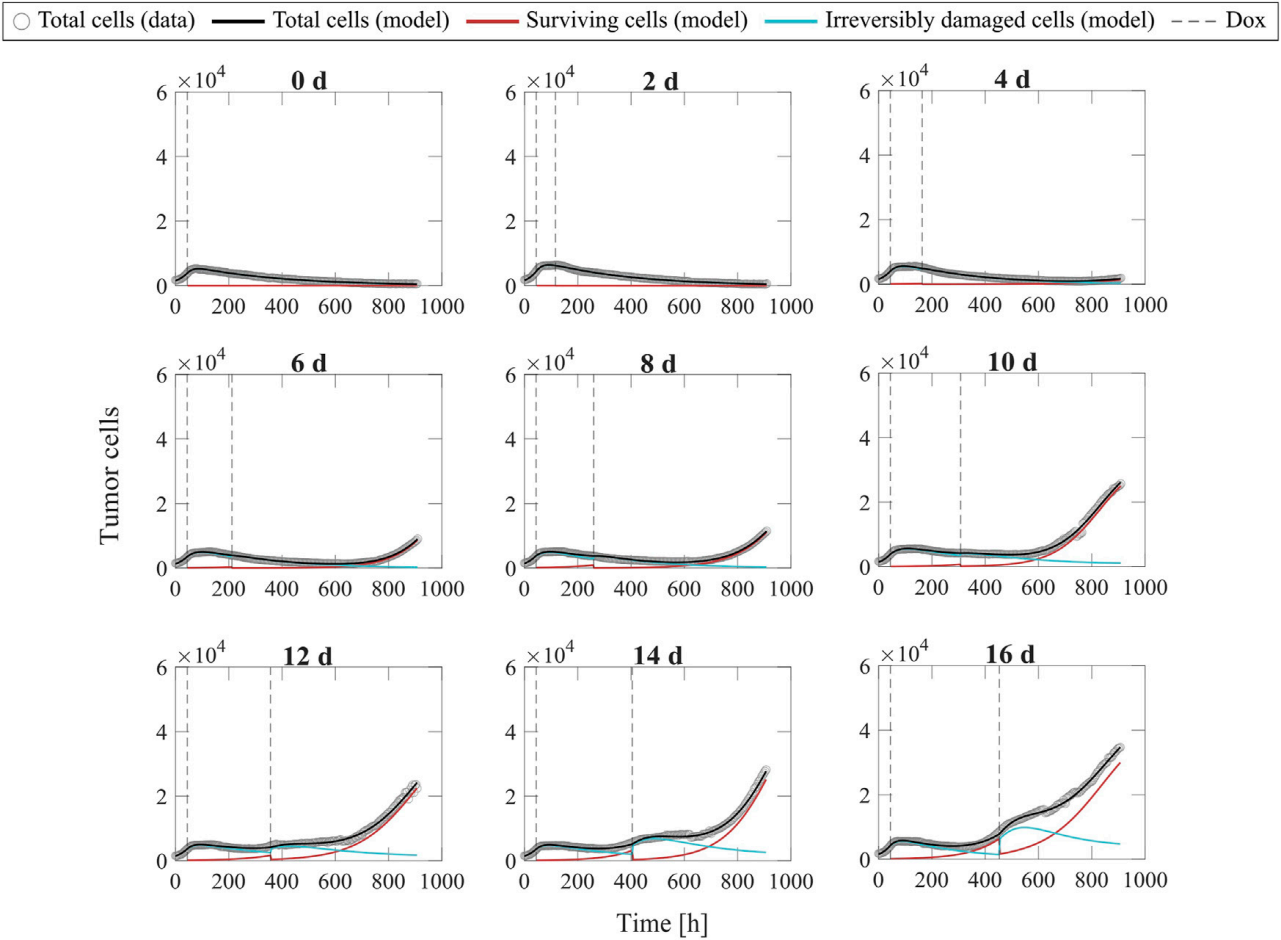
Representative fits of the multiple-dose model for varying inter-treatment intervals. Data and model fittings are shown for a representative replicate exposed to two doses of 75 nM doxorubicin delivered at inter-treatment intervals ranging from 0 to 16 days (Experiment 2, Table 1). Experimental data are shown in gray circles. The number of total cells, surviving cells, and irreversibly damaged cells obtained with the fitted multiple-dose model are shown in black, red, and blue solid lines, respectively. The times of doxorubicin (Dox) delivery are represented with vertical grey dashed lines. For the 0-day case, a single line represents a continuous treatment with no interval between the doses. For inter-treatment intervals of 0–6 days, the multiple-dose model with constant parameters was used for data fitting. For inter-treatment intervals of 8–16 days, we used the multiple-dose model with varying $f_{s}$ and $\gamma_{d}$ . As the inter-treatment interval is lengthened, we observe an increase in the proliferation rate of the surviving cells and a slower transition from proliferation to treatment-induced death in the irreversibly damaged cells. These drug-induced effects ultimately lead to a tumor cell population relapse after the second dose for inter-treatment intervals of 6 days or longer in most replicates, as well as tumor cell population regrowth after the first dose for inter-treatment intervals of 12 days or longer. These observations suggest increasingly poor tumor control as the two doses of 75 nM of doxorubicin are spaced further out in time. The median and range of the quality of fit metrics across all datasets in Experiment 2 (*n* = 108, Table 1) are NRMSE: 4.64 [2.74, 14.3], $R^{2}$ : 0.99 [0.80, >0.99], PCC: >0.99 [0.91, >0.99], and CCC: 0.99 [0.90, >0.99].

Figure 6 shows representative model fits for the observed growth of MCF-7 cell populations treated with two doses of 75 nM doxorubicin delivered at inter-treatment intervals ranging from 0 to 16 days (Experiment 2, Table 1). Model fits for all the replicates at each inter-treatment interval (*n* = 12) can be found in Supplementary Figure S3. Additionally, Supplementary Table S9 summarizes the median and range of the fitted model parameters for each inter-treatment interval. The median and range of the quality of fit metrics across all replicates in Experiment 2 (*n* = 108) were: NRMSE (4.64 [2.74, 14.3]), $R^{2}$ (0.99 [0.80, >0.99]), PCC (>0.99 [0.91, >0.99]), and CCC (0.99 [0.90, >0.99]). More detailed quality of fit metrics for each inter-treatment interval are reported in Supplementary Table S10. As the inter-treatment interval lengthens, the surviving cells tend to adopt an increasingly larger proliferation rate and the irreversibly damaged cells transition more slowly from proliferation to drug-induced death after two doses of doxorubicin treatment (see Figure 6, Supplementary Figure S3 and Supplementary Table S9). These effects appear to promote the tumor cell population regrowth after the second dose in most replicates for inter-treatment intervals of 6 days or longer and after the first dose for inter-treatment intervals of 12 days or longer. Overall, this ultimately leads to significantly higher final total tumor cell counts as the inter-treatment interval is lengthened ( p < 0.05, see Supplementary Table S11), suggesting that increased time spans between consecutive doses of doxorubicin is conducive to poorer tumor control.

**FIGURE 7:**
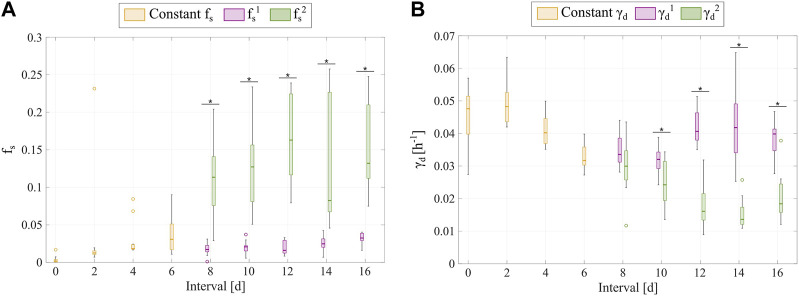
Comparison of the $f_{s}$ and $\gamma_{d}$ distributions obtained from fitting the multiple-dose model to the experimental data for varying inter-treatment intervals. The parameter distributions are represented as boxplots and were obtained from fitting the multiple-dose model to the varying inter-treatment interval datasets from Experiment 2 (Table 1). Outliers are represented with circles. Panel **(A)** shows the distributions for the fraction of surviving cells ( $f_{s}$ ). Panel **(B)** shows the distributions for the doxorubicin-induced death delay rate of the irreversibly damaged tumor cells ( $\gamma_{d}$ ). For 0–6 days inter-treatment intervals, $f_{s}$ and $\gamma_{d}$ are kept constant in the model (yellow boxplots); whereas, for 8–16 days inter-treatment intervals, we vary $f_{s}$ and $\gamma_{d}$ with each doxorubicin dose ( $f_{s}^{1},\gamma_{d}^{1}$ : purple boxplots, $f_{s}^{2},\gamma_{d}^{2}$ : green boxplots). As the inter-treatment interval is lengthened from 0 to 6 days, the constant $f_{s}$ and $\gamma_{d}$ show a trend towards higher surviving fractions and slower transitions to doxorubicin-induced death, suggesting increasingly poorer tumor control. When $f_{s}$ and $\gamma_{d}$ are varied with each dose, we observe that the second $f_{s}$ values correspond to significantly higher surviving fractions for 8-, 10-, 12-, 14-, and 16- day inter-treatment intervals ( $\text{p}:\;4.7\times 10^{-5},\;3.7\times 10^{-5}$ , $3.7\times 10^{-5}$ , $3.7\times 10^{-5}$ , and $3.7\times 10^{-5}$ , respectively) and that the second $\gamma_{d}$ values represent significantly slower transitions to treatment-induced death for 10-, 12-, 14-, and 16- day inter-treatment intervals ( p: 0.0141 , $3.7\times 10^{-5}$ , $6.0\times 10^{-5}$ , and $9.7\times 10^{-5},\;$ respectively). These changes in $f_{s}$ and $\gamma_{d}$ after the second dose also suggest an increasingly poorer tumor control after the second dose with a longer inter-treatment interval. An asterisk (*) indicates p< 0.05 in two-sided Wilcoxon rank sum tests comparing the distributions of the two $f_{s}$ and $\gamma_{d}$ values obtained for each inter-treatment interval where the multiple-dose model with varying parameters was used.

Additionally, Figure 7 shows the distributions of the fitted $f_{s}$ and $\gamma_{d}$ values from fitting the multiple-dose model to the data with varying inter-treatment intervals (Experiment 2, Table 1). When the model with constant parameters is used (inter-treatment intervals from 0 to 6 days), we observe a trend towards higher surviving fractions and delayed transitions to treatment-induced death in irreversibly damaged cells as the two doses are further spaced in time. This observation is further supported by the distributions of varying $f_{s}$ and $\gamma_{d}$ obtained from fitting the multiple-dose model to the data for inter-treatment intervals from 8 to 16 days. After the second dose in each of these longer intervals, the surviving fraction significantly increases and the transition from proliferation to treatment-induced death in irreversibly damaged cells significantly slows ( p < 0.05, see Figure 7), thereby suggesting an enhanced chemoresistance in both tumor cell subpopulations for longer inter-treatment intervals. Additionally, comparing the distributions of parameter $f_{s}^{2}$ obtained for the different inter-treatment intervals considered in Experiment 2, the values obtained in the 0-day and 2-day cases are significantly lower than those obtained in any larger intervals, the $f_{s}^{2}$ values obtained in the 4-day and 6-day scenarios are significantly lower than those obtained for any inter-treatment interval larger or equal to 8 days, and the $f_{s}^{2}$ values obtained in the 8-day case are significantly lower than those obtained for the 12-day inter-treatment interval (*p* < 0.05; see Supplementary Appendix D for further detail). Likewise, for parameter $\gamma_{d}^{2}$ , the values obtained for the 0-day, 2-day, 4-day, 6-day, and 8-day cases are significantly larger than those obtained for any inter-treatment interval larger or equal to 6, 4, 6, 10, and 12 days, respectively (*p* < 0.05; see Supplementary Appendix D). Furthermore, the $\gamma_{d}^{2}$ values obtained for the 10-day inter-treatment interval are significantly larger than those obtained for the 12-day and 14-day intervals, and the $\gamma_{d}^{2}$ values obtained for the 14-day inter-treatment interval are significantly lower than those obtained for the 16-day interval (*p* < 0.05; see Supplementary Appendix D). Thus, these results along with the distributions plotted in Figure 7 show that there is a tendency towards a larger $f_{s}^{2}$ value for larger inter-treatment intervals, which is suggestive of increased chemoresistance in the surviving cell compartment. However, this trend becomes less clear among the longest intervals considered in Experiment 2 (i.e., >8 days), for which $f_{s}^{2}$ appears to plateau at a value between 0.10 and 0.15. Additionally, our results also show a decreasing trend in the values of $\gamma_{d}^{2}$ between the 0-day and the 14-day inter-treatment interval scenario, which suggests an increase in the chemoresistance of the irreversibly-damaged cells (i.e., they transition more slowly from proliferation to drug-induced cell death); although this tendency is reverted for the 16-day inter-treatment interval. Moreover, the distributions shown in Figure 7 further support the use of the multiple-dose model with varying $f_{s}$ and $\gamma_{d}$ for longer inter-treatment intervals.

### Fitting the multiple-dose model to experiment 3 data: Varying number of doses

**FIGURE 8:**
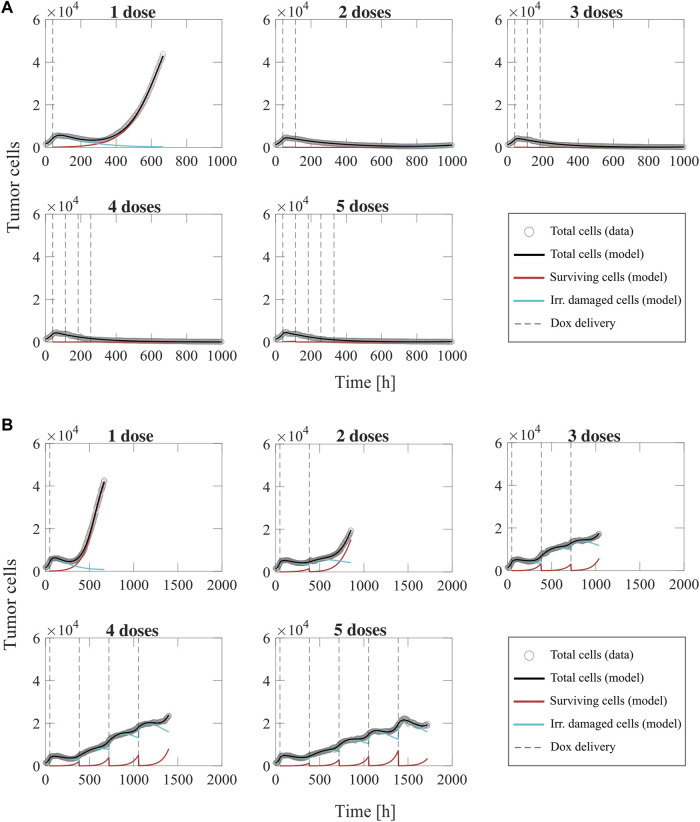
Representative fits of the multiple-dose model for a varying number of doxorubicin doses. Data and model results are shown for a representative replicate treated with 1–5 doses of 75 nM doxorubicin delivered at either 2-day or 2-week inter-treatment intervals (Experiment 3, Table 1). Experimental data are shown in gray circles. The number of total cells, surviving cells, and irreversibly damaged cells obtained with the fitted multiple-dose model are shown in black, red, and blue solid lines, respectively. The times at which doxorubicin (Dox) is delivered are represented with vertical grey dashed lines. Panel **(A)** shows fittings for 1 to 5 doxorubicin doses delivered at 2-day inter-treatment intervals obtained with the model with constant parameters. The median and range of the quality of fit metrics across all replicates for this Experiment 3 subgroup (*n* = 60, Table 1) are NRMSE: 12.2 [2.72, 19.1], $R^{2}$ : 0.99 [0.87, >0.99], PCC: 0.99 [0.93, >0.99], CCC: 0.99 [0.93, >0.99]. Panel **(B)** shows fittings for 1 to 5 doxorubicin doses delivered at 2-week inter-treatment intervals obtained with the model with varying $f_{s}$ and $\gamma_{d}$ . The median and range of the quality of fit metrics across all replicates for this Experiment 3 subgroup (*n* = 60, Table 1) are NRMSE: 3.21 [1.91, 8.57], $R^{2}$ : >0.99 [0.93, >0.99], PCC: >0.99 [0.97, >0.99], CCC: 0.99 [0.96, >0.99]. Overall, we observe that there is superior tumor control with an increased number of doses, which is further improved when the doses are delivered at shorter inter-treatment intervals. As the inter-treatment interval is lengthened from 2 days to 2 weeks, we observe that the population growth rate and number of the surviving cells increase, while the irreversibly damaged cells exhibit a slower transition from proliferation to treatment-induced death.

Figure 8 shows representative model fits for the observed growth of MCF-7 cell populations treated with 1–5 doses of 75 nM doxorubicin delivered at either 2-day or 2-week inter-treatment intervals (Experiment 3, Table 1). The datasets from the cells treated with a 2-day inter-treatment interval were fitted with the multiple-dose model with constant parameters, while the datasets from the cells treated with a 2-week inter-treatment interval were fitted with the multiple-dose model with varying $f_{r}$ and $\gamma_{d}$ . Model fits for all the replicates for each number of doses and both inter-treatment intervals (*n* = 12) can be found in Supplementary Figures S4, S5. The median and range of the fitted model parameters for each dose number are summarized in Supplementary Tables S13, S18, S19. For the replicates treated every 2 days, the median and range of the quality of fit metrics (*n* = 60) were: NRMSE (12.2 [2.72, 19.1]), $R^{2}$ (0.99 [0.87, >0.99]), PCC (0.99 [0.93, >0.99]), and CCC (0.99 [0.93, >0.99]). Likewise, for the replicates treated every 2 weeks, the median and range of the quality of fit metrics (*n* = 60) were: NRMSE (3.21 [1.91, 8.57]), $R^{2}$ (>0.99 [0.93, >0.99]), PCC (>0.99 [0.97, >0.99]), CCC (0.99 [0.96, >0.99]). More detailed quality of fit metrics for each number of doses and both inter-treatment intervals can be found in Supplementary Tables S14, S20.

The model fittings plotted in Figure 8 and Supplementary Figures S4, S5 show that increasing the number of doses contributed to improved tumor control for the two inter-treatment intervals investigated in this work. In general, for the cells treated every 2 days, we observed significantly lower final total tumor cell counts as the number of doses was increased ( p < 0.05, see Supplementary Table S15). Furthermore, delivering two or more doses effectively suppressed tumor growth at the end of the experiment, typically showing a decreasing branch in the total tumor cell count right after the first dose. When the inter-treatment interval was extended to 2 weeks, delivering more than one dose of doxorubicin also contributed to limited tumor cell growth ( p < 0.05, see Supplementary Table S21); however, most of the replicates showed an increasing trend in total tumor cell count over the experiment duration. Thus, with a 2-week inter-treatment interval, an increasing number of doses can decelerate tumor cell growth, but it cannot suppress it as observed with a 2-day inter-treatment interval. Furthermore, the model fitting results reported in Figure 8, Supplementary Figures S4, S5 and Supplementary Tables S3, S18, S19 show that, as the inter-treatment interval is lengthened from 2 days to 2 weeks, the surviving cells exhibit a larger proliferation rate, while the irreversibly damaged cells emerging after the second and subsequent doses undergo a slower transition to treatment-induced death. These effects, induced by the lengthened inter-treatment interval, contribute to explaining the superior tumor control in the 2-day experiments and align with the corresponding results shown in Figure 6, Supplementary Figure S3 and Supplementary Table S9.

**FIGURE 9:**
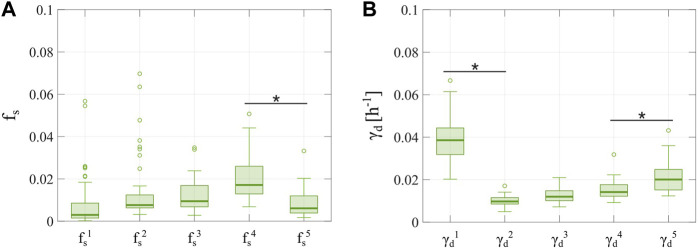
Distributions of $f_{s}$ and $\gamma_{d}$ obtained from fitting the multiple-dose model to the experimental data for a varying number of doses with a 2-week inter-treatment interval. The parameter distributions are represented as boxplots and were obtained from fitting the multiple-dose model to the 2-week inter-treatment interval datasets from Experiment 3 (Table 1), in which the model with varying $f_{s}$ and $\gamma_{d}$ was used. Outliers are represented as circles. Panel **(A)** shows the distributions of the fraction of surviving cells, such that a new value for $f_{s}$ is defined for each drug dose ( $f_{s}^{1},f_{s}^{2},\ldots ,f_{s}^{5}$ ). We observe an increasing trend in the first four $f_{s}$ parameters, which suggests an increasing chemoresistance with each dose. However, $f_{s}^{5}$ takes on significantly lower values than $f_{s}^{4}$ ( p=0.0015 ), which suggests that adding more doses may limit the trend towards chemoresistance. Panel **(B)** shows the distributions of the doxorubicin-induced death delay rate of irreversibly damaged cells, such that a new value of $\gamma_{d}$ is defined for each drug dose ( $\gamma_{d}^{1}$ , $\gamma_{d}^{2}$ ,…, $\gamma_{d}^{5}$ ). The values for $\gamma_{d}^{2}$ are significantly lower than those of $\gamma_{d}^{1}$ ( $p=5.6\times 10^{-19}$ ). Hence, the second irreversibly damaged subpopulation shows a slower transition to treatment-induced death. However, the subsequent doxorubicin doses induce irreversibly damaged subpopulations exhibiting an increasing $\gamma_{d}$ , with $\gamma_{d}^{5}$ being significantly larger than $\gamma_{d}^{4}\;\left(p=0.0093\right).$ This observation further suggests that past a certain number of doses, initial chemoresistance appears to be reverted. An asterisk (*) indicates p< 0.05 (two-sided Wilcoxon rank sum test).

We further investigated tumor cell dynamics for the Experiment 3 data with 2-week inter-treatment intervals by analyzing the evolving distributions of parameters $f_{s}$ and $\gamma_{d}$ , which are shown in Figure 9. We observe that the surviving fraction corresponding to the first to fourth doses ( $f_{s}^{1},f_{s}^{2},f_{s}^{3},f_{s}^{4}$ ) shows an increasing trend, which is indicative of progressive chemoresistance during treatment and aligns with the corresponding results shown in Figure 7. However, the fitted values for $f_{s}^{5}$ are significantly lower than the value obtained for $f_{s}^{4}$ ( p = 0.0015) . Additionally, we observe that the values for $\gamma_{d}^{2}$ are significantly lower than that of $\gamma_{d}^{1}$ ( p = $5.6\times 10^{-19}$ ), following the trend observed in Figure 7 for the data from Experiment 2. However, the values for $\gamma_{d}^{2}$ , $\gamma_{d}^{3}$ , $\gamma_{d}^{4},$ and $\gamma_{d}^{5}$ exhibit an increasing trend, with $\gamma_{d}^{5}$ being significantly larger than $\gamma_{d}^{4}\;\left(p=0.0093\right)$ . These changes in $f_{s}$ and $\gamma_{d}$ suggest that delivering multiple doses of doxorubicin may progressively limit or even revert the chemoresistance observed in the initial surviving and irreversibly damaged subpopulations.

## Discussion

We have presented a mathematical framework to describe the therapeutic response of MCF-7 breast cancer cells to treatment with doxorubicin and the development of chemoresistance in the *in vitro* setting. Our mathematical models rely on a compartmentalization of the tumor cell population after the delivery of each drug dose into either surviving cells or irreversibly damaged cells, the latter of which ultimately die due to the cytotoxic action of doxorubicin (Anampa et al., 2015; Howard et al., 2022; Gewirtz, 1999; Carvalho et al., 2009; McKenna et al., 2017). With this dichotomy, we aim to capture the underlying diverse spectrum of drug sensitivities in the tumor cell population, as well as its changes after the delivery of subsequent doxorubicin doses considering different inter-treatment interval lengths (Howard et al., 2022; Pisco et al., 2013; Álvarez-Arenas et al., 2019). We presented a single-dose model that can be extended to a multiple-dose model, in which parameterization can vary with each dose. We fitted our models to various time-resolved microscopy datasets, which enabled us to evaluate tumor cell population dynamics with our models in three experimental scenarios that varied either the doxorubicin concentration, the inter-treatment interval, or the number of doses (see Table 1). In all three cases, our models recapitulated the experimental observations, achieving a remarkable quality of fit.

In Experiment 1 (Table 1), we evaluated the effect of a single dose of doxorubicin on MCF-7 breast cancer cell population growth and we found that tumor control was significantly improved with increased drug concentration ( p < 0.05, see Supplementary Table S5). Our single-dose model showed that, at a subpopulation level, these dynamics emerged from a lower proliferation rate of surviving cells and a faster transition from proliferation to treatment-induced death in irreversibly damaged cells. The dynamics observed in our varying concentration experiment have also been reported in other studies of doxorubicin effects on breast cancer cell lines, both as monotherapy and in combination with other therapeutic agents (McKenna et al., 2017; Zoli et al., 1995; Czeczuga-Semeniuk et al., 2004).

We used the parameter distributions obtained from our single-dose model fits to the varying drug concentration datasets to empirically fit various parameter formulas as functions of doxorubicin concentration, as shown in Figure 3. The model simulations generated from our proposed empirical parameter formulas were able to capture a spectrum of model solutions that encompass the dynamics observed in our data from Experiment 1 (see Figure 4). We observed clear exponentially decaying trends for the fraction of surviving cells ( $f_{s})$ and the proliferation rates of surviving and irreversibly damaged tumor cells ( $g_{s}$ and $g_{d}$ , respectively) as doxorubicin concentration increases (see Figures 3A–C). These trends seem to capture the growth-inhibition effect of doxorubicin as well as the dose-response curve for this drug within our mechanistic modeling framework, in which doxorubicin efficacy has been observed to plateau at high concentrations (El-Kareh and Secomb, 2005; Neale et al., 2000). The distributions of the doxorubicin-induced death rate in the irreversibly damaged cells ( $k_{d})$ exhibited a non-monotonic trend as doxorubicin concentration was varied, which we approximated with a Morse-potential relationship (Girifalco and Weizer, 1959). This result was counterintuitive, as we had initially anticipated a strictly decreasing trend in $k_{d}$ for higher doxorubicin doses, which would indicate an increasingly more intense effect of treatment-induced death. However, the cytotoxic action of doxorubicin (Gewirtz, 1999; Carvalho et al., 2009; McKenna et al., 2017) also induces cell cycle arrest. The interplay between these two drug-induced effects may ultimately lead to nonlinear tumor cell responses, such as the one captured by the empirical formula for $k_{d}$ proposed in this work. The relative participation of cell death and cell cycle arrest in the overall doxorubicin action on breast cancer cells may follow more complex dynamics that are not fully captured by our models, and thus requires further investigation. Indeed, to refine the description of these doxorubicin effects, our models could also be extended to account for the dynamics of doxorubicin uptake and binding (McKenna et al., 2017); although this would require additional data reporting on those phenomena. From a modeling point of view, previous studies have leveraged other formulations to represent cytotoxic drug action (e.g., an exponential decay, alternative transition terms from proliferation to drug-induced death) (Lorenzo et al., 2022; Howard et al., 2022; Colli et al., 2021) which could be explored with our modeling framework in a model selection study (Lorenzo et al., 2022) to assess the optimal approach to capture the cytotoxic action of doxorubicin.

Experiment 2 involved the delivery of two doses of doxorubicin to each replicate of MCF-7 breast cancer cells at varying-inter-treatment intervals ranging from 0 to 16 days (Table 1). We fit two versions of our multiple-dose model to these datasets: either with constant parameters or with $f_{s}$ and $\gamma_{d}$ varied at each drug dose. The model with constant parameters sufficed to describe the observed tumor cell population dynamics for inter-treatment intervals from 0 to 6 days, while the model with varying $f_{s}$ and $\gamma_{d}$ was superior for inter-treatment intervals from 8 to 16 days ( p < 0.05, see Figure 5). For two consecutive doses of doxorubicin delivered at varying inter-treatment intervals, our results showed significantly poorer tumor control with longer inter-treatment intervals ( p < 0.05, see Supplementary Table S11). In the fittings from the model with varying $f_{s}$ and $\gamma_{d}$ , we observed that the second dose induced a significantly larger $f_{s}^{2}$ and a lower $\gamma_{d}^{2}$ compared to the corresponding values of $f_{s}^{1}$ and $\gamma_{d}^{1}$ ( p < 0.05, see Figure 7), further supporting the adoption of an adaptive model parameterization for inter-treatment intervals from 8 to 16 days. Additionally, comparing the distributions of $f_{s}^{2}$ and $\gamma_{d}^{2}$ obtained for the different inter-treatment intervals, we observed a tendency towards higher survival fraction and a slower death delay rate for higher inter-treatment intervals. Nevertheless, $f_{s}^{2}$ appears to plateau in long inter-treatment intervals and the general trend observed for $\gamma_{d}^{2}$ is reverted in the 16-day interval scenario. Thus, the changes observed in $f_{s}$ and $\gamma_{d}$ in Experiment 2 suggest that longer inter-treatment intervals contribute to the development of chemoresistance in both tumor cell subpopulations in our model; although our results also suggest to further investigate whether the trends in $f_{s}^{2}$ and $\gamma_{d}^{2}$ are reverted for inter-treatment intervals larger than 16 days. From a biological perspective, long inter-treatment intervals may allow cancer cells to acquire chemoresistance through processes like treatment-induced mutations, altered epigenetics, and phenotype switching, which ultimately limit the efficacy of the second dose and may lead to tumor regrowth (Easwaran et al., 2014; Zhao, 2016; Dong et al., 2018; Ji et al., 2019; Meacham and Morrison, 2013; Echeverria et al., 2019; Kumar et al., 2019). This phenomenon has been observed in preclinical studies (Lyman, 2009; De Souza et al., 2011; Ponnusamy et al., 2017), but the trends are less clear in the clinical setting (Lyman, 2009; Richards et al., 1992; Citron et al., 2003; Untch et al., 2009; Foukakis et al., 2016).

In Experiment 3, we treated MCF-7 breast cancer cells with multiple doses of doxorubicin at either 2-day or 2-week inter-treatment intervals (Table 1). We observed significantly improved tumor control with an increased number of doses delivered at a 2-day inter-treatment interval ( p < 0.05, see Supplementary Table S15), with tumor cell population growth effectively suppressed after two or more doxorubicin doses. When the treatment interval was extended to 2 weeks, tumor cell population growth was significantly decelerated ( p < 0.05, see Supplementary Table S21) but not suppressed, aligning with our previous conclusions that longer inter-treatment intervals may promote chemoresistance. Moreover, these results underscore that, in comparison to the total number of doses, it is the treatment interval that holds a critical impact on determining overall tumor control. Indeed, as most patients receive chemotherapy treatments delivered every 1–3 weeks, our results point to the clinical importance of optimizing treatment interval in designing effective drug regimens (Jarrett et al., 2020a; Lyman, 2009; Richards et al., 1992; Citron et al., 2003; Untch et al., 2009; Foukakis et al., 2016). Additionally, the evolving distributions for the varying $f_{s}$ and $\gamma_{d}$ from the model fits to the 2-week inter-treatment interval datasets (see Figure 9) exhibit trends that potentially explain the relationship between the number of doses and the resulting chemoresistance dynamics. For $f_{s}$ , the initially increasing trend for the first four doses ( $f_{s}^{1},f_{s}^{2},f_{s}^{3},f_{s}^{4}$ ), suggests a progressive increase in chemoresistance with each dose. However, the values of $f_{s}^{5}$ were significantly lower than those of $f_{s}^{4}$ ( p = 0.0015 ), potentially indicating that increasing the number of doses may ultimately hinder chemoresistance. This is further corroborated by the trends for $\gamma_{d}$ , in which $\gamma_{d}^{2}$ drops significantly with respect to $\gamma_{d}^{1}$ ( p = $5.6\times 10^{-19}$ ), but $\gamma_{d}^{2}$ , $\gamma_{d}^{3}$ , $\gamma_{d}^{4},$ and $\gamma_{d}^{5}$ exhibit an increasing trend. This result suggests that further doses of doxorubicin can promote increasingly faster transitions to treatment-induced death, thus reverting the initial chemoresistance observed in the irreversibly damaged subpopulation. We do note that because we have only tested up to five doses of doxorubicin, further studies with a larger number of doses would be needed to further probe these trends.

Although our work presents promising insights into the mechanisms of chemoresistance, this study does have its limitations. First, we used a limited number of replicates within the scenarios explored in each experiment (*n* = 6 or 12, see Table 1). Since we do not observe uniform tumor cell populations dynamics across all replicates within each scenario, we would like to re-assess the observations in this study over a larger experimental setup, for example involving a higher number of replicates exposed to more diverse combinations of drug concentration, inter-treatment interval, and number of drug doses. This would enable us to investigate whether these observations are from doxorubicin effects altering tumor cell dynamics or whether the experimental conditions influence the development of a representative distribution of drug-resistant and drug-sensitive cells (e.g., ∼2,000 seeded cells/well might potentially limit the emergence of a resistant subpopulation, which may skew the observed response to treatment). Second, we also acknowledge the general limitations of extrapolating from *in vitro* systems to tumors in patients (Katt et al., 2016), as cell lines do not capture the unique, heterogeneous nature of each patient’s tumor. To address this limitation, we plan to evaluate our models on clinically-relevant breast cancer cells other than MCF-7 cells (ER + breast cancer), such as the BT-474 (ER + HER2+ breast cancer) and MD-MBA-231 (triple-negative breast cancer) considered by Howard et al. (2022). Third, our cells were grown in monolayers, which are not representative of the three-dimensional tumor geometry *in vivo*. However, our mathematical models could be made readily applicable to tumor cell spheroid data. In particular, our models could be extended to a set of partial differential equations, accounting for tumor cell mobility and spatially-resolved parameters and variables, which would allow for a spatiotemporal description of spheroid growth in both *in vitro* and *in vivo* settings (Jarrett et al., 2020a; Kazerouni et al., 2020). Indeed, these extended models could incorporate other spatially-varying mechanisms beyond tumor cell dynamics, such as drug diffusion, mechanics, and angiogenesis, which have also been recognized as key components of chemoresistance and drug action (Lankelma et al., 1999; Mascheroni et al., 2017; Yonucu et al., 2017; Jarrett et al., 2020b; Kazerouni et al., 2020). Fourth, given that our model requires a moderate number of parameters that may increase with the number of delivered doses of doxorubicin, their estimation from specific experimental data may exhibit a certain degree of uncertainty (see Supplementary Appendix C). Thus, future studies should investigate whether and how the levels of uncertainty obtained for the parameters of our models affect the description of the therapeutic action of doxorubicin on tumor cells, for example, by leveraging a robust Bayesian framework (Lorenzo et al., 2022). Fifth, we only analyzed a constant versus an adaptive parameterization for the surviving fraction ( $f_{s}$ ) and the death delay rate ( $\gamma_{d}$ ) because we hypothesized that these would suffice to account for the development of chemoresistance. While this choice was supported by the results presented herein, an uncertainty quantification approach could also be exploited to conduct a model selection study aiming to investigate the optimal combination of constant and adaptive parameters in Experiments 2 and 3 (Lorenzo et al., 2022), which may provide new insights in the development of chemoresistance to doxorubicin. Furthermore, while experimental observations and modeling results in our study support the adoption of a fixed value of $\theta_{Dox}$ after treatment, the aforementioned modeling selection analysis could also be extended to investigate whether the change in the carrying capacity after treatment (i.e., from $\theta_{u}$ to $\theta_{Dox}$ ) is permanent or temporal; although this analysis most likely requires additional experiments and data types to investigate the biological mechanisms underlying either of these two modeling alternatives (e.g., doxorubicin-induced changes in cell size or genetic alterations). Finally, we acknowledge the limitations in modeling tumor cell subpopulation dynamics with total tumor cell data, and that our study thus lacks methods for specifically validating the proposed mathematical description of surviving and irreversibly damaged tumor cell dynamics. This issue could potentially be addressed by incorporating methods to trace cell lineage, which would enable the collection of time-resolved measurements of the therapeutic response of diverse drug-sensitive and drug-resistant phenotypes in the tumor cell population. For instance, Al’Khafaji et al. (2018) have developed a functionalized lineage tracing tool to track both cell lineages and direct lineage-specific gene expression using barcoded gRNAs. Then, fitting these data to an extension of our model to a multicompartment formulation describing the dynamics of the various detected drug sensitivity phenotypes could provide a more precise insight into the dose-dependent response (including refined parameter empirical formulas) and how timing and the number of doses mediate the global response of the tumor cell population.

In future studies, we intend to explore a refinement of our model to account for the mechanisms underlying the trends observed in the empirical parameter formulas from this work, which will help us further understand doxorubicin effects. Additionally, we plan to extend the construction of these empirical formulas over the three-dimensional space spanned by the dosage, the inter-treatment interval, and the number of doses by leveraging a larger collection of replicate datasets exhibiting variations across those three treatment regimen variables. This experimental campaign would expand Experiments 2 and 3 in our study beyond a fixed drug concentration of 75 nM per dose, and an inter-treatment interval of 2 days or 2 weeks in Experiment 3 (see Table 1). We hypothesize that nonlinear dependencies will govern the relationship between the three regimen variables and the model parameters, thereby enabling the capture of a diverse spectrum of therapeutic responses to doxorubicin that we already observed in Experiments 2 and 3 (see Supplementary Figures S3–S5). Hence, the resulting three-dimensional empirical formulas could allow for predicting the outcome of any doxorubicin treatment regimen *a priori* (i.e., before running the corresponding experiment) by just selecting the treatment schedule (i.e., dosage, inter-treatment interval, and number of doses), but this would require previous validation by leveraging a different collection of experimental datasets than the one used to construct the three-dimensional empirical formulas. Further experimentally informed studies with our mechanistic models could also contribute to identifying the optimal timing and frequency for doxorubicin delivery in preclinical scenarios. Indeed, we would like to explore optimal control theory (Jarrett et al., 2020b; Colli et al., 2021) *in vitro* through heterogeneous multiclonal cultures to identify optimal treatment combinations of doxorubicin concentration, treatment interval, and number of doses.

High-throughput, time-resolved microscopy *in vitro* systems enable the collection of vast amounts of time-resolved data on the dynamics of tumor cell populations across multiple, diverse scenarios. We (and others) (Zhang et al., 2015; Strobl et al., 2021; Howard et al., 2022; Pisco et al., 2013; Álvarez-Arenas et al., 2019; McKenna et al., 2017; Kazerouni et al., 2020) posit that these time-resolved datasets can be integrated in mathematical models of tumor cell population dynamics to systematically investigate the effects of drugs on tumor cells across a much broader variety of regimens than are possible to test *in vivo*. Then, our ultimate goal is to exploit the knowledge gained from a model constructed and validated in a data-rich *in vitro* preclinical environment (e.g., where hundreds of data points are available for each replicate) to refine mathematical models and their predictions of therapeutic response in a data-poor *in vivo* clinical environment (i.e., where less than five data points may be available for each patient) (Lorenzo et al., 2022; Weis et al., 2015; Jarrett et al., 2018; Jarrett et al., 2020a). In particular, we think that the mechanistic insights provided by the models and empirical formulas proposed in this study could be leveraged to identify the minimal dose range required to effectively inhibit breast cancer growth *in vivo* and achieve optimal tumor control, both of which are of great clinical interest (Carvalho et al., 2009; Jarrett et al., 2020b; Harahap et al., 2020; Chan et al., 1999). Thus, we believe that the complex dynamics underlying the dose-dependent effect of doxorubicin deserve further research coupling extensive experiments with mechanistic modeling.

## Conclusion

We have developed a biologically-based, mathematical model of MCF-7 breast cancer cell response to the cytotoxic action of doxorubicin accounting for the development of chemoresistance, which significantly extends the experimentally-informed mechanistic models by Howard et al. (2022). To this end, we proposed a modeling framework that can accommodate multiple doxorubicin doses as well as an adaptive parameterization with each drug dose. We show that model fittings to longitudinal, time-resolved microscopy data of MCF-7 breast cancer cells could remarkably recapitulate the observed tumor cell population dynamics for all experimental scenarios varying in either drug concentration, inter-treatment interval, or number of doses. We also propose empirical formulas that describe model parameters as functions of doxorubicin concentration, which could contribute to refining our mechanistic model and further our understanding of doxorubicin action. We report significantly improved tumor control with higher doxorubicin concentrations, shorter inter-treatment intervals, and a higher number of doses. We also observe that longer inter-treatment intervals potentially promote chemoresistance, which manifests as higher surviving fractions and delayed transitions to treatment-induced death in irreversibly damaged subpopulations. Our findings show promise in furthering our understanding of doxorubicin action and chemoresistance progression, while also representing a step towards systematically exploring optimal treatment regimens *in vitro*.

## Data Availability

Publicly available datasets were analyzed in this study. This data can be found here: https://doi.org/10.5281/zenodo.6973776.
