# Supplementary material for: Mathematical characterization of population dynamics in breast cancer cells treated with doxorubicin
Source: Front Mol Biosci. 2022 Sep 12;9:972146. doi: 10.3389/fmolb.2022.972146 (PMC9510895; doi:10.3389/fmolb.2022.972146)
Supplement: Supplementary file 1 [file DataSheet1.PDF]

Supplementary Information

Mathematical characterization of population dynamics in breast cancer cells treated with doxorubicin

Emily Y. Yang[1], Grant R. Howard[2], Amy Brock[2-4], Thomas E. Yankeelov[1-3,5-7], Guillermo Lorenzo[1,8,*]

[1]Oden Institute for Computational Engineering and Sciences, The University of Texas at Austin, Austin, TX, USA

[2]Department of Biomedical Engineering, The University of Texas at Austin, Austin, TX, USA

[3]Livestrong Cancer Institutes, Dell Medical School, The University of Texas at Austin, Austin, TX, USA

[4]Interdisciplinary Life Sciences Program, The University of Texas at Austin, Austin, TX, USA

[5]Department of Diagnostic Medicine, The University of Texas at Austin, Austin, Austin, TX, USA

[6]Department of Oncology, The University of Texas at Austin, Austin, Austin, TX, USA

[7]Department of Imaging Physics, The University of Texas MD Anderson Cancer Center, Houston, TX, USA

[8]Department of Civil Engineering and Architecture, University of Pavia, Pavia, Italy

*Corresponding author

Oden Institute for Computational Engineering and Sciences

The University of Texas at Austin

201 E 24th St, 78712-1229 Austin TX, USA

Email: guillermo.lorenzo@utexas.edu, guillermo.lorenzo@unipv.it

Abbreviations

NRMSE: Normalized root mean square error

R^2: Coefficient of determination

PCC: Pearson correlation coefficient

CCC: Concordance correlation coefficient

Table of Contents

Supplementary Tables 1

Mathematical Model 1

S1. Definition and units of the model parameters 1

Experiment 1: Varying doxorubicin concentration 2

S2. Parameter initial guess and bounds 2

S3. Median and range of fitted model parameters 3

S4. Quality of fit metrics 4

S5. P-value matrix of final tumor cell number comparisons for Experiment 1 5

Experiment 1: Empirical parameter formulas 6

S6. Fitted empirical parameter values and confidence intervals 6

S7. Quality of fit metrics 6

Experiment 2: Varying treatment interval 7

S8. Parameter initial guess and bounds 7

S9. Median and range of fitted model parameters 8

S10. Quality of fit metrics 9

S11. P-value matrix of final tumor cell number comparisons for Experiment 2 10

Experiment 3: Varying number of doses 11

2-day inter-treatment interval 11

S12. Parameter initial guess and bounds 11

S13. Median and range of fitted model parameters 12

S14. Quality of fit metrics 13

S15. P-value matrix of final tumor cell number comparisons for Experiment 3 (2-day interval) 13

2-week inter-treatment interval 14

S16. Parameter initial guess and bounds: constant parameters 14

S17. Parameter initial guess and bounds: f_r and γ_d 14

S18. Median and range of fitted model parameters: constant parameters 15

S19. Median and range of fitted model parameters: f_r and γ_d 15

S20. Quality of fit metrics 16

S21. P-value matrix of final tumor cell number comparisons for Experiment 3 (2-week interval).... 16

Supplementary Figures 17

S1. Logistic model fits to the untreated datasets from Experiment 1 17

S2. Single-dose model fits to all the varying concentration datasets 18

S3. Multiple-dose model fits to all the varying inter-treatment interval datasets 21

S4. Multiple-dose model fits to all the datasets with varying number of doses: 2-day inter-treatment intervals 26

S5. Multiple-dose model fits to all the datasets with varying number of doses: 2-week inter-treatment intervals 29

Appendix A. Data normalization 32

Appendix B. Model fitting 34

Appendix C. Confidence intervals for model fits and parameters 35

Appendix D. Analysis of f_S^2 and γ_d^2 distributions in Experiment 2 42

Supplementary Tables

Parameter	Definition	Units
$\boldsymbol{N_0}$	Initial number of tumor cells	cells
$\boldsymbol{g_0}$	Baseline proliferation rate	h^{-1}
$\boldsymbol{\theta_u}$	Carrying capacity of untreated tumor cell population	cells
$\boldsymbol{\theta_{Dox}}$	Carrying capacity of treated tumor cell population	cells
$\boldsymbol{f_s}$	Fraction of surviving cells	-
$\boldsymbol{g_s}$	Proliferation rate of surviving tumor cells	h^{-1}
$\boldsymbol{g_d}$	Proliferation rate of irreversibly damaged tumor cells	h^{-1}
$\boldsymbol{k_d}$	Doxorubicin-induced death rate of irreversibly damaged tumor cells	h^{-1}
$\boldsymbol{\gamma_d}$	Doxorubicin-induced death delay rate of irreversibly damaged tumor cells	h^{-1}

Supplementary Table S1. Definition and units of the model parameters.

Dose [nM]	Initial guess and bounds							
	N_0 **[cells]**	g_0 **[h^{-1}]**	θ_{Dox} **[cells]**	f_s **[-]**	g_s **[h^{-1}]**	g_d **[h^{-1}]**	k_d **[h^{-1}]**	γ_d **[h^{-1}]**
**0**	$N_{0,obs}$ [700, 2000]	0.02 [0.0075, 0.035]	52100 [40000, 70000]*	-	-	-	-	-
**10**	$N_{0,obs}$ [1200, 3000]	0.03 [0.02, 0.035]	60000 [15000, 90000]	0.75 [0, 1]	0.02 [0.001, 0.05]	0.05 [0.001, 0.065]	-0.001 [-0.01, 0]	0.01 [1/120, 1/10]
**20**	〃	〃	〃	〃	〃	〃	〃	〃
**35**	〃	〃	50000 [15000, 90000]	0.5 [0, 1]	〃	〃	〃	0.015 [1/120, 1/10]
**50**	〃	〃	60000 [15000, 90000]	〃	〃	〃	〃	〃
**75**	〃	〃	〃	〃	〃	〃	〃	0.02 [1/120, 1/10]
**100**	〃	〃	〃	〃	〃	〃	〃	0.035 [1/120, 1/10]
**125**	〃	〃	〃	〃	〃	0.01 [0.001, 0.065]	〃	0.04 [1/120, 1/10]
**150**	〃	〃	80000 [15000, 90000]	0.25 [0, 1]	〃	〃	〃	0.05 [1/120, 1/10]
**300**	〃	〃	60000 [15000, 90000]	〃	〃	0.005 [0.001, 0.065]	-0.0025 [-0.01, 0]	〃

Supplementary Table S2. Parameter initial guesses and bounds used for fitting the single-dose model to time courses resulting from cells treated with a single dose of doxorubicin at varying concentrations (n = 6 per tested concentration; Experiment 1 in Table 1 of the main text). A ditto mark (〃) indicates that the initial guess and bounds are identical to the values in the cells above. *For 0 nM, we report the initial guess and bounds for θ_u instead of θ_{Dox}.

Dose [nM]	Parameter values							
	N_0 [cells]	g_0 [h^{-1}]	θ_{Dox} [cells]	f_s [-]	g_s [h^{-1}]	g_d [h^{-1}]	k_d [h^{-1}]	γ_d [h^{-1}]
**0**	1298 [1139, 1401]	0.028 [0.027, 0.028]	54439 [51372, 55575]*	-	-	-	-	-
**10**	1400 [1346, 1482]	0.026 [0.026, 0.027]	63579 [61403, 66880]	0.82 [0.80, 0.85]	0.019 [0.019, 0.021]	0.045 [0.04, 0.05]	-7.6×10^{-4} [-9.3×10^{-4}, -2.4×10^{-4}]	0.01 [0.0084, 0.012]
**20**	1444 [1283, 1475]	0.026 [0.025, 0.027]	77484 [71701, 83936]	0.54 [0.50, 0.58]	0.015 [0.014, 0.015]	0.037 [0.031, 0.039]	-0.0054 [-0.0091, -0.0038]	0.018 [0.015, 0.022]
**35**	1346 [1248, 1386]	0.024 [0.023, 0.024]	63576 [48622, 73338]	0.071 [0.049, 0.18]	0.018 [0.013, 0.021]	0.031 [0.029, 0.035]	-0.0072 [-0.008, -0.0059]	0.02 [0.019, 0.027]
**50**	1370 [1305, 1499]	0.024 [0.023, 0.025]	68827 [61904, 71593]	0.051 [0.036, 0.091]	0.014 [0.011, 0.021]	0.027 [0.025, 0.035]	-0.008 [-0.009, -0.0067]	0.025 [0.021, 0.031]
**75**	1469 [1326, 1563]	0.024 [0.022, 0.025]	68569 [52398, 77716]	0.031 [0.012, 0.036]	0.011 [0.0081, 0.016]	0.021 [0.015, 0.031]	-0.007 [-0.0077, -0.0061]	0.028 [0.023, 0.033]
**100**	1480 [1323, 1564]	0.023 [0.022, 0.025]	68167 [66466, 79285]	0.02 [0.015, 0.057]	0.0079 [0.0024, 0.0091]	0.026 [0.011, 0.032]	-0.0055 [-0.0078, -0.0042]	0.038 [0.025, 0.046]
**125**	1435 [1352, 1505]	0.026 [0.024, 0.028]	68167 [20746, 68167]	0.0095 [9×10^{-5}, 0.057]	0.0031 [0.001, 0.011]	0.013 [0.0058, 0.018]	-0.0049 [-0.0086, -0.0041]	0.024 [0.021, 0.046]
**150**	1444 [1234, 1562]	0.024 [0.022, 0.025]	68167 [68167, 88076]	0.0092 [4×10^{-5}, 0.02]	0.0035 [0.002, 0.015]	0.012 [0.009, 0.024]	-0.0047 [-0.0068, -0.0043]	0.031 [0.024, 0.033]
**300**	1482 [1381, 1546]	0.025 [0.024, 0.025]	68167 [68167, 68167]	0.0072 [5×10^{-4}, 0.014]	0.0028 [0.0018, 0.0063]	0.014 [0.0096, 0.022]	-0.0046 [-0.0051, -0.0043]	0.043 [0.033, 0.06]

Supplementary Table S3. Median and range of the single-dose model parameters obtained from the fits to the time courses resulting from cells treated with a single dose of doxorubicin at varying concentrations (n = 6 per tested concentration; Experiment 1 in Table 1 of the main text). *For 0 nM, we report the mean and range for θ_u instead of θ_{Dox}.

Dose [nM]	Quality of fit metrics			
	**NRMSE [%]**	R^2	**PCC**	**CCC**
**0**	1.12 [0.86, 1.50]	>0.999 [>0.999, >0.999]	>0.999 [>0.999, >0.999]	0.986 [0.986, 0.986]
**10**	1.13 [0.804, 1.2]	>0.999 [>0.999, >0.999]	>0.99 [>0.999, >0.999]	0.984 [0.984, 0.985]
**20**	1.46 [1.23, 2.29]	0.999 [0.999, >0.999]	>0.99 [0.999, >0.999]	0.986 [0.986, 0.986]
**35**	2.58 [0.804, 2.87]	0.999 [0.999, 0.999]	0.999 [0.999, >0.999]	0.991 [0.990, 0.991]
**50**	3.07 [0.804, 4.37]	0.998 [0.998, >0.999]	0.999 [0.999, >0.999]	0.991 [0.991, 0.992]
**75**	2.75 [0.804, 4.61]	0.999 [0.995, >0.999]	0.999 [0.997, >0.999]	0.994 [0.992, 0.994]
**100**	5.19 [0.804, 10.2]	0.993 [0.959, 0.997]	0.996 [0.980, 0.998]	0.991 [0.973, 0.993]
**125**	4.53 [0.804, 8.48]	0.996 [0.972, 0.997]	0.998 [0.986, 0.999]	0.993 [0.981, 0.994]
**150**	8.48 [0.804, 12.2]	0.990 [0.985, 0.999]	0.995 [0.993, >0.999]	0.991 [0.989, 0.996]
**300**	9.06 [0.804, 12]	0.990 [0.981, 0.996]	0.995 [0.991, 0.998]	0.992 [0.987, 0.994]

Supplementary Table S4. Median and range of the quality of fit metrics for the single-dose model fits to the time courses resulting from cells treated with a single dose of doxorubicin at varying concentrations (n = 6 per tested concentration; Experiment 1 in Table 1 of the main text).

Dose [nM]	**0**	**10**	**20**	**35**	**50**	**75**	**100**	**125**	**150**	**300**
**0**	-	0.9372	**0.0022**	**0.0087**	**0.0022**	**0.0022**	**0.0022**	**0.0022**	**0.0043**	**0.0022**
**10**	-	-	**0.0022**	**0.0022**	**0.0022**	**0.0022**	**0.0022**	**0.0022**	**0.0022**	**0.0022**
**20**	-	-	-	0.1797	0.0931	**0.0022**	**0.0022**	**0.0022**	0.0649	**0.0022**
**35**	-	-	-	-	0.0931	**0.0043**	**0.0022**	**0.0022**	0.0649	**0.0022**
**50**	-	-	-	-	-	0.0649	**0.0043**	**0.0022**	0.0931	**0.0022**
**75**	-	-	-	-	-	-	0.1797	**0.0152**	0.1797	**0.0022**
**100**	-	-	-	-	-	-	-	0.0931	0.3939	**0.0022**
**125**	-	-	-	-	-	-	-	-	0.3939	0.9372
**150**	-	-	-	-	-	-	-	-	-	0.2403
**300**	-	-	-	-	-	-	-	-	-	-

Supplementary Table S5. P-values from two-sided Wilcoxon rank sum tests comparing the observed final tumor cell numbers for every distinct combination of the varying doxorubicin concentration datasets (n = 6 per tested concentration; Experiment 1 in Table 1 of the main text). Values bolded in red indicate $p < 0.05$.

Parameter formula	Parameter values			
	α_1	α_2	α_3	α_4
$f_s(C) = \alpha_1 e^{-(\alpha_2 C)} + \alpha_3$	1.63<br>[1.05, 2.20]	0.065<br>[0.038, 0.093]	0.0032<br>[-0.061, 0.068]	-
$g_s(C) = \alpha_1 e^{-(\alpha_2 C)}$	0.022<br>[0.017, 0.026]	0.010<br>[0.0061, 0.015]	-	-
$g_d(C) = \alpha_1 e^{-(\alpha_2 C)}$	0.043<br>[0.033, 0.053]	0.0074<br>[0.0036, 0.011]	-	-
$k_d(C) = \alpha_1(1 - e^{-(\alpha_2(C-\alpha_3))})^2 - \alpha_4$	0.0033<br>[0.0023, 0.0044]	0.028<br>[0.02, 0.035]	41.92<br>[35.24, 48.60]	0.0078<br>[0.0072, 0.0084]
$\gamma_d(C) = \alpha_1 - \alpha_2 e^{-(\alpha_3 C)}$	0.035<br>[0.027, 0.043]	0.026<br>[0.006, 0.047]	0.0104<br>[-0.0062, 0.0270]	-

Supplementary Table S6. Fitted empirical parameter values and the corresponding 95% confidence intervals for the empirical parameter formulas derived from the single-dose model fits to the varying doxorubicin concentration datasets (n = 6 per tested concentration; Experiment 1 in Table 1 of the main text).

Parameter formula	Quality of fit metrics			
	**NRMSE [%]**	R^2	**PCC**	**CCC**
$f_s(C) = \alpha_1 e^{-(\alpha_2 C)} + \alpha_3$	153.64*	0.977	0.985	0.876
$g_s(C) = \alpha_1 e^{-(\alpha_2 C)}$	16.73	0.909	0.953	0.845
$g_d(C) = \alpha_1 e^{-(\alpha_2 C)}$	17.65	0.818	0.912	0.810
$k_d(C) = \alpha_1(1 - e^{-(\alpha_2(C-\alpha_3))})^2 - \alpha_4$	5.45	0.978	0.992	0.879
$\gamma_d(C) = \alpha_1 - \alpha_2 e^{-(\alpha_3 C)}$	17.94	0.784	0.883	0.779

Supplementary Table S7. Quality of fit metrics for the empirical parameter formulas derived from the single-dose model fits to the varying doxorubicin concentration datasets (n = 6 per tested concentration; Experiment 1 in Table 1 of the main text). *For larger drug concentrations, f_s takes on values on the order of 10^{-5}, such that natural variations of f_s across replicates may result in NRMSE values greater than 100%. However, these variations have a negligible impact on model outcome.

Interval [d]	Initial guess and bounds									
	N_0 [cells]	g_0 [h^{-1}]	θ_{Dox} [cells]	f_s^1 [-]	f_s^2 [-]	g_s [h^{-1}]	g_d [h^{-1}]	k_d [h^{-1}]	γ_d^1 [h^{-1}]	γ_d^2 [h^{-1}]
**Multiple-dose model with constant parameters**										
**0**	$N_{0,obs}$ [1000, 2500]	0.0234 [0.02, 0.035]	60000 [30000, 90000]	0.0337 [0, 1]		0.0101 [0.001, 0.05]	0.0277 [0.001, 0.05]	-0.0069 [-0.01, 0]	0.0325 [0, 1/15]	
**2**	〃	〃	〃	〃		〃	〃	〃	〃	
**4**	〃	〃	〃	〃		〃	〃	〃	〃	
**6**	〃	〃	〃	〃		〃	〃	〃	〃	
**Multiple-dose model with varying f_s and γ_d**										
**8**	$N_{0,obs}$ [1200, 2500]	0.0234 [0.02, 0.035]	60000 [15000, 90000]	0.0337 [0, 0.1]	0.005 [0, 1]	0.0101 [0.001, 0.05]	0.0277 [0.001, 0.055]	-5×10^{-4} [-0.01, 0]	0.0325 [0, 1/15]	1/75 [0, 1/15]
**10**	〃	〃	〃	〃	〃	〃	〃	〃	〃	〃
**12**	〃	〃	〃	〃	〃	〃	〃	〃	〃	〃
**14**	〃	〃	〃	〃	〃	〃	〃	〃	〃	〃
**16**	〃	〃	〃	〃	〃	〃	〃	〃	〃	〃

Supplementary Table S8. Parameter initial guesses and bounds used for fitting the multiple-dose model to the time courses resulting from cells treated with two consecutive doses of 75 nM doxorubicin delivered at varying inter-treatment intervals (n = 12 per tested interval; Experiment 2 in Table 1 of the main text). A ditto mark (〃) indicates that the initial guess and bounds are identical to the values in the cells above.

Interval [d]	Parameter values									
	N_0 [cells]	g_0 [h^{-1}]	θ_{Dox} [cells]	f_s^1 [-]	f_s^2 [-]	g_s [h^{-1}]	g_d [h^{-1}]	k_d [h^{-1}]	γ_d^1 [h^{-1}]	γ_d^2 [h^{-1}]
**Multiple-dose model with constant parameters**										
**0**	1468 [1354, 1542]	0.024 [0.023, 0.025]	53376 [42605, 80016]	0.0019 [7×10^{-5}, 0.017]		0.002 [0.001, 0.014]	0.022 [0.012, 0.026]	-0.0035 [-0.0047, -0.002]	0.046 [0.027, 0.057]	
**2**	1468 [1381, 1591]	0.024 [0.024, 0.025]	53376 [53376, 53376]	0.012 [0.0069, 0.23]		0.0045 [0.0028, 0.0069]	0.026 [0.023, 0.038]	-0.0035 [-0.0047, -0.0032]	0.048 [0.042, 0.063]	
**4**	1514 [1396, 1572]	0.024 [0.023, 0.025]	53376 [53376, 53376]	0.018 [0.016, 0.084]		0.0067 [0.0054, 0.0079]	0.022 [0.021, 0.025]	-0.0037 [-0.0042, -0.0034]	0.04 [0.035, 0.05]	
**6**	1411 [1312, 1591]	0.024 [0.023, 0.025]	53376 [30690, 53376]	0.031 [0.011, 0.09]		0.0086 [0.0063, 0.011]	0.019 [0.017, 0.022]	-0.0042 [-0.0061, -0.0033]	0.031 [0.027, 0.04]	
**Multiple-dose model with varying f_s and γ_d**										
**8**	1572 [1289, 1676]	0.021 [0.02, 0.024]	53376 [36130, 56316]	0.0170 [0.0009, 0.0310]	0.1100 [0.029, 0.20]	0.0093 [0.0010, 0.0160]	0.0020 [0.0160, 0.0230]	-0.0036 [-0.0043, -0.0027]	0.0340 [0.0280, 0.0440]	0.0300 [0.0120, 0.0440]
**10**	1573 [1382, 1687]	0.022 [0.02, 0.025]	53376 [36130, 56316]	0.021 [0.0055, 0.037]	0.13 [0.051, 0.23]	0.0094 [0.0077, 0.015]	0.019 [0.015, 0.022]	-0.0034 [-0.0046, -0.0027]	0.032 [0.024, 0.039]	0.024 [0.014, 0.034]
**12**	1372 [1209, 1479]	0.022 [0.020, 0.025]	53376 [46045, 67004]	0.016 [0.0083, 0.033]	0.16 [0.079, 0.24]	0.0098 [0.0084, 0.012]	0.02 [0.017, 0.024]	-0.0031 [-0.0036, -0.0021]	0.041 [0.035, 0.051]	0.016 [0.0089, 0.032]
**14**	1428 [1327, 1850]	0.022 [0.020, 0.025]	58091 [37433, 73197]	0.025 [0.0066, 0.042]	0.082 [0.046, 0.26]	0.01 [0.0078, 0.012]	0.019 [0.0038, 0.024]	-0.0031 [-0.0048, -0.0017]	0.042 [0.025, 0.065]	0.014 [0.011, 0.026]
**16**	1560 [1350, 1684]	0.023 [0.021, 0.025]	53376 [42964, 81236]	0.032 [0.016, 0.04]	0.13 [0.075, 0.25]	0.01 [0.007, 0.012]	0.019 [0.013, 0.021]	-0.0039 [-0.0049, -0.0031]	0.04 [0.028, 0.047]	0.018 [0.012, 0.038]

Supplementary Table S9. Median and range of the multiple-dose model parameters obtained from the fits to the time courses resulting from cells treated with two consecutive doses of 75 nM doxorubicin delivered at varying inter-treatment intervals (n = 12 per tested interval; Experiment 2 in Table 1 of the main text).

Interval [d]	Quality of fit metrics			
	**NRMSE [%]**	R^2	**PCC**	**CCC**
**Multiple-dose model with constant parameters**				
**0**	4.9 [3.2, 13.5]	0.995 [0.948, 0.998]	0.998 [0.974, 0.999]	0.994 [0.969, 0.995]
**2**	4.39 [3.26, 4.76]	0.996 [0.992, 0.998]	0.998 [0.996, 0.999]	0.994 [0.992, 0.995]
**4**	6.12 [5.03, 7.27]	0.991 [0.979, 0.994]	0.995 [0.990, 0.997]	0.991 [0.986, 0.993]
**6**	5.27 [2.74, 9.47]	0.992 [0.971, 0.999]	0.996 [0.986, >0.999]	0.992 [0.982, 0.996]
**Multiple-dose model with varying f_s and γ_d**				
**8**	4.57 [3.59, 6.32]	0.993 [0.983, 0.997]	0.997 [0.992, 0.999]	0.992 [0.988, 0.995]
**10**	4.08 [3.59, 6.57]	0.992 [0.974, 0.998]	0.996 [0.988, 0.999]	0.992 [0.983, 0.995]
**12**	4.54 [3.44, 10.2]	0.995 [0.963, 0.998]	0.998 [0.982, 0.999]	0.994 [0.977, 0.995]
**14**	4.39 [2.79, 14.3]	0.996 [0.769, 0.999]	0.998 [0.911, >0.999]	0.994 [0.898, 0.996]
**16**	4.38 [3.12, 7.04]	0.996 [0.971, 0.998]	0.998 [0.986, 0.999]	0.994 [0.981, 0.995]

Supplementary Table S10. Median and range of the quality of fit metrics for the multiple-dose model fits to the time courses resulting from cells treated with two consecutive doses of 75 nM doxorubicin delivered at varying inter-treatment intervals (n = 12 per tested interval; Experiment 2 in Table 1 of the main text).

Interval [d]	**0**	**2**	**4**	**6**	**8**	**10**	**12**	**14**	**16**
**0**	-	0.2145	0.4705	0.0999	0.0783	**0.0226**	**0.0166**	**0.0102**	**0.0061**
**2**	-	-	**0.0029**	**1.96×10^{-4}**	**1.55×10^{-4}**	**3.66×10^{-5}**	**3.66×10^{-5}**	**3.66×10^{-5}**	**3.66×10^{-5}**
**4**	-	-	-	**0.0102**	**0.0029**	**3.66×10^{-5}**	**3.66×10^{-5}**	**3.66×10^{-5}**	**3.66×10^{-5}**
**6**	-	-	-	-	0.5067	**0.0262**	**0.0035**	**0.0120**	**0.0011**
**8**	-	-	-	-	-	0.1749	0.0783	**0.0404**	**0.0024**
**10**	-	-	-	-	-	-	0.5834	0.4705	0.0690
**12**	-	-	-	-	-	-	-	0.5444	0.1124
**14**	-	-	-	-	-	-	-	-	0.5444
**16**	-	-	-	-	-	-	-	-	-

Supplementary Table S11. P-values from two-sided Wilcoxon rank sum tests comparing the observed final tumor cell numbers for every distinct combination of the varying inter-treatment interval datasets (n = 12 per tested interval; Experiment 2 in Table 1 of the main text). Values bolded in red indicate $p < 0.05$.

Number of doses	Initial guess and bounds: 2-day inter-treatment interval							
	N_0 [cells]	g_0 [h^{-1}]	θ_{Dox} [cells]	f_s [-]	g_s [h^{-1}]	g_d [h^{-1}]	k_d [h^{-1}]	γ_d [h^{-1}]
**1**	$N_{0,obs}$ [1000, 2500]	0.0234 [0.0225, 0.05]	60000 [20000, 90000]	0.0337 [0, 0.05]	0.0101 [1×10^{-4}, 0.05]	0.0277 [1×10^{-4}, 0.05]	-1×10^{-5} [-0.05, 0]	0.0325 [0, 1]
**2**	〃	〃	〃	0.01 [0, 0.05]	0.0025 [1×10^{-4}, 0.05]	0.0025 [1×10^{-4}, 0.05]	-0.015 [-0.05, 0]	〃
**3**	〃	〃	〃	0.001 [0, 0.05]	〃	〃	〃	0.05 [0, 1]
**4**	〃	〃	〃	1×10^{-5} [0, 0.05]	〃	〃	〃	0.08 [0, 1]
**5**	〃	〃	〃	1×10^{-9} [0, 0.05]	1.5×10^{-4} [1×10^{-4}, 0.05]	1.5×10^{-4} [1×10^{-4}, 0.05]	-0.035 [-0.05, 0]	0.75 [0, 1]

Supplementary Table S12. Parameter initial guesses and bounds used for fitting the multiple-dose model to the time courses resulting from cells treated with a varying number of 75 nM doxorubicin doses delivered at 2-day inter-treatment intervals (n = 12 per tested number of doses; Experiment 3 in Table 1 of the main text). A ditto mark (〃) indicates that the initial guess and bounds are identical to the values in the cells above.

Number of Doses	Parameter values: 2-day inter-treatment interval							
	N_0 [cells]	g_0 [h^{-1}]	θ_{Dox} [cells]	f_s [-]	g_s [h^{-1}]	g_d [h^{-1}]	k_d [h^{-1}]	γ_d [h^{-1}]
**1**	1477 [1276, 1712]	0.024 [0.023, 0.028]	59448 [34132, 87537]	0.02 [0.004, 0.029]	0.01 [0.0084, 0.015]	0.021 [0.0088, 0.03]	-0.006 [-0.0089, -0.003]	0.037 [0.014, 0.064]
**2**	1313 [1194, 1503]	0.028 [0.027, 0.029]	59448 [59448, 59448]	0.0088 [1×10^{-7}, 0.05]	0.0062 [0.0047, 0.0079]	0.019 [0.012, 0.039]	-0.004 [-0.0042, -0.0034]	0.08 [0.059, 0.2]
**3**	1278 [1081, 1456]	0.028 [0.026, 0.029]	59448 [59448, 59448]	0.0066 [1×10^{-7}, 0.016]	0.013 [0.0099, 0.016]	0.029 [0.017, 0.038]	-0.005 [-0.005, -0.0041]	0.13 [0.088, 0.15]
**4**	1196 [1032, 1380]	0.028 [0.026, 0.029]	59448 [59448, 59448]	6×10^{-5} [2×10^{-14}, 0.0086]	1×10^{-4} [1×10^{-4}, 0.017]	0.027 [0.023, 0.033]	-0.005 [-0.0058, -0.0045]	0.11 [0.074, 0.15]
**5**	1140 [1051, 1305]	0.028 [0.026, 0.029]	59448 [59448, 59448]	0.031 [0.023, 0.047]	0.015 [0.013, 0.017]	0.035 [0.028, 0.05]	-0.006 [-0.0068, -0.0061]	0.11 [0.086, 0.18]

Supplementary Table S13. Median and range of the multiple-dose model parameters obtained from the fits to the time courses resulting from cells treated with a varying number of 75 nM doxorubicin doses delivered at 2-day inter-treatment intervals (n = 12 per tested number of doses; Experiment 3 in Table 1 of the main text).

Number of doses	Quality of fit metrics: 2-day inter-treatment interval			
	**NRMSE [%]**	$\boldsymbol{R^2}$	**PCC**	**CCC**
**1**	3.5 [2.72, 5.79]	0.998 [0.992, 0.999]	0.999 [0.996, >0.999]	0.993 [0.99, 0.994]
**2**	4.72 [4.14, 19.1]	0.997 [0.992, 0.999]	0.998 [0.934, 0.999]	0.994 [0.928, 0.995]
**3**	12.1 [10.1, 13.7]	0.985 [0.992, 0.999]	0.993 [0.991, 0.995]	0.988 [0.987, 0.991]
**4**	16.2 [15.1, 17.5]	0.975 [0.992, 0.999]	0.989 [0.987, 0.991]	0.984 [0.981, 0.986]
**5**	14.8 [13, 16.4]	0.983 [0.992, 0.999]	0.993 [0.991, 0.995]	0.988 [0.986, 0.99]

Supplementary Table S14. Median and range of the quality of fit metrics for the multiple-dose model fits to the time courses resulting from cells treated with a varying number of 75 nM doxorubicin doses delivered at 2-day inter-treatment intervals (n = 12 per tested number of doses; Experiment 3 in Table 1 of the main text).

Number of doses	**1**	**2**	**3**	**4**	**5**
**1**	**-**	**3.66×10^{-5}**	**3.64×10^{-5}**	**3.64×10^{-5}**	**3.64×10^{-5}**
**2**	**-**	**-**	**0.0165**	**0.0110**	**4.68×10^{-5}**
**3**	**-**	**-**	**-**	0.7289	**1.08×10^{-4}**
**4**	**-**	**-**	**-**	**-**	**0.0079**
**5**	**-**	**-**	**-**	**-**	**-**

Supplementary Table S15. P-values from 2-sided Wilcoxon rank sum tests comparing the final tumor cell numbers for every distinct combination of the varying dose number datasets with a 2-day inter-treatment interval. (n = 12 per tested number of doses; Experiment 3 in Table 1 of the main text). Values bolded in red indicate $p < 0.05$.

Number of Doses	Initial guess and bounds: 2-week inter-treatment interval					
	$\boldsymbol{N_0}$ **[cells]**	$\boldsymbol{g_0}$ **[h^{-1}]**	$\boldsymbol{\theta_{Dox}}$ **[cells]**	$\boldsymbol{g_s}$ **[h^{-1}]**	$\boldsymbol{g_d}$ **[h^{-1}]**	$\boldsymbol{k_d}$ **[h^{-1}]**
**1**	$N_{0,obs}$<br>[900, 3000]	0.0275<br>[0.02, 0.05]	80000<br>[15000, 90000]	0.02<br>[0.001, 0.05]	0.0275<br>[0.0125, 0.075]	-0.0075<br>[-0.01, -0.001]
**2**	〃	〃	〃	〃	〃	〃
**3**	〃	〃	〃	〃	〃	〃
**4**	〃	〃	〃	〃	〃	〃
**5**	〃	〃	〃	〃	〃	〃

Supplementary Table S16. Initial guesses and bounds of the constant parameters used for fitting the multiple-dose model to the time courses resulting from cells treated with a varying number of 75 nM doxorubicin doses delivered at 2-week inter-treatment intervals (n = 12 per tested number of doses; Experiment 3 in Table 1 of the main text). A ditto mark (〃) indicates that the initial guess and bounds are identical to the values in the cells above.

Initial guess & bounds: 2-week inter-treatment interval				
$\boldsymbol{f_s^1}$ **[-]**	$\boldsymbol{f_s^2}$ **[-]**	$\boldsymbol{f_s^3}$ **[-]**	$\boldsymbol{f_s^4}$ **[-]**	$\boldsymbol{f_s^5}$ **[-]**
0.001<br>[0, 1]	0.005<br>[0, 0.5]	0.005<br>[0, 0.5]	0.005<br>[0, 0.5]	0.005<br>[0, 0.5]
$\boldsymbol{\gamma_d^1}$ **[h^{-1}]**	$\boldsymbol{\gamma_d^2}$ **[h^{-1}]**	$\boldsymbol{\gamma_d^3}$ **[h^{-1}]**	$\boldsymbol{\gamma_d^4}$ **[h^{-1}]**	$\boldsymbol{\gamma_d^5}$ **[h^{-1}]**
1/50<br>[0, 1/15]	1/50<br>[0, 1/15]	1/50<br>[0, 1/15]	1/50<br>[0, 1/15]	1/50<br>[0, 1/15]

Supplementary Table S17. Initial guesses and bounds of f_s and γ_d for each doxorubicin dose used for fitting the multiple-dose model to the time courses resulting from cells treated with a varying number of 75 nM doxorubicin doses delivered at 2-week inter-treatment intervals (n = 12 per tested number of doses; Experiment 3 in Table 1 of the main text). A ditto mark (〃) indicates that the initial guess and bounds are identical to the values in the cells above.

Number of doses	Parameter values: 2-week inter-treatment interval					
	$\boldsymbol{N_0}$ **[cells]**	$\boldsymbol{g_0}$ **[h^{-1}]**	$\boldsymbol{\theta_{Dox}}$ **[cells]**	$\boldsymbol{g_s}$ **[h^{-1}]**	$\boldsymbol{g_d}$ **[h^{-1}]**	$\boldsymbol{k_d}$ **[h^{-1}]**
**1**	1626<br>[1437, 1791]	0.024<br>[0.023, 0.025]	52460<br>[41191, 73604]	0.013<br>[0.011, 0.015]	0.02<br>[0.014, 0.023]	-0.0053<br>[-0.0073, -0.0032]
**2**	1322<br>[1066, 1568]	0.026<br>[0.024, 0.027]	55086<br>[32671, 62376]	0.015<br>[0.013, 0.018]	0.014<br>[0.013, 0.016]	-0.0026<br>[-0.0037, -0.0023]
**3**	1345<br>[1051, 1553]	0.025<br>[0.022, 0.027]	68390<br>[62376, 78206]	0.018<br>[0.016, 0.019]	0.014<br>[0.013, 0.023]	-0.0023<br>[-0.0029, -0.001]
**4**	1182<br>[1017, 1417]	0.024<br>[0.021, 0.026]	71551<br>[54867, 77778]	0.018<br>[0.016, 0.02]	0.013<br>[0.013, 0.018]	-0.0014<br>[-0.0021, -0.001]
**5**	1114<br>[901, 1287]	0.024<br>[0.021, 0.025]	70227<br>[57909, 78940]	0.019<br>[0.016, 0.019]	0.015<br>[0.013, 0.019]	-0.0014<br>[-0.003, -0.001]

Supplementary Table S18. Median and range of the constant model parameters from the multiple-dose model fits to the time courses resulting from cells treated with a varying number of 75 nM doxorubicin doses delivered at 2-week inter-treatment intervals (n = 12 per tested number of doses; Experiment 3 in Table 1 of the main text).

Parameter values: 2-week inter-treatment interval				
$\boldsymbol{f_s^1}$ **[-]**	$\boldsymbol{f_s^2}$ **[-]**	$\boldsymbol{f_s^3}$ **[-]**	$\boldsymbol{f_s^4}$ **[-]**	$\boldsymbol{f_s^5}$ **[-]**
0.003<br>[2×10^{-4}, 0.057]	0.0076<br>[0.0032, 0.070]	0.0094<br>[0.0028, 0.035]	0.017<br>[0.0068, 0.051]	0.0061<br>[0.0017, 0.033]
$\boldsymbol{\gamma_d^1}$ **[h^{-1}]**	$\boldsymbol{\gamma_d^2}$ **[h^{-1}]**	$\boldsymbol{\gamma_d^3}$ **[h^{-1}]**	$\boldsymbol{\gamma_d^4}$ **[h^{-1}]**	$\boldsymbol{\gamma_d^5}$ **[h^{-1}]**
0.039<br>[0.020, 0.067]	0.0097<br>[0.0049, 0.017]	0.012<br>[0.0072, 0.021]	0.014<br>[0.0092, 0.032]	0.020<br>[0.012, 0.043]

Supplementary Table S19. Median and range of f_s and γ_d for each doxorubicin dose from the multiple-dose model fits to the time courses resulting from cells treated with a varying number of 75 nM doxorubicin doses delivered at 2-week inter-treatment intervals (n = 12 per tested number of doses; Experiment 3 in Table 1 of the main text).

Number of doses	Quality of fit metrics: 2-week inter-treatment interval			
	**NRMSE [%]**	R^2	**PCC**	**CCC**
**1**	2.38 [2.05, 3.11]	0.999 [0.998, >0.999]	>0.999 [0.999, >0.999]	0.993 [0.993, 0.994]
**2**	3.45 [2.13, 8.57]	0.996 [0.932, 0.999]	0.998 [0.969, 0.999]	0.993 [0.959, 0.995]
**3**	3.69 [2.90, 6.80]	0.993 [0.952, 0.997]	0.997 [0.978, 0.999]	0.992 [0.971, 0.995]
**4**	3.51 [1.91, 7.02]	0.996 [0.975, 0.999]	0.998 [0.988, >0.999]	0.995 [0.984, 0.997]
**5**	3.10 [1.99, 6.40]	0.996 [0.934, 0.999]	0.998 [0.969, 0.999]	0.995 [0.963, 0.997]

Supplementary Table S20. Median and range of the quality of fit metrics for the multiple-dose model fits to time courses resulting from cells treated with a varying number of 75 nM doxorubicin doses delivered at 2-week inter-treatment intervals (n = 12 per tested number of doses; Experiment 3 in Table 1 of the main text).

Number of doses	**1**	**2**	**3**	**4**	**5**
**1**	**-**	**9.73×10^{-5}**	**4.69×10^{-5}**	**2.46×10^{-5}**	**7.66×10^{-5}**
**2**	**-**	**-**	0.4357	0.7508	0.5444
**3**	**-**	**-**	**-**	0.1572	0.4025
**4**	**-**	**-**	**-**	**-**	0.3708
**5**	**-**	**-**	**-**	**-**	**-**

Supplementary Table S21. P-values from 2-sided Wilcoxon rank sum tests comparing the final tumor cell numbers for every distinct combination of the varying dose number datasets with a 2-week treatment interval. (n = 12 per tested number of doses; Experiment 3 in Table 1 of the main text). Values bolded in red indicate $p < 0.05$.

Supplementary Figures

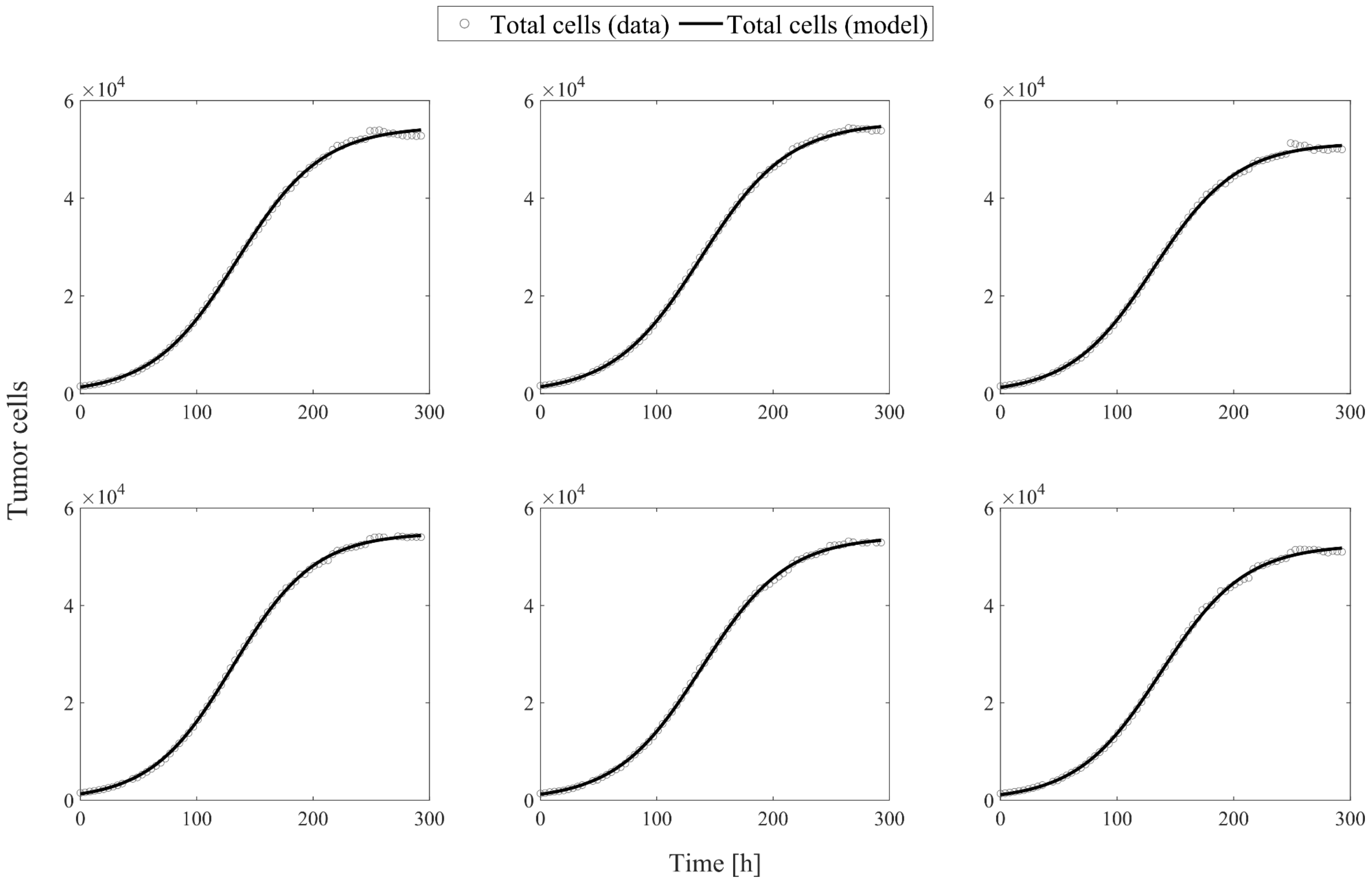

Supplementary Figure S1. Logistic growth model fits for all of the untreated tumor cell time courses (i.e., for a doxorubicin concentration of 0 nM in Experiment 1 in Table 1 of the main text; n = 6).

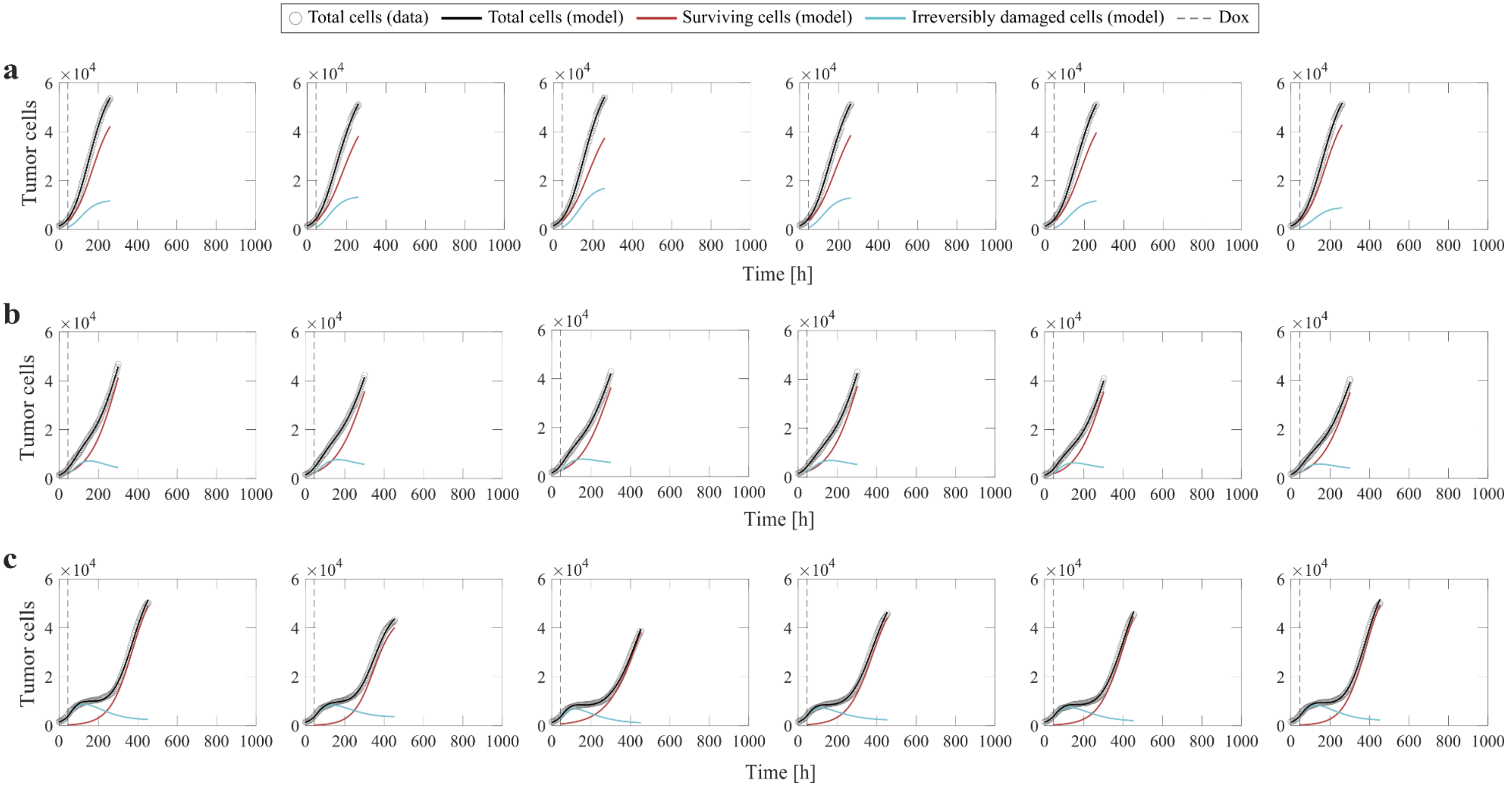

Supplementary Figure S2. Single-dose model fits for all of the time courses resulting from cells treated with a single dose of doxorubicin at concentrations ranging from 10 to 300 nM (n = 6 for each concentration; Experiment 1 in Table 1 of the main text). (**a**) 10 nM; (**b**) 20 nM; (**c**) 35 nM.

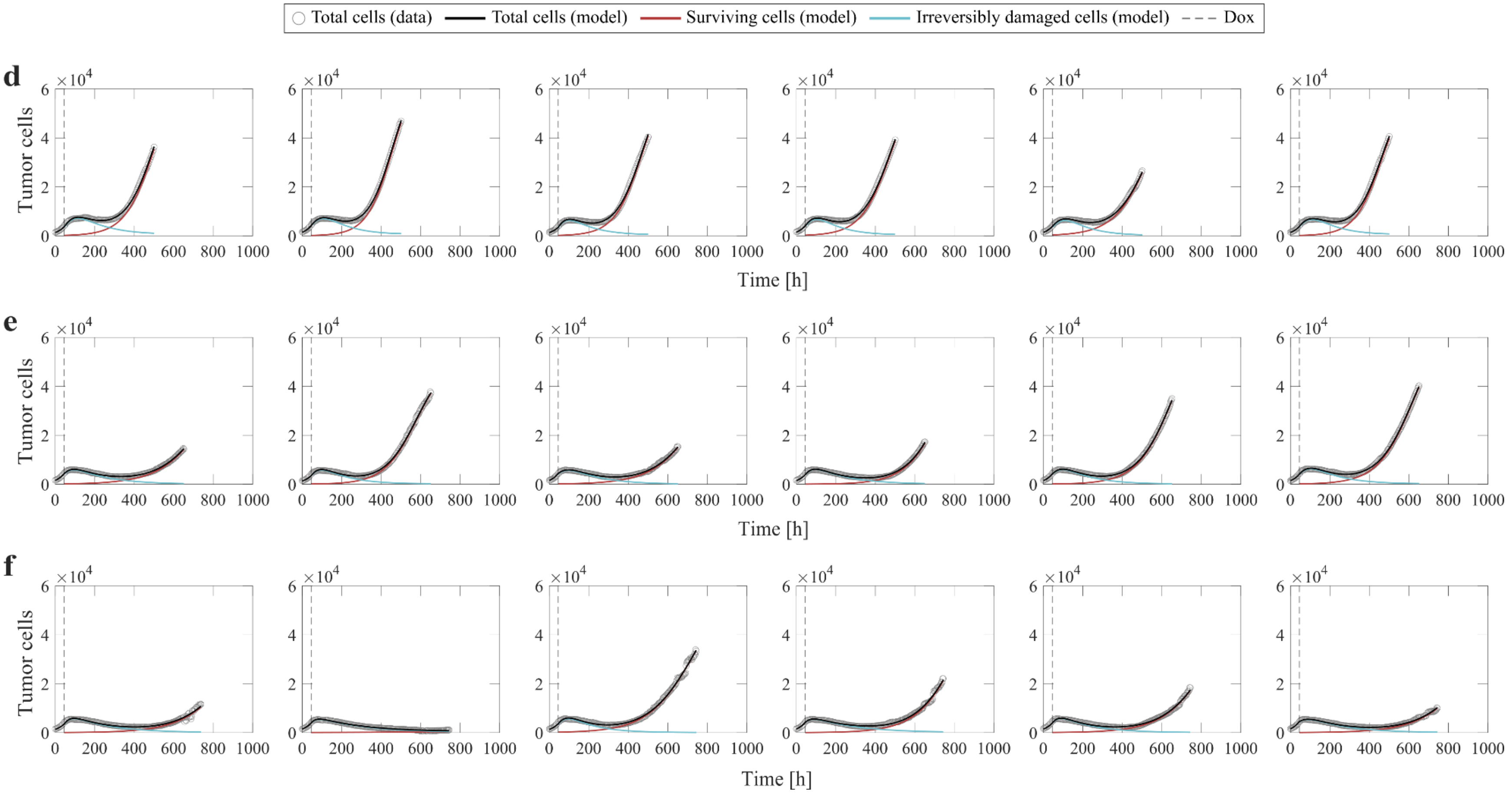

Supplementary Figure S2 (continued). Single-dose model fits for all of the time courses resulting from cells treated with a single dose of doxorubicin at concentrations ranging from 10 to 300 nM (n = 6 for each concentration; Experiment 1 in Table 1 of the main text). (**d**) 50nM; (**e**) 75nM; (**f**) 100 nM.

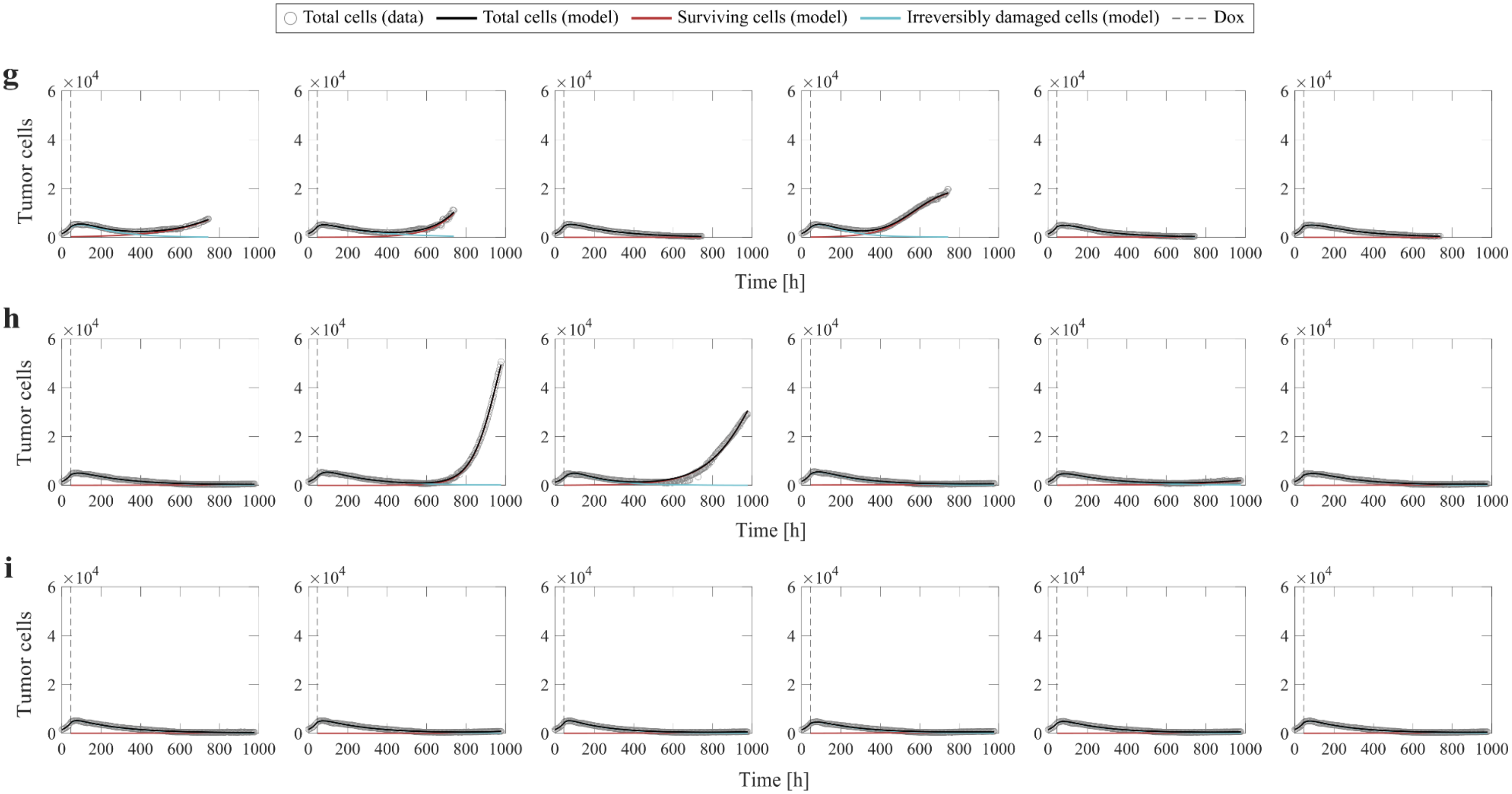

Supplementary Figure S2 (continued). Single-dose model fits for all of the time courses resulting from cells treated with a single dose of doxorubicin at concentrations ranging from 10 to 300 nM (n = 6 for each concentration; Experiment 1 in Table 1 of the main text). (**g**) 125 nM; (**h**) 150 nM; (**i**) 300 nM.

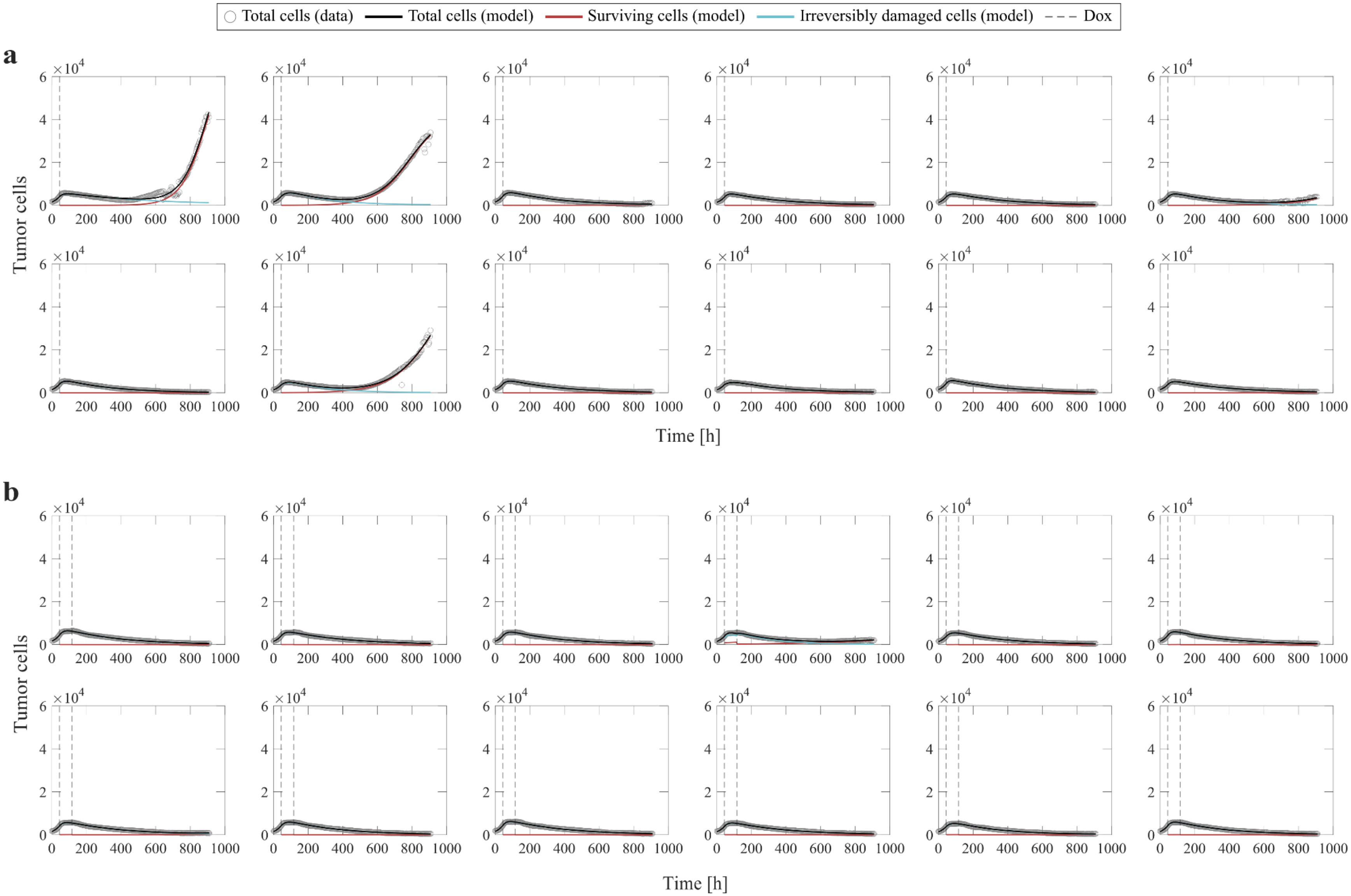

Supplementary Figure S3. Multiple-dose model fits for all of the time courses resulting from cells treated with two consecutive doses of 75 nM doxorubicin delivered at varying inter-treatment intervals (n = 12 for each interval; Experiment 2 in Table 1 of the main text). (**a**) 0 days; (**b**) 2 days. For (**a**)-(**d**), the multiple-dose model with constant parameters was used to fit the datasets, while for (**e**)-(**i**), the multiple-dose model with varying f_s and γ_d was used.

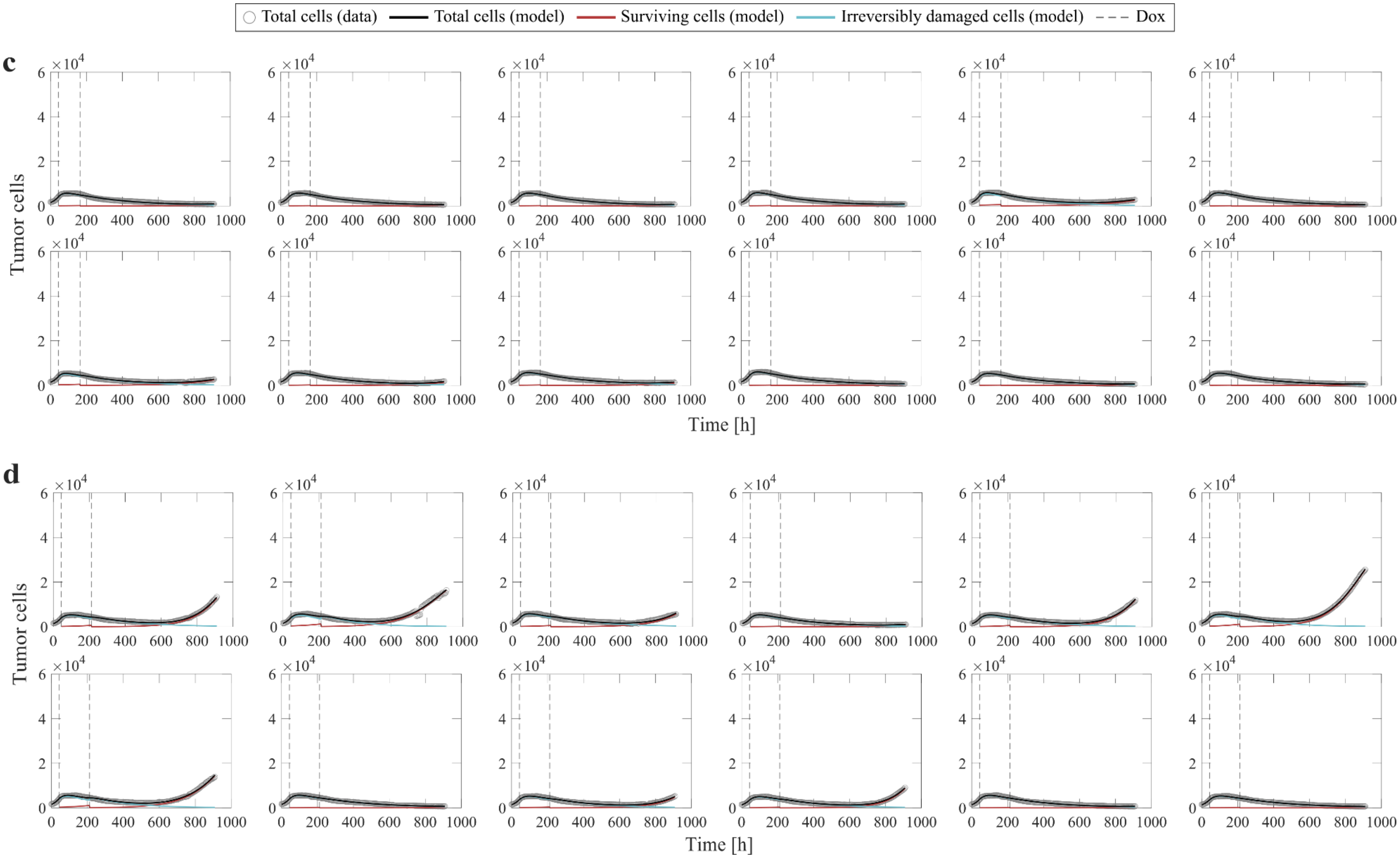

Supplementary Figure S3 (continued). Multiple-dose model fits for all of the time courses resulting from cells treated with two consecutive doses of 75 nM doxorubicin delivered at varying inter-treatment intervals (n = 12 for each interval; Experiment 2 in Table 1 of the main text). (**c**) 4 days; (**d**) 6 days. For (**a**)-(**d**), the multiple-dose model with constant parameters was used to fit the datasets, while for (**e**)-(**i**), the multiple-dose model with varying f_s and γ_d was used.

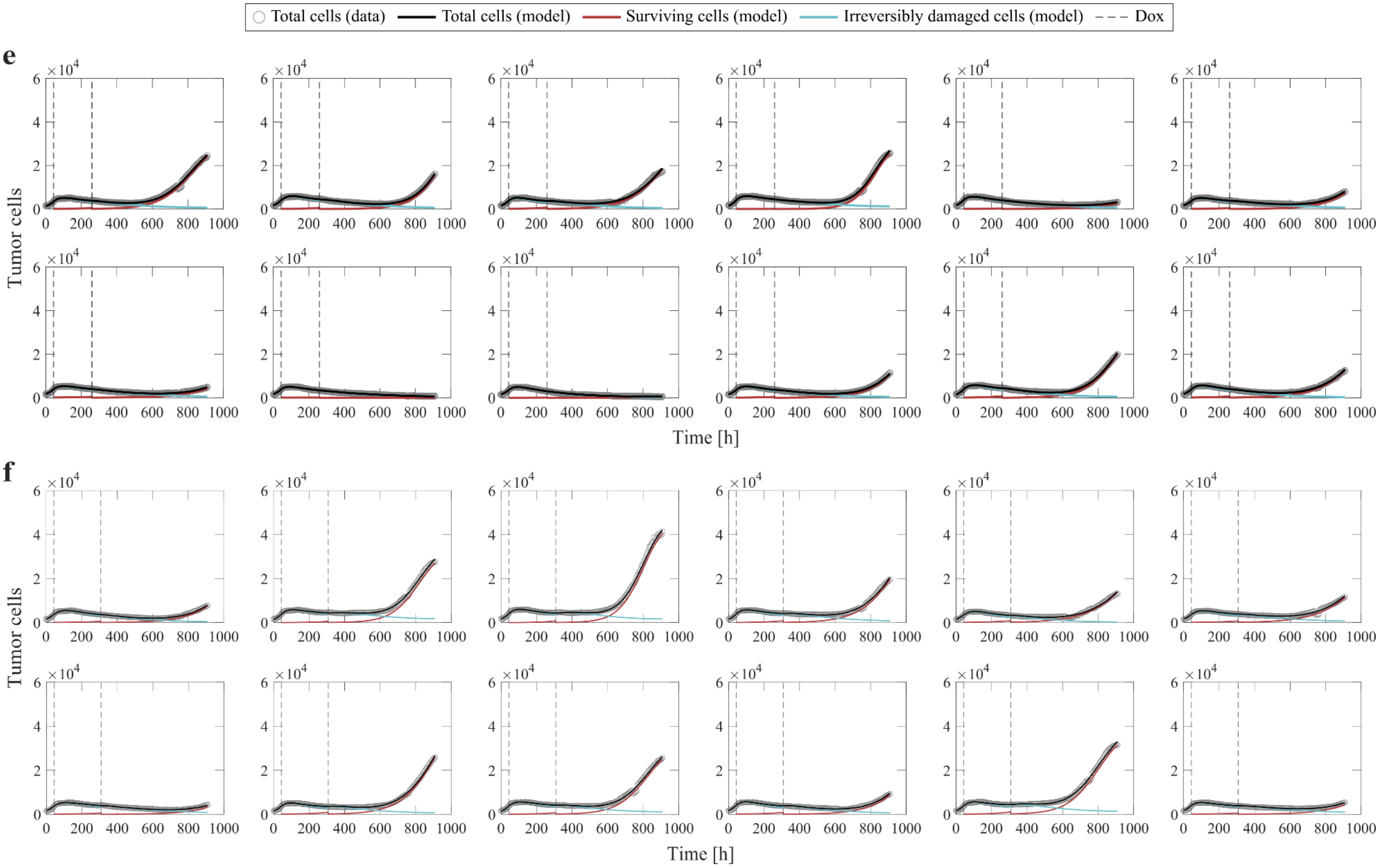

Supplementary Figure S3 (continued). Multiple-dose model fits for all of the time courses resulting from cells treated with two consecutive doses of 75 nM doxorubicin delivered at varying inter-treatment intervals (n = 12 for each interval; Experiment 2 in Table 1 of the main text). (**e**) 8 days; (**f**) 10 days. For (**a**)-(**d**), the multiple-dose model with constant parameters was used to fit the datasets, while for (**e**)-(**i**), the multiple-dose model with varying f_s and γ_d was used.

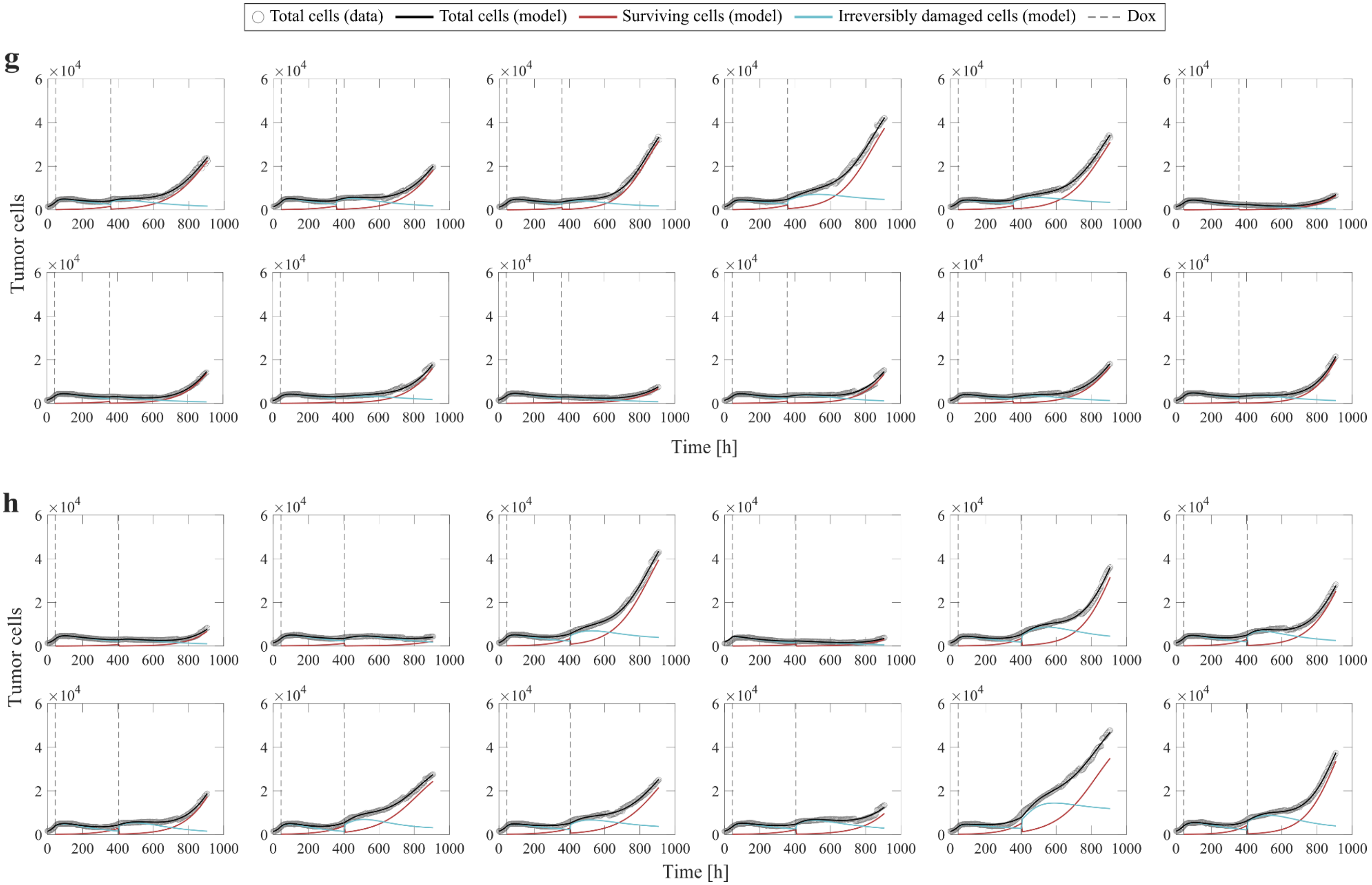

Supplementary Figure S3 (continued). Multiple-dose model fits for all of the time courses resulting from cells treated with two consecutive doses of 75 nM doxorubicin delivered at varying inter-treatment intervals (n=12 for each interval; Experiment 2 in Table 1 of the main text). (**g**) 12 days; (**h**) 14 days. For (**a**)-(**d**), the multiple-dose model with constant parameters was used to fit the datasets, while for (**e**)-(**i**), the multiple-dose model with varying f_s and γ_d was used.

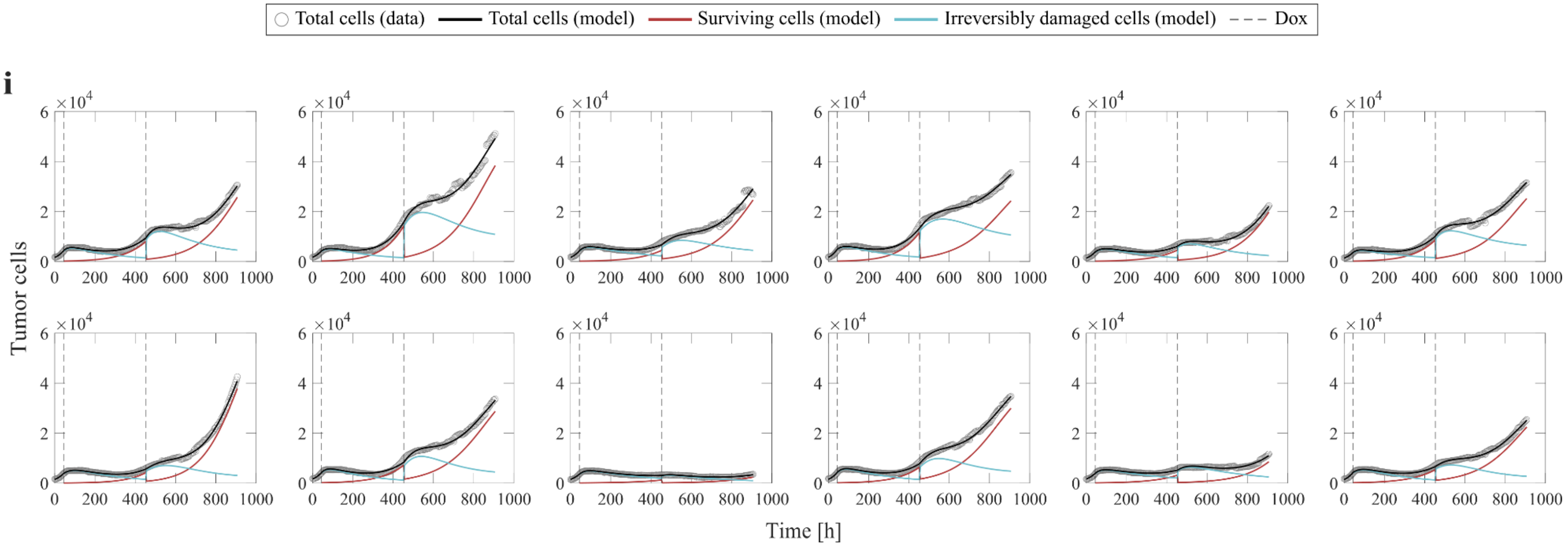

Supplementary Figure S3 (continued). Multiple-dose model fits for all of the time courses resulting from cells treated with two consecutive doses of 75 nM doxorubicin delivered at varying inter-treatment intervals (n=12 for each interval; Experiment 2 in Table 1 of the main text). (**i**) 16 days. For (**a**)-(**d**), the multiple-dose model with constant parameters was used to fit the datasets, while for (**e**)-(**i**), the multiple-dose model with varying f_s and γ_d was used.

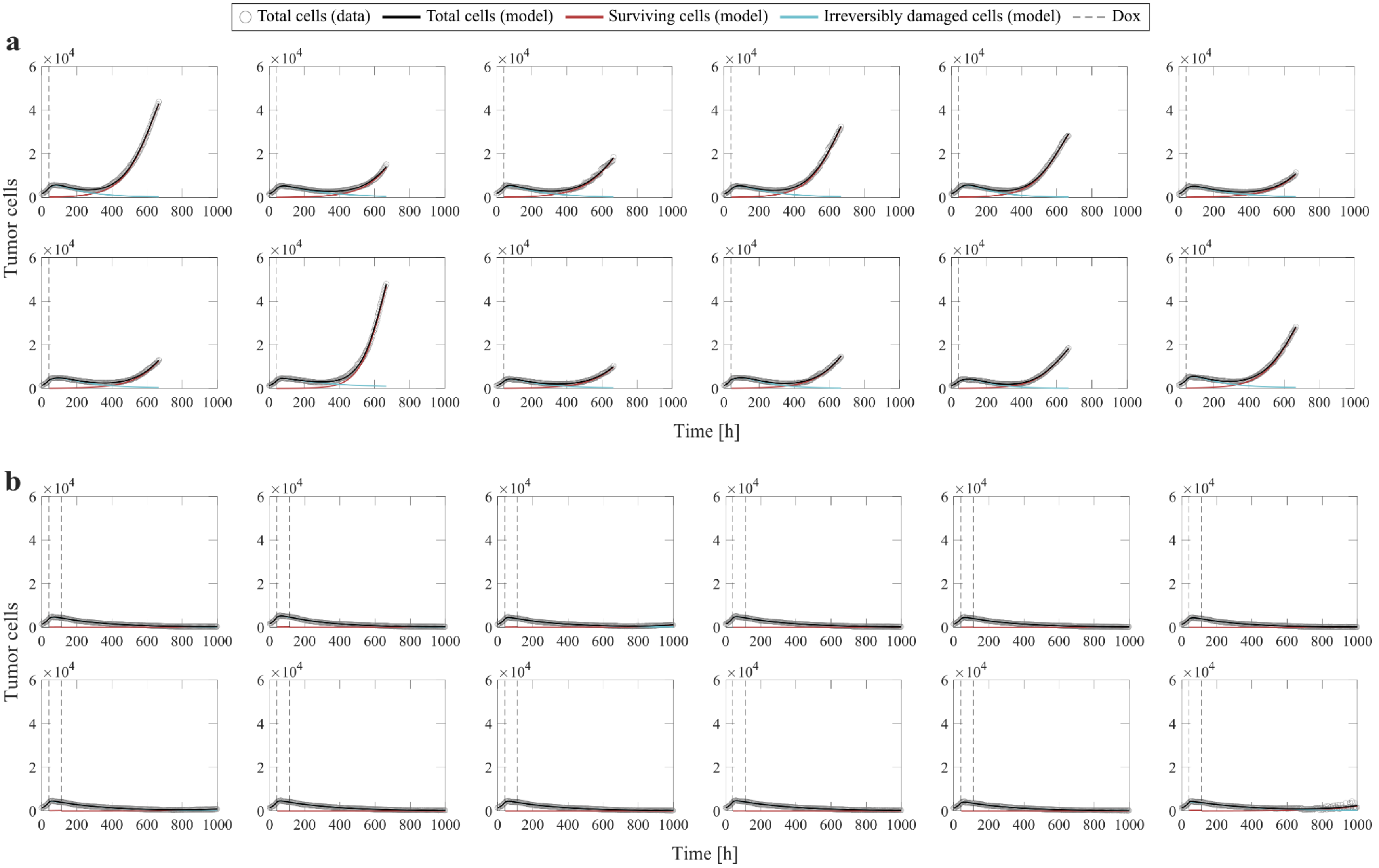

Supplementary Figure S4. Multiple-dose model fits for all of the time courses resulting from cells treated with a varying number of 75 nM doxorubicin doses delivered at 2-day inter-treatment intervals (n = 12 for each total dose number; Experiment 3 in Table 1 of the main text). (**a**) 1 dose; (**b**) 2 doses. The multiple-dose model with constant parameters was used to fit the datasets.

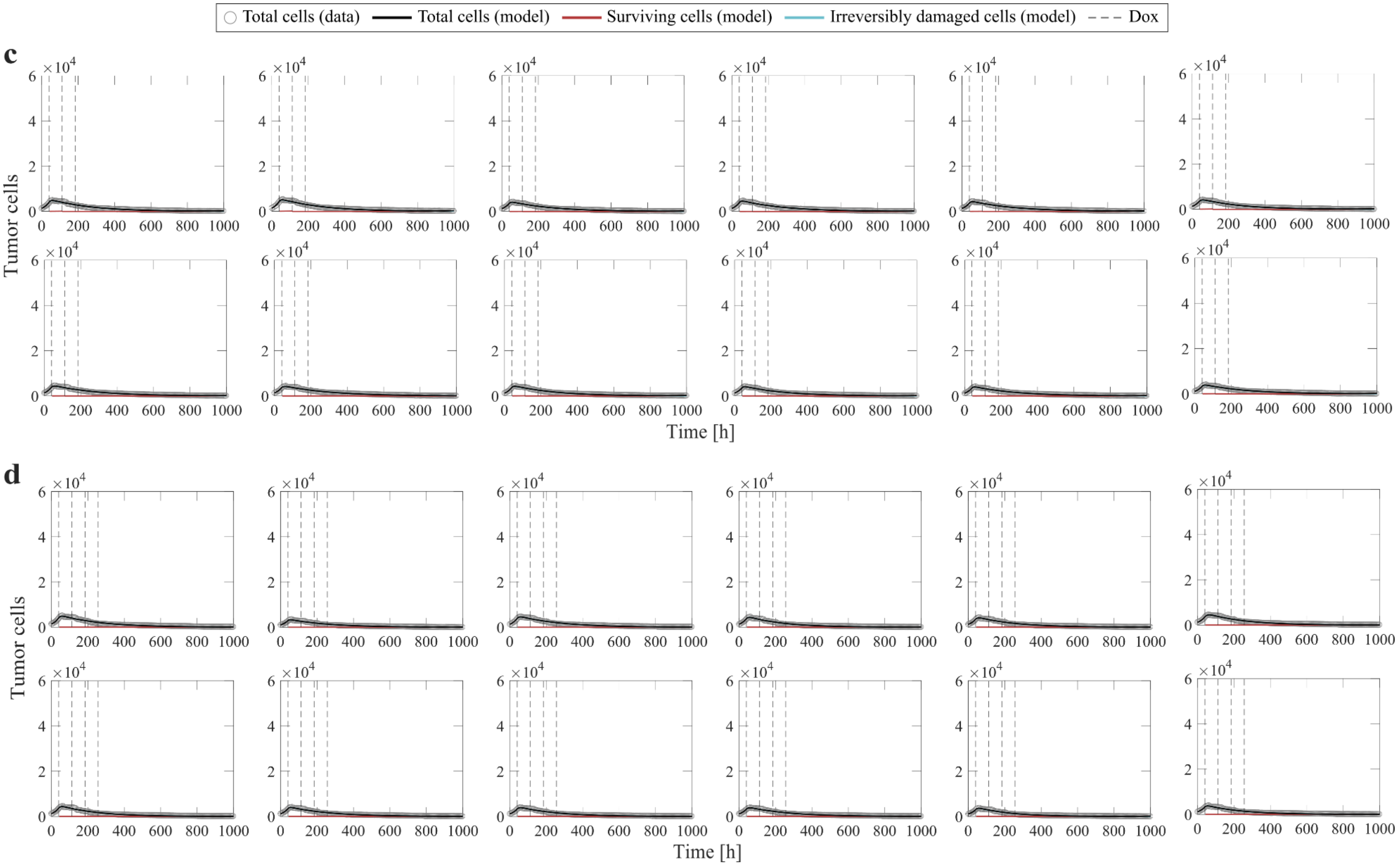

Supplementary Figure S4 (continued). Multiple-dose model fits for all of the time courses resulting from cells treated with a varying number of 75 nM doxorubicin doses delivered at 2-day inter-treatment intervals (n = 12 for each total dose number; Experiment 3 in Table 1 of the main text). **(c)** 3 doses; **(d)** 4 doses. The multiple-dose model with constant parameters was used to fit the datasets.

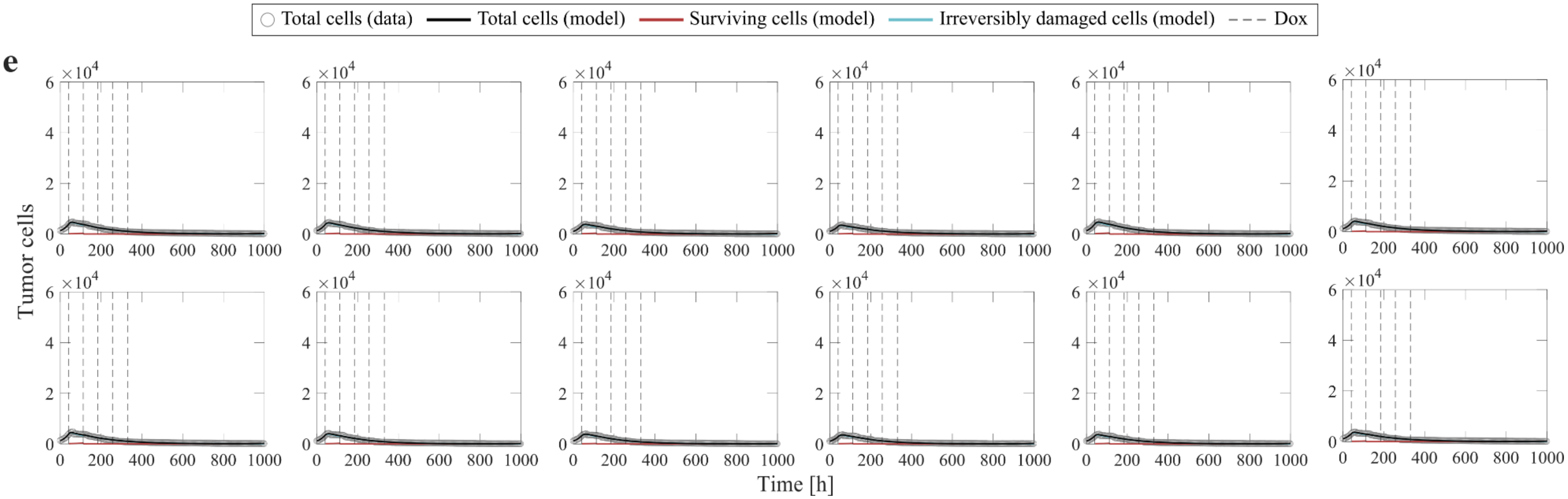

Supplementary Figure S4 (continued). Multiple-dose model for all of the time courses resulting from cells treated with a varying number of 75 nM doxorubicin doses delivered at 2-day inter-treatment intervals (n = 12 for each total dose number; Experiment 3 in Table 1 of the main text). (**e**) 5 doses. The multiple-dose model with constant parameters was used to fit the datasets.

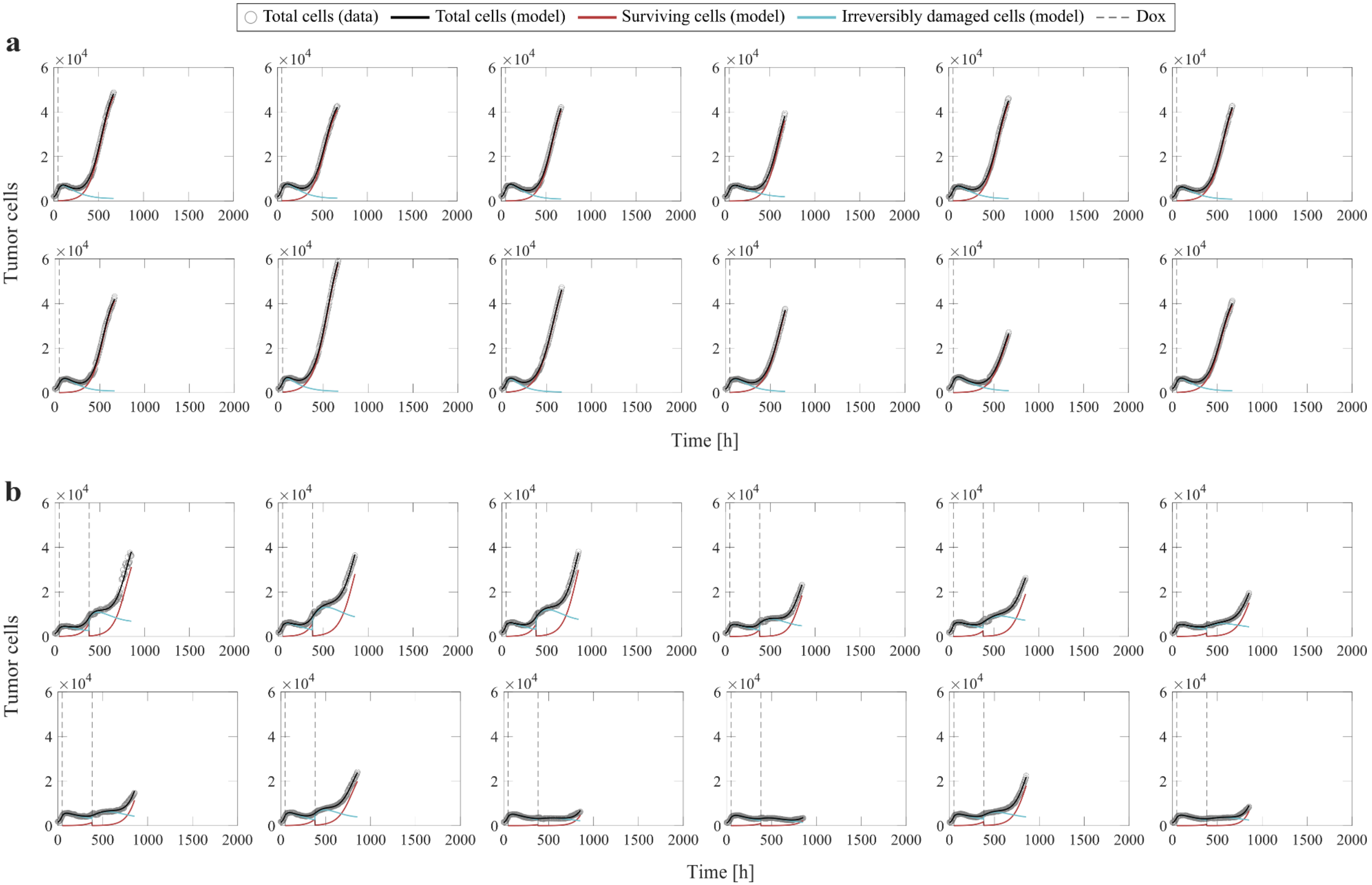

Supplementary Figure S5. Multiple-dose model fits for all of the time courses resulting from cells treated with a varying number of 75 nM doxorubicin doses delivered at 2-week inter-treatment intervals (n = 12 for each total dose number; Experiment 3 in Table 1 of the main text). (**a**) 1 dose; (**b**) 2 doses. The multiple-dose model with varying f_s and γ_d was used to fit the datasets.

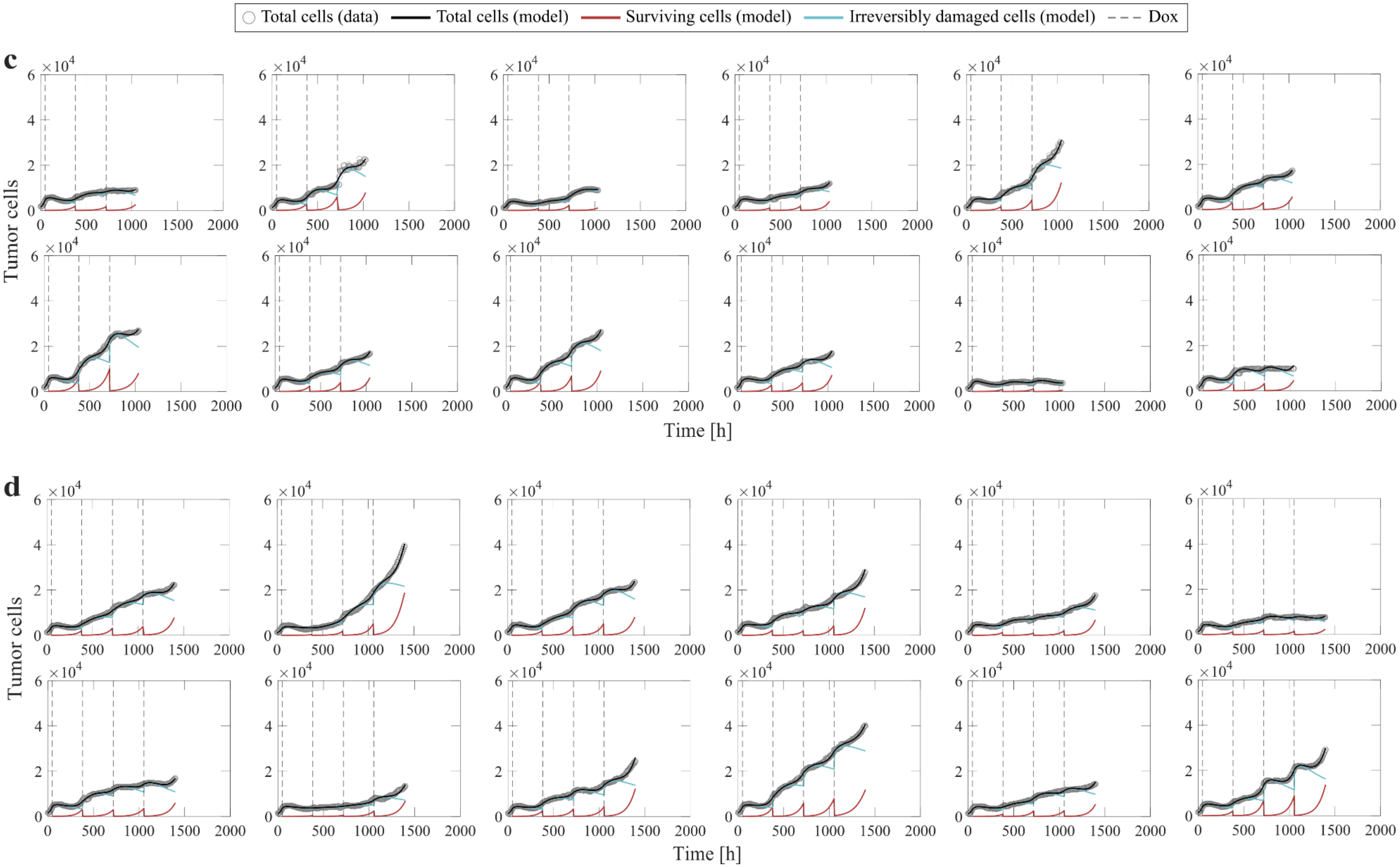

Supplementary Figure S5 (continued). Multiple-dose model fits for all of the time courses resulting from cells treated with a varying number of 75 nM doxorubicin doses delivered at 2-week inter-treatment intervals (n = 12 for each total dose number; Experiment 3 in Table 1 of the main text). **(c)** 3 doses; (**d**) 4 doses. The multiple-dose model with varying f_s and γ_d was used to fit the datasets.

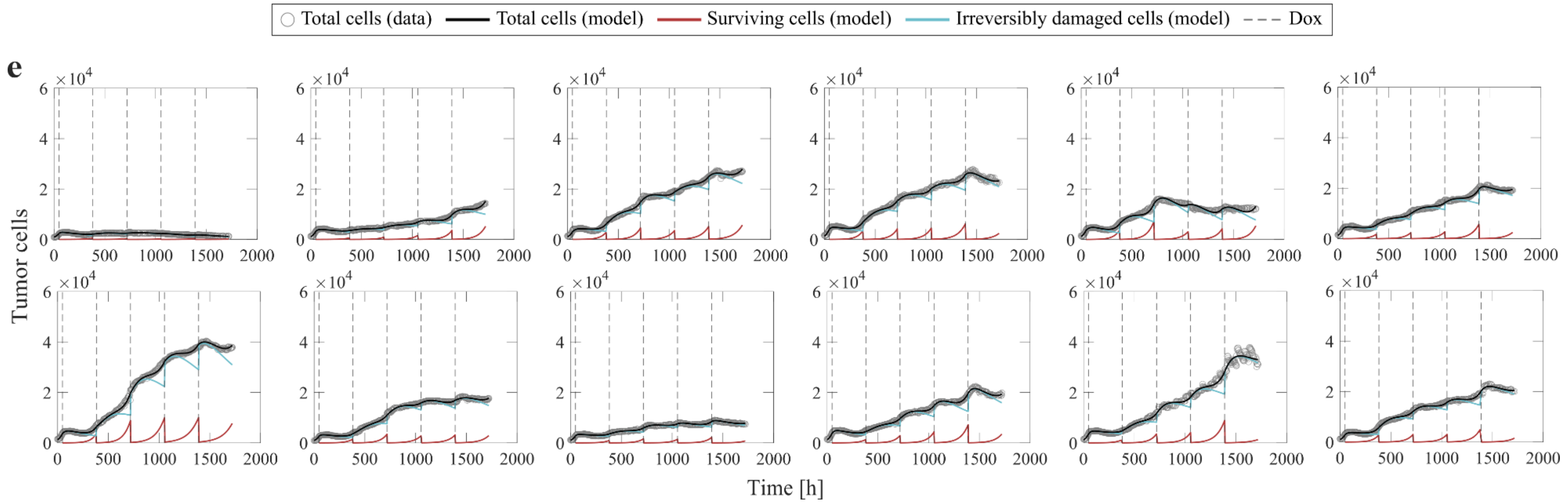

Supplementary Figure S5 (continued). Multiple-dose model fits for all of the time courses resulting from cells treated with a varying number of 75 nM doxorubicin doses delivered at 2-week inter-treatment intervals (n = 12 for each total dose number; Experiment 3 in Table 1 of the main text). (**e**) 5 doses. The multiple-dose model with varying f_s and γ_d was used to fit the datasets.

Appendix A. Data normalization

The procedure to exchange the media during the experiments may result in some cells being accidentally removed. This issue introduces a discontinuity in the measured total tumor cell number time courses that complicates their analysis using biomathematical models (e.g., hampering the model parameterization). As explained in the section *Data normalization* of the main text, the purpose of Eq. (1) is to obtain a correction factor to adjust the total tumor cell counts measured before an accidental removal of tumor cells during media handling, thereby accounting for this cell loss and smoothing the discontinuity in the measured tumor cell counts. To this end, the authors of Ref. [41] proposed a simple *ad hoc* approach: they divide all the data points before the discontinuity by a nonzero, positive constant α such that, after this correction, the slope of the measured total tumor cell count curve at the discontinuity equals the average of the slopes before and after the discontinuity. More specifically, let N_d, N_{d-i}, and N_{d+i} denote the total tumor cell counts measured at times t_d, t_{d-i}, and t_{d+i}, respectively, where t_d is the time of the discontinuity introduced by media handling. Then, the constant α must satisfy:

$$\frac{N_d - \frac{N_{d-1}}{\alpha}}{t_d - t_{d-1}} = \frac{1}{2}\left[\frac{\frac{N_{d-1}}{\alpha} - \frac{N_{d-2}}{\alpha}}{t_{d-1} - t_{d-2}} + \frac{N_{d+1} - N_d}{t_{d+1} - t_d}\right]. \tag{A1}$$

A simple algebraic reorganization of Eq. (A1) leads to Eq. (1) in the manuscript:

$$\alpha = \frac{\frac{(N_{d-1} - N_{d-2})}{(t_{d-1} - t_{d-2})} + 2\frac{N_{d-1}}{t_d - t_{d-1}}}{2\frac{N_d}{t_d - t_{d-1}} + \frac{N_d - N_{d+1}}{t_{d+1} - t_d}}. \tag{A2}$$

To finalize this preprocessing step, the total tumor cell counts measured before the discontinuity are adjusted as follows: $\widehat{N}_{d-i} = N_{d-i}/\alpha$. Hence, the pre-discontinuity total tumor cell counts are proportionally reduced by a normalizing factor α to account for the fraction of cells that are lost during media handling. Figure A1 at the end of this appendix further illustrates this preprocessing procedure. Notice that this approach preserves the total tumor cells counts after media handling, since they are ultimately driving the ensuing tumor cell response (i.e., the main phenomenon that we investigate in our study and that the authors of Ref. [41] analyze in their work). The total MCF7 tumor cell count datasets used in our study were already preprocessed according to the just described by the authors of Ref. [41].

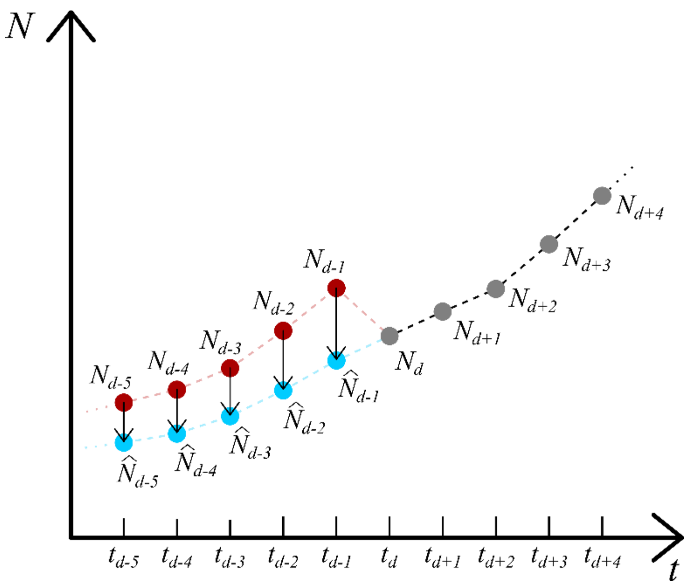

Figure A1. Data normalization to minimize the effect of accidental tumor cell removal during media handling. This procedure consists of adjusting the total tumor cell counts before the discontinuity introduced by an accidental cell loss during media handling at time t_d with a double objective: (1) smooth the local trend in total tumor cell dynamics in the vicinity of the discontinuity, and (2) correct the total tumor cell counts prior to the discontinuity by proportionally eliminating the fraction of cells that are accidentally removed during media handling. To this end, each measured total tumor cell count before the discontinuity N_{d-i} is divided by a strictly positive constant α, resulting in $\widehat{N}_{d-i}$, such that the slope at the discontinuity (i.e., from $\widehat{N}_{d-1}$ to N_d) equals the average of the slopes between the preceding and subsequent total tumor cell counts (i.e., from $\widehat{N}_{d-2}$ to $\widehat{N}_{d-1}$ and from N_d to N_{d+1}, respectively). In this figure, red points represent the total tumor cell counts that require adjustment due to cell loss during media handling at time t_d, blue points denote the corresponding corrected total tumor cell counts during this preprocessing step, and gray points represent total tumor cell counts after the discontinuity that remain unaltered. Dashed lines represent the slopes between total tumor cell counts, such that the red ones are modified by the data normalization to yield the blue ones. The adopted approach for data normalization was originally proposed in Ref. [41].

Appendix B. Model fitting

We fit our mathematical models of tumor cell growth and response to doxorubicin cytotoxic effects to each individual replicate dataset from each experiment listed in Table 1 in the main text. In particular, the single-dose model was fit to the time-course of total tumor cell count data of each individual replicate from Experiment 1 (i.e., varying doxorubicin concentrations, n = 60), while the multiple-dose model was fit to the time-course of total tumor cell count data of each individual replicate from Experiment 2 (i.e., varying inter-treatment interval between two consecutive doses of 75 nM doxorubicin, n = 108) and Experiment 3 (i.e., varying number of doses of 75 nM doxorubicin delivered at an inter-treatment interval of either 2 weeks or 2 days, n = 60 for each intertreatment interval).

We perform model fitting *via* a nonlinear least-squares method based on a trust-region reflective algorithm, as provided by *lsqnonlin* in MATLAB (R2020b). The function, step, and optimality tolerances were set at 10^{-6}, while the maximum number of function evaluations and iterations was set to 20,000. The parameter bounds and initial guesses were guided by the results from Howard *et al.* [41], and are summarized in Supplementary Tables S2, S8, S12, S16 and S17. Thus, our model fitting method aims at calculating the set of model parameters $\boldsymbol{p}$ minimizing an objective functional $J(\boldsymbol{p})$ given by

$$J(\boldsymbol{p}) = \frac{\sum_{i=1}^{n_f}\left(N(t_i,\boldsymbol{p}) - N_d(t_i)\right)^2}{\sum_{i=1}^{n_f}\left(N_d(t_i)\right)^2}\,, \tag{B1}$$

where $N(t_i,\boldsymbol{p})$ and $N_d(t_i)$ respectively denote the total tumor cell counts calculated with our model using a certain parameter set $\boldsymbol{p}$ and measured in the experiments of Ref. [41] at times t_i ($i = 1,2,\dots,n_f$; where n_f is the total number of available tumor cell counts for each replicate after data preprocessing). Hence, the right-hand side of Eq. (B1) consists of the sum of the squared differences between the model-evaluated total tumor cell count and the experimental measurements of total tumor cell counts normalized by the squared sum of the latter for each individual replicate dataset featuring measuring times t_i ($i = 1,2,\dots,n_f$).

To calculate the total tumor cell density $N(t_i,\boldsymbol{p})$ in Eq. (B1) during the application of the nonlinear least-square method outlined above, the ordinary differential equations in our mathematical models are solved by using a Runge-Kutta method as provided by *ode45* in MATLAB (R2020b). Recall that our models directly provide the total tumor cell number before the delivery of the first dose of doxorubicin *via* Eq. (2) in the main text. In the single-dose model, after the delivery of the first dose of doxorubicin we calculate the total tumor cell count by using Eq. (6) in the main text from the model solution of the tumor cell count in the surviving (S) and irreversibly damaged (D) cell compartments. Likewise, in the multiple-dose model we calculate the total tumor cell count *via* Eq. (11) in the main text from the model solution of the tumor cell count in the surviving (S) and each of the irreversibly damaged (D^i; $i = 1,2,\dots,n_d$) cell compartments after the delivery of n_d doses of doxorubicin.

Appendix C. Confidence intervals for model fits and parameters

The quality of the model fits presented in this manuscript was primarily assessed leveraging global metrics of quality of fit, such as the normalized root mean square error (NRMSE), the coefficient of determination (R^2), the Pearson correlation coefficient (PCC), and the concordance correlation coefficient (CCC). Additionally, we further assessed our model parameterizations and fits to each individual replicate dataset by utilizing 95% nonlinear regression parameter confidence intervals (CIs) and 95% nonlinear regression prediction CIs calculated using *nlparci* and *nlpredci* in MATLAB (R2020b), respectively. In brief, these two functions provide a centered 95% CI around each model parameter and fitted solution for each individual replicate, respectively, thereby enabling assessment of the level of uncertainty in these quantities.

This Appendix provides a collection of 95% CI results to illustrate the level of uncertainty in the model fits and corresponding parameterizations in the three experimental scenarios considered in this work. For each example, a figure illustrates the 95% CI in the model fit of total tumor cell counts and an accompanying table provides the parameter values and their corresponding 95% CI. Figure C1 and Table C1 correspond to an untreated control. Figures C2-C4 and Tables C2-C4 show examples of replicates treated with a single dose of varying concentrations of doxorubicin (Experiment 1). Figures C5-C7 and Tables C5-C7 correspond to replicates treated with two doses of 75 nM doxorubicin delivered at varying inter-treatment intervals (Experiment 2). Finally, Figures C8-C9 and Tables C8-C9 show examples of replicates treated with a varying number of 75 nM doses of doxorubicin every 2 days (Experiment 3), while Figures C10-C11 and Tables C10-C11 provide examples of replicates treated with a varying number of 75 nM doses of doxorubicin every 2 weeks (Experiment 3). As observed in these figures and tables, in general we obtain a minimal uncertainty in the model fits of total tumor cell counts and a low to moderate degree of uncertainty in the corresponding parameterizations across the experimental scenarios considered in this work.

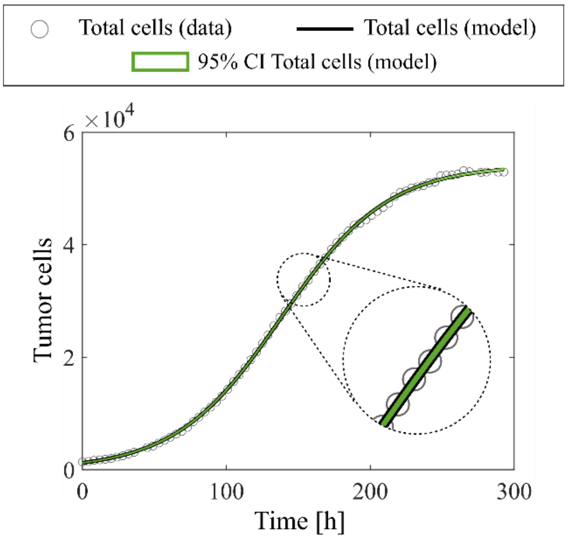

Figure C1. Example of 95% CI of the model fit of total tumor cell counts for an untreated replicate (i.e., 0 nM scenario in Experiment 1). The 95% CI (thin green lines) virtually overlaps on top of the model fit (thick black line), as shown in the zoomed detail.

Parameter (units)	Fitted value	95% CI
N_0 (cells)	1236	[1192, 1279]
g_0 (h^{-1})	0.027	[0.027, 0.028]
θ_u (cells)	54180	[53982, 54377]

Table C1. Parameter values and corresponding 95% CI for the model fitting represented in Figure C1. The logistic growth model was used in this case.

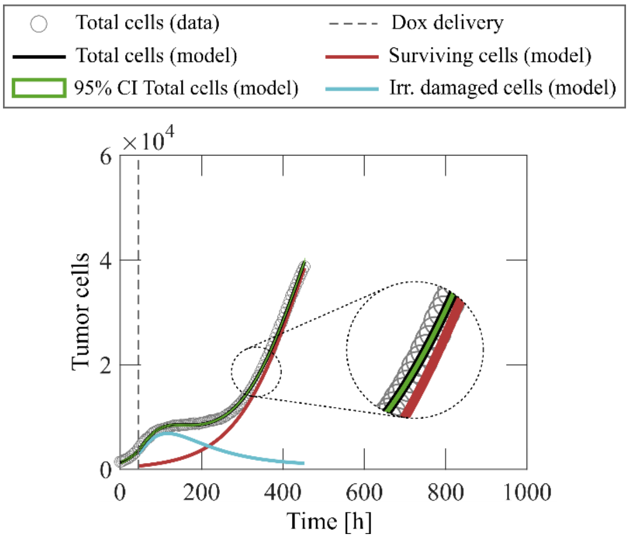

Figure C2. Example of 95% CI of the model fit of total tumor cell counts for a replicate treated with a single dose of 35 nM doxorubicin (Experiment 1). The 95% CI (thin green lines) virtually overlaps on top of the model fit (thick black line), as shown in the zoomed detail.

Parameter (units)	Fitted value	95% CI
N_0 (cells)	1248	[1011, 1486]
g_0 (h^{-1})	0.024	[0.019, 0.030]
f_s (-)	0.18	[0.089, 0.27]
g_s (h^{-1})	0.013	[0.011, 0.015]
g_d(h^{-1})	0.035	[0.027, 0.042]
k_d (h^{-1})	-0.0080	[-0.012, -0.0036]
γ_d (h^{-1})	0.023	[0.015, 0.031]
θ_{dox} (cells)	73338	[61330, 85346]

Table C2. Parameter values and corresponding 95% CI for the model fitting represented in Figure C2. The single dose model was used in this case.

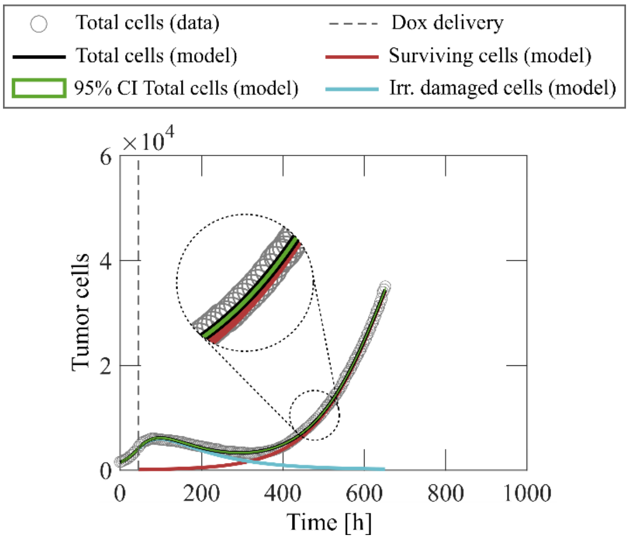

Figure C3. Example of 95% CI of the model fit of total tumor cell counts for a replicate treated with a single dose of 75 nM doxorubicin (Experiment 1). The 95% CI (thin green lines) virtually overlaps on top of the model fit (thick black line), as shown in the zoomed detail.

Parameter (units)	Fitted value	95% CI
N_0 (cells)	1475	[1292, 1658]
g_0 (h^{-1})	0.024	[0.021, 0.028]
f_s (-)	0.025	[0.021, 0.029]
g_s (h^{-1})	0.011	[0.011, 0.011]
g_d(h^{-1})	0.020	[0.015, 0.025]
k_d (h^{-1})	-0.0074	[-0.0083, -0.0065]
γ_d (h^{-1})	0.026	[0.020, 0.031]
θ_{dox} (cells)	72350	[66786, 77915]

Table C3. Parameter values and corresponding 95% CI for the model fitting represented in Figure C3. The single dose model was used in this case.

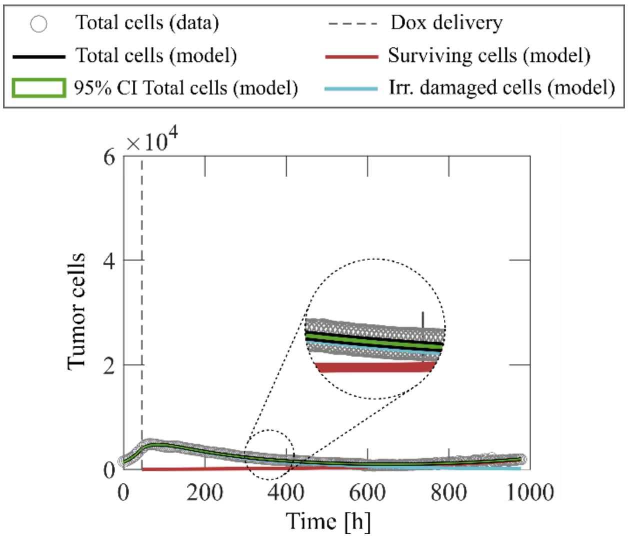

Parameter (units)	Fitted value	95% CI
N_0 (cells)	1403	[1295, 1512]
g_0 (h^{-1})	0.024	[0.022,0.027]
f_s (-)	0.013	[0.0082, 0.017]
g_s (h^{-1})	0.0040	[0.0036, 0.0044]
g_d(h^{-1})	0.012	[0.0080, 0.015]
k_d (h^{-1})	-0.0044	[-0.0046, -0.0041]
γ_d (h^{-1})	0.033	[0.026, 0.040]
θ_{dox} (cells)	68167	[-]

Figure C4. Example of 95% CI of the model fit of total tumor cell counts for a replicate treated with a single dose of 150 nM doxorubicin (Experiment 1). The 95% CI (thin green lines) virtually overlaps on top of the model fit (thick black line), as shown in the zoomed detail.

Table C4. Parameter values and corresponding 95% CI for the model fitting represented in Figure C4. The single dose model was used in this case. The value of θ_{Dox} was fixed according to the procedure described in the Methods section for this replicate dataset.

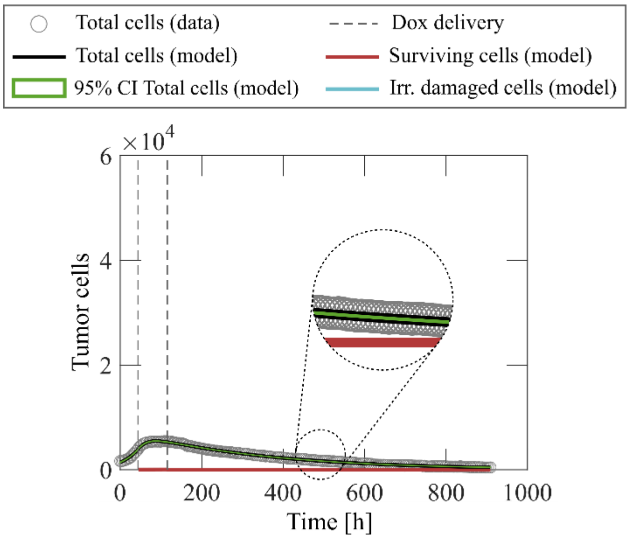

Parameter (units)	Fitted value	95% CI
N_0 (cells)	1411	[1349, 1472]
g_0 (h^{-1})	0.024	[0.023, 0.026]
f_s (-)	0.012	[-0.024, 0.049]
g_s (h^{-1})	0.0047	[-0.0025, 0.012]
g_d(h^{-1})	0.026	[0.022, 0.030]
k_d (h^{-1})	-0.0033	[-0.0034, -0.0033]
γ_d (h^{-1})	0.050	[0.043, 0.056]
θ_{dox} (cells)	53376	[-]

Figure C5. Example of 95% CI of the model fit of total tumor cell counts for a replicate treated with two doses of 75 nM doxorubicin delivered with an inter-treatment interval of 2 days (Experiment 2). The 95% CI (thin green lines) virtually overlaps on top of the model fit (thick black line), as shown in the zoomed detail.

Table C5. Parameter values and corresponding 95% CI for the model fitting represented in Figure C5. The multiple dose model with constant f_s and γ_d was used in this case. The value of θ_{Dox} was fixed according to the procedure described in the Methods section for this replicate dataset.

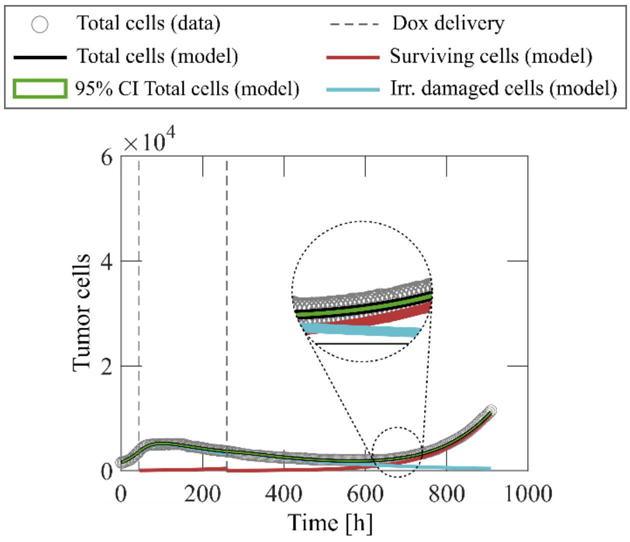

Figure C6. Example of 95% CI of the model fit of total tumor cell counts for a replicate treated with two doses of 75 nM doxorubicin delivered with an inter-treatment interval of 8 days (Experiment 2). The 95% CI (thin green lines) virtually overlaps on top of the model fit (thick black line), as shown in the zoomed detail.

Parameter (units)	Fitted value	95% CI
N_0 (cells)	1547	[748, 2346]
g_0 (h^{-1})	0.020	[0.013, 0.028]
f_s^1 (-)	0.018	[0.0091, 0.026]
f_s^2 (-)	0.068	[0.024, 0.11]
g_s (h^{-1})	0.0097	[0.0093, 0.010]
g_d(h^{-1})	0.020	[0.016, 0.023]
k_d (h^{-1})	-0.0038	[-0.0051, -0.0025]
γ_d^1 (h^{-1})	0.033	[0.025, 0.040]
γ_d^2 (h^{-1})	0.029	[0.016, 0.042]
θ_{dox} (cells)	53376	[-]

Table C6. Parameter values and corresponding 95% CI for the model fitting represented in Figure C6. The multiple dose model with varying f_s and γ_d was used in this case. The value of θ_{Dox} was fixed according to the procedure described in the Methods section for this replicate dataset.

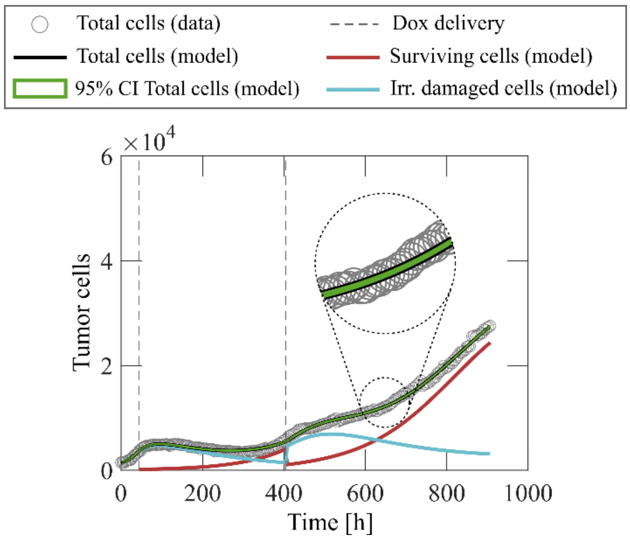

Figure C7. Example of 95% CI of the model fit of total tumor cell counts for a replicate treated with two doses of 75 nM doxorubicin delivered with an inter-treatment interval of 14 days (Experiment 2). The 95% CI (thin green lines) virtually overlaps on top of the model fit (thick black line), as shown in the zoomed detail.

Parameter (units)	Fitted value	95% CI
N_0 (cells)	1327	[1123, 1531]
g_0 (h^{-1})	0.024	[0.020, 0.028]
f_s^1 (-)	0.042	[0.0071, 0.077]
f_s^2 (-)	0.26	[0.13, 0.39]
g_s (h^{-1})	0.010	[0.0078, 0.013]
g_d(h^{-1})	0.019	[0.015, 0.023]
k_d (h^{-1})	-0.0048	[-0.0063, -0.0033]
γ_d^1 (h^{-1})	0.033	[0.023, 0.044]
γ_d^2 (h^{-1})	0.013	[0.010, 0.016]
θ_{dox} (cells)	37433	[33643, 41203]

Table C7. Parameter values and corresponding 95% CI for the model fitting represented in Figure C7. The multiple dose model with varying f_s and γ_d was used in this case.

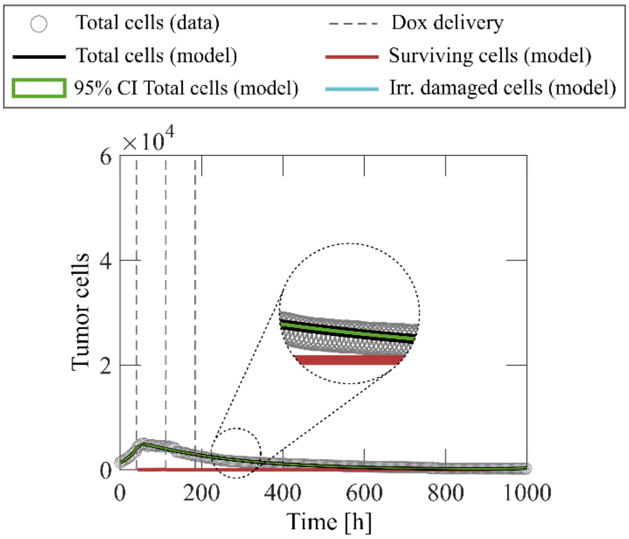

Figure C8. Example of 95% CI of the model fit of total tumor cell counts for a replicate treated with three doses of 75 nM doxorubicin delivered with an inter-treatment interval of 2 days (Experiment 3). The 95% CI (thin green lines) virtually overlaps on top of the model fit (thick black line), as shown in the zoomed detail.

**Parameter (units)**	**Fitted value**	**95% CI**
N_0 (cells)	1344	[1192, 1496]
g_0 (h^{-1})	0.028	[0.025, 0.032]
f_s (-)	0.0082	[-0.017, 0.034]
g_s (h^{-1})	0.012	[0.0020, 0.023]
g_d(h^{-1})	0.033	[0.011, 0.054]
k_d (h^{-1})	-0.0047	[-0.0049, -0.0045]
γ_d (h^{-1})	0.12	[0.057, 0.17]
θ_{dox} (cells)	59448	[-]

Table C8. Parameter values and corresponding 95% CI for the model fitting represented in Figure C8. The multiple dose model with constant f_s and γ_d was used in this case. The value of θ_{Dox} was fixed according to the procedure described in the Methods section for this replicate dataset.

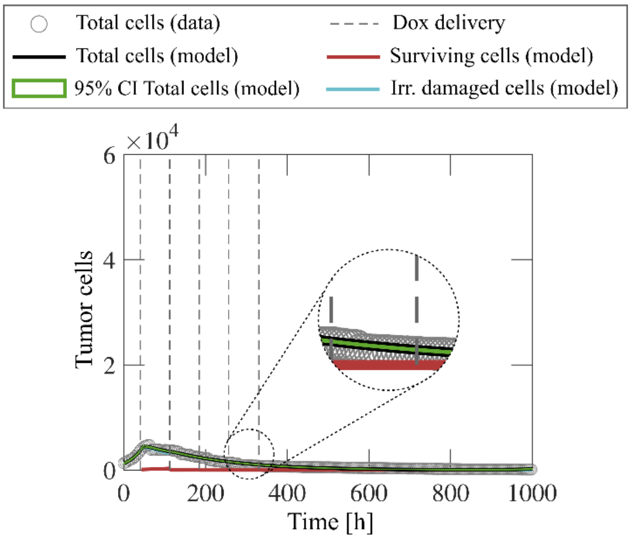

Figure C9. Example of 95% CI of the model fit of total tumor cell counts for a replicate treated with five doses of 75 nM doxorubicin delivered with an inter-treatment interval of 2 days (Experiment 3). The 95% CI (thin green lines) virtually overlaps on top of the model fit (thick black line), as shown in the zoomed detail.

**Parameter (units)**	**Fitted value**	**95% CI**
N_0 (cells)	1260	[1125, 1394]
g_0 (h^{-1})	0.028	[0.024, 0.032]
f_s (-)	0.032	[0.00019, 0.065]
g_s (h^{-1})	0.015	[0.0095, 0.020]
g_d(h^{-1})	0.050	[0.014, 0.086]
k_d (h^{-1})	-0.0061	[-0.0064, -0.0058]
γ_d (h^{-1})	0.18	[0.076, 0.28]
θ_{dox} (cells)	59448	[-]

Table C9. Parameter values and corresponding 95% CI for the model fitting represented in Figure C9. The multiple dose model with constant f_s and γ_d was used in this case. The value of θ_{Dox} was fixed according to the procedure described in the Methods section for this replicate dataset.

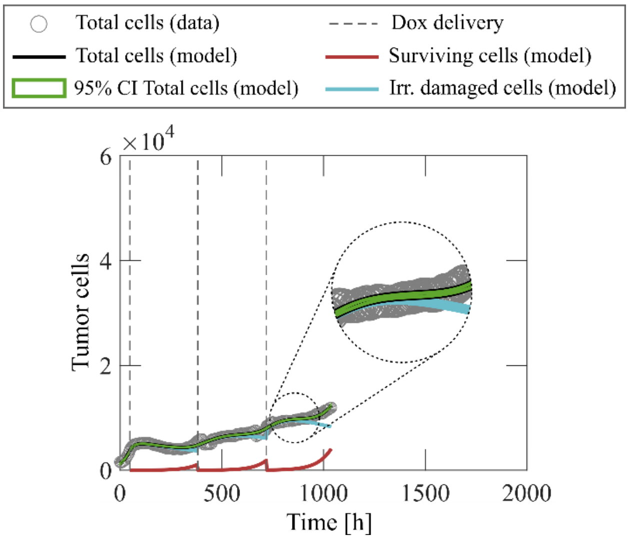

Figure C10. Example of 95% CI of the model fit of total tumor cell counts for a replicate treated with three doses of 75 nM doxorubicin delivered with an inter-treatment interval of 2 weeks (Experiment 3). The 95% CI (thin green lines) virtually overlaps on top of the model fit (thick black line), as shown in the zoomed detail.

Parameter (units)	Fitted value	95% CI
N_0 (cells)	1251	[1040, 1462]
g_0 (h^{-1})	0.024	[0.020, 0.028]
f_s^1 (-)	0.0010	[-0.0017, 0.0037]
f_s^2 (-)	0.0066	[-0.0090, 0.022]
f_s^3 (-)	0.016	[-0.016, 0.047]
g_s (h^{-1})	0.018	[0.011, 0.026]
g_d(h^{-1})	0.017	[0.013, 0.021]
k_d (h^{-1})	-0.0014	[-0.0019, -0.00076]
γ_d^1 (h^{-1})	0.044	[0.028, 0.060]
γ_d^2 (h^{-1})	0.010	[0.0085, 0.012]
γ_d^3 (h^{-1})	0.014	[0.010, 0.018]
θ_{dox} (cells)	62376	[-]

Table C10. Parameter values and corresponding 95% CI for the model fitting represented in Figure C10. The multiple dose model with varying f_s and γ_d was used in this case. The value of θ_{Dox} was fixed according to the procedure described in the Methods section for this replicate dataset.

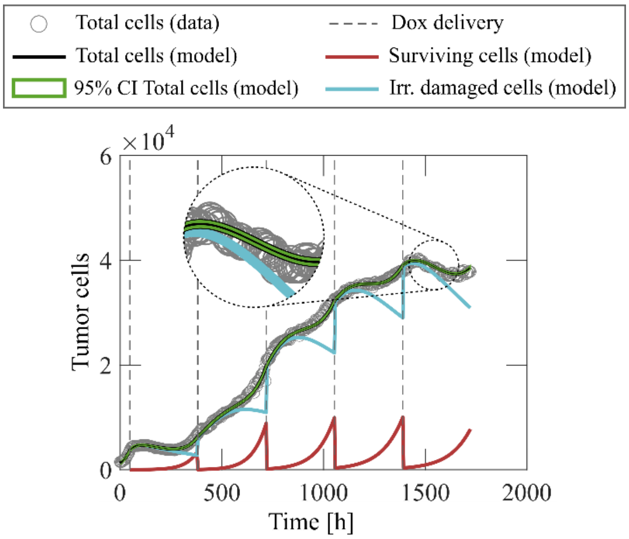

Figure C11. Example of 95% CI of the model fit of total tumor cell counts for a replicate treated with five doses of 75 nM doxorubicin delivered with an inter-treatment interval of 2 weeks (Experiment 3). The 95% CI (thin green lines) virtually overlaps on top of the model fit (thick black line), as shown in the zoomed detail.

Parameter (units)	Fitted value	95% CI
N_0 (cells)	1229	[905, 1553]
g_0 (h^{-1})	0.024	[0.018, 0.030]
f_s^1 (-)	0.0025	[-0.0015, 0.0066]
f_s^2 (-)	0.011	[-0.0045, 0.027]
f_s^3 (-)	0.017	[-0.0038, 0.038]
f_s^4 (-)	0.032	[-0.0033, 0.067]
f_s^5 (-)	0.033	[-0.0032, 0.070]
g_s (h^{-1})	0.019	[0.014, 0.023]
g_d(h^{-1})	0.013	[0.011, 0.014]
k_d (h^{-1})	-0.0023	[-0.0035, -0.0011]
γ_d^1 (h^{-1})	0.033	[0.019, 0.047]
γ_d^2 (h^{-1})	0.0072	[0.0063, 0.0081]
γ_d^3 (h^{-1})	0.0092	[0.0076, 0.011]
γ_d^4 (h^{-1})	0.010	[0.0078, 0.012]
γ_d^5 (h^{-1})	0.014	[0.0097, 0.019]
θ_{dox} (cells)	77565	[65746, 89384]

Table C11. Parameter values and corresponding 95% CI for the model fitting represented in Figure C11. The multiple dose model with varying f_s and γ_d was used in this case.

Appendix D. Analysis of f_s^2 and γ_d^2 distributions in Experiment 2

We used two-sided Wilcoxon rank sum tests to statistically compare the distributions of f_s^2 and γ_d^2 values obtained across the inter-treatment interval scenarios in Figure 7 of the main text. We remark that, for inter-treatment intervals between 0 and 6 days, we used the multiple-dose model with constant parameters (which assumes $f_s^1 = f_s^2$ and $\gamma_d^1 = \gamma_d^2$), while, for inter-treatment intervals between 8 and 16 days, we leveraged the multiple-dose model with varying parameters (i.e., varying f_s and γ_d). Tables D1 and D2 provide the p-values resulting from this analysis over the f_s^2 and γ_d^2 distributions. As noted in the Results section in the main text, the analysis of the distributions of the f_s^2 values obtained across the different inter-treatment interval scenarios in Experiment 2 shows a tendency towards a larger f_s^2 value for larger inter-treatment intervals, which is suggestive of increased chemoresistance in the surviving cell compartment. However, this trend becomes less clear among the longest intervals considered in Experiment 2 (i.e., > 8 days), for which f_s^2 appears to plateau at a value between 0.10 and 0.15. Additionally, the analysis of the distributions of the γ_d^2 values obtained across the inter-treatment intervals shows that there is a decreasing trend between the 0-day and the 14-day inter-treatment interval scenario, which suggests an increase in the chemoresistance of the irreversibly-damaged cells (i.e., they transition more slowly from proliferation to drug-induced cell death); although this tendency is reverted for the 16-day inter-treatment interval.

	0 days	2 days	4 days	6 days	8 days	10 days	12 days	14 days	16 days
0 days	-	**3.84×10^{-4}**	**4.69×10^{-5}**	**7.66×10^{-5}**	**3.66×10^{-5}**	**3.66×10^{-5}**	**3.66×10^{-5}**	**3.66×10^{-5}**	**3.66×10^{-5}**
2 days	-	-	**0.0043**	**0.0073**	**5.92×10^{-4}**	**4.78×10^{-4}**	**3.08×10^{-4}**	**3.08×10^{-4}**	**3.84×10^{-4}**
4 days	-	-	-	0.4705	**1.56×10^{-4}**	**1.56×10^{-4}**	**4.69×10^{-5}**	**3.84×10^{-4}**	**4.69×10^{-5}**
6 days	-	-	-	-	**5.92×10^{-4}**	**3.08×10^{-4}**	**4.69×10^{-5}**	**7.31×10^{-4}**	**4.69×10^{-5}**
8 days	-	-	-	-	-	0.5444	**0.0404**	0.8399	0.0999
10 days	-	-	-	-	-	-	0.1260	0.6650	0.3708
12 days	-	-	-	-	-	-	-	0.1410	0.5444
14 days	-	-	-	-	-	-	-	-	0.1572
16 days	-	-	-	-	-	-	-	-	-

Table D1. P-values from two-sided Wilcoxon rank sum tests comparing the values of f_s^2 obtained for every distinct combination of the varying inter-treatment interval datasets (n = 12 per tested interval; Experiment 2 in Table 1 of the main text). The multiple-dose model with constant parameters was used for inter-treatment intervals from 0 to 6 days, while the model version with varying f_s and γ_d was leveraged for inter-treatment intervals from 8 to 16 days. Values bolded in red indicate $p < 0.05$.

	0 days	2 days	4 days	6 days	8 days	10 days	12 days	14 days	16 days
0 days	-	0.6650	0.0999	**0.0024**	**0.0011**	**1.56×10^{-4}**	**6.01×10^{-5}**	**3.66×10^{-5}**	**7.66×10^{-5}**
2 days	-	-	**0.0043**	**3.66×10^{-5}**	**7.66×10^{-5}**	**3.66×10^{-5}**	**3.66×10^{-5}**	**3.66×10^{-5}**	**3.66×10^{-5}**
4 days	-	-	-	**0.0011**	**7.31×10^{-4}**	**3.66×10^{-5}**	**3.66×10^{-5}**	**3.66×10^{-5}**	**7.66×10^{-5}**
6 days	-	-	-	-	0.2855	**0.0073**	**3.84×10^{-4}**	**3.66×10^{-5}**	**3.84×10^{-4}**
8 days	-	-	-	-	-	0.0885	**0.0035**	**5.92×10^{-4}**	**0.0086**
10 days	-	-	-	-	-	-	**0.0351**	**0.0017**	0.1572
12 days	-	-	-	-	-	-	-	0.2855	0.3708
14 days	-	-	-	-	-	-	-	-	**0.0351**
16 days	-	-	-	-	-	-	-	-	-

Table D2. P-values from two-sided Wilcoxon rank sum tests comparing the values of γ_d^2 obtained for every distinct combination of the varying inter-treatment interval datasets (n = 12 per tested interval; Experiment 2 in Table 1 of the main text). The multiple-dose model with constant parameters was used for inter-treatment intervals from 0 to 6 days, while the model version with varying f_s and γ_d was leveraged for inter-treatment intervals from 8 to 16 days. Values bolded in red indicate p < 0.05.

To gain additional insight into the changes in f_s^2 and γ_d^2 as the inter-treatment interval increases, Figure D1 further reports the distributions of f_s^1, f_s^2, γ_d^1, and γ_d^2 obtained in all inter-treatment interval scenarios leveraging only the multiple-dose model with varying parameters. We remark that, although significant differences between f_s^1 and f_s^2 as well as γ_d^1 and γ_d^2 are obtained for inter-treatment intervals between 0 and 6 days (see Figure D1), the analysis of the fitting error reported in Figure 5 showed that the multiple-dose model with constant parameterization was not inferior than the multiple-dose model with varying f_s and γ_d, while using a comparatively smaller parameter set (i.e., the constant parameter formulation assumes $f_s^1 = f_s^2$ and $\gamma_d^1 = \gamma_d^2$). This means that the differences in f_s and γ_d values do not produce a significant impact in the ability of the multiple-dose model to recapitulate the dynamics of the MCF-7 tumor cell populations treated with inter-treatment intervals from 0 to 6 days in Experiment 2. Consequently, from a modeling selection perspective (e.g., see Ref. [22]), we adopted the constant parameterization for inter-treatment intervals between 0 and 6 days. Additionally, notice that the global trends for the changes in f_s^2 and γ_d^2 values across the inter-treatment interval scenarios reported in the Results section also hold when only the multiple-dose model with varying parameters is used to fit the datasets from Experiment 2. First, there is an increasing trend towards larger f_s^2 as the inter-treatment interval in expanded, although this tendency appears to plateau for the longest intervals (i.e., > 8 days). Second, the values of γ_d^2 exhibit a decreasing trend between the 0-day and 14-day inter-treatment scenario, but this is reverted once the inter-treatment interval is lengthened to 16 days. Thus, these results confirm that the multiple-dose model with constant parameters is an acceptable and efficient surrogate to investigate the response of MCF-7 cell populations to doxorubicin regimens featuring two consecutive 75 nM doses delivered at inter-treatment intervals between 0 and 6 days. Nevertheless, as noted in the Discussion section, future studies should extend our analyses in MCF-7 replicates treated with 2 consecutive doses of doxorubicin with different dosages and also exploring inter-treatment intervals larger than 16 days.

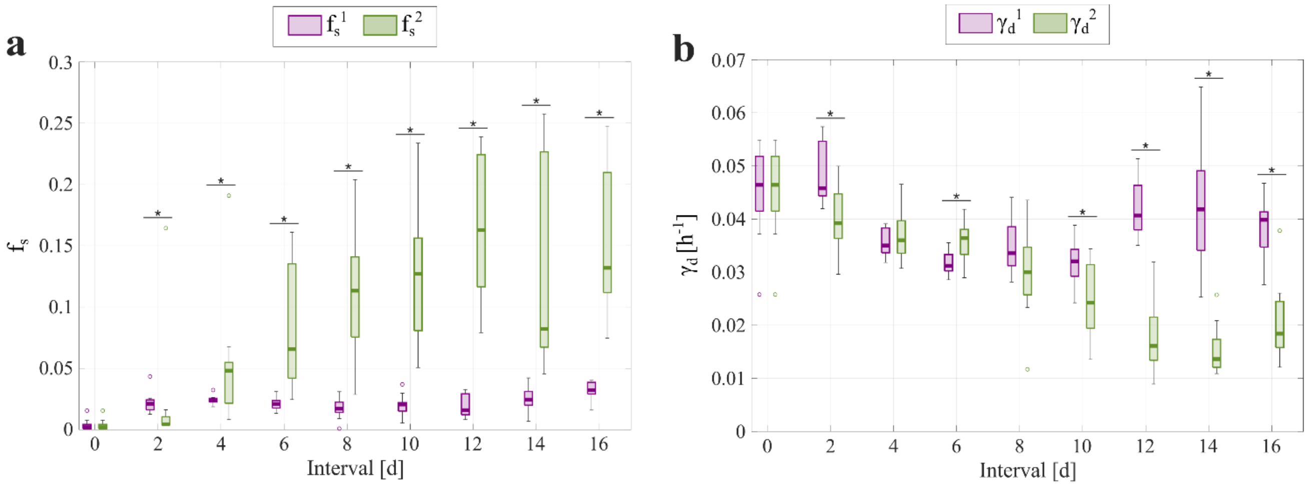

Figure D1. Comparison of the f_s and γ_d distributions obtained from fitting the multiple-dose model with varying parameters to the experimental data for different inter-treatment intervals. The parameter distributions are represented as boxplots and were obtained from fitting the multiple-dose model with varying parameters to the different inter-treatment interval datasets from Experiment 2 (Table 1). Outliers are represented with circles. Panel (a) shows the distributions for the fraction of surviving cells after each dose (f_s^1, f_s^2). Panel (b) shows the distributions for the doxorubicin-induced death delay rate of the irreversibly-damaged tumor cells (γ_d^1, γ_d^2). The boxplots of the parameter values related to the first dose are shown in purple (i.e., f_s^1, γ_d^1), while the boxplots of the parameter values related to the second dose are represented in green (i.e., f_s^2, γ_d^2). The results in this figure show that the fraction of surviving tumor cells and the death delay rate of irreversibly-damaged cells after the second doxorubicin dose (i.e., f_s^2 and γ_d^2, respectively) tend to be significantly different than the corresponding values after the first dose (i.e., f_s^1 and γ_d^1, respectively), especially for the longer inter-treatment intervals. Panel (a) further shows that there is an increasing trend towards larger f_s^2 as the inter-treatment interval in expanded, although this tendency appears to plateau for the longest intervals (i.e., > 8 days). Additionally, in panel (b) the values of γ_d^2 exhibit a decreasing trend between the 0-day and 14-day inter-treatment scenario, but this is reverted once the inter-treatment interval is lengthened to 16 days. These changes in f_s and γ_d after the second dose also suggest an increasingly poorer tumor control with a longer inter-treatment interval. An asterisk (*) indicates $p < 0.05$ in two-sided Wilcoxon rank sum tests comparing the distributions of the two f_s and γ_d values obtained for each inter-treatment interval.
